# Calcium Hydride Cation Dimer Catalyzed Hydrogenation of Unactivated 1‐Alkenes and H_2_ Isotope Exchange: Competitive Ca−H−Ca Bridges and Terminal Ca−H Bonds

**DOI:** 10.1002/chem.202202602

**Published:** 2022-11-14

**Authors:** Zheng‐Wang Qu, Hui Zhu, Stefan Grimme

**Affiliations:** ^1^ Mulliken Center for Theoretical Chemistry University of Bonn Beringstr. 4 53115 Bonn Germany

**Keywords:** 1-alkene, calcium hydride complexes, homogenous catalysis, hydrogenation, isotope exchange

## Abstract

Recently, it was shown that the double Ca−H−Ca bridged calcium hydride cation dimer complex [LCaH_2_CaL]^2+^ (macrocyclic ligand L=NNNN‐tetradentate Me_4_TACD) exhibited remarkable activity in catalyzing the hydrogenation of unactivated 1‐alkenes as well as the H_2_ isotope exchange under mild conditions, tentatively via the terminal Ca−H bond of cation monomer LCaH^+^. In this DFT mechanistic work, a novel substrate‐dependent catalytic mechanism is disclosed involving cooperative Ca−H−Ca bridges for H_2_ isotope exchange, competitive Ca−H−Ca bridges and terminal Ca−H bonds for anti‐Markovnikov addition of unactivated 1‐alkenes, and terminal Ca−H bonds for Markovnikov addition of conjugation‐activated styrene. THF‐coordination plays a key role in favoring the anti‐Markovnikov addition while strong cation‐π interactions direct the Markovnikov addition to terminal Ca−H bonds.

## Graphical abstract

**Calcium hydride cation dimer catalysis**: Dispersion‐corrected DFT calculations reveal that the same double Ca−H−Ca bridged calcium hydride cation dimer catalyst [LCaH_2_CaL]^2+^⋅THF shows a novel substrate‐dependent catalytic activity, involving cooperative Ca−H−Ca bridges for efficient H_2_ isotope exchange, terminal Ca−H bond for the Markovnikov addition of conjugation‐activated styrene, and competitive Ca−H−Ca bridges and terminal Ca−H bonds for the anti‐Markovnikov addition of unactivated 1‐alkenes.

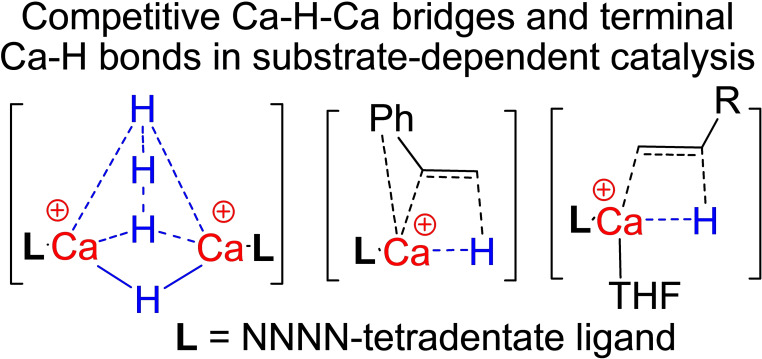

## Introduction

**Scheme 1:**
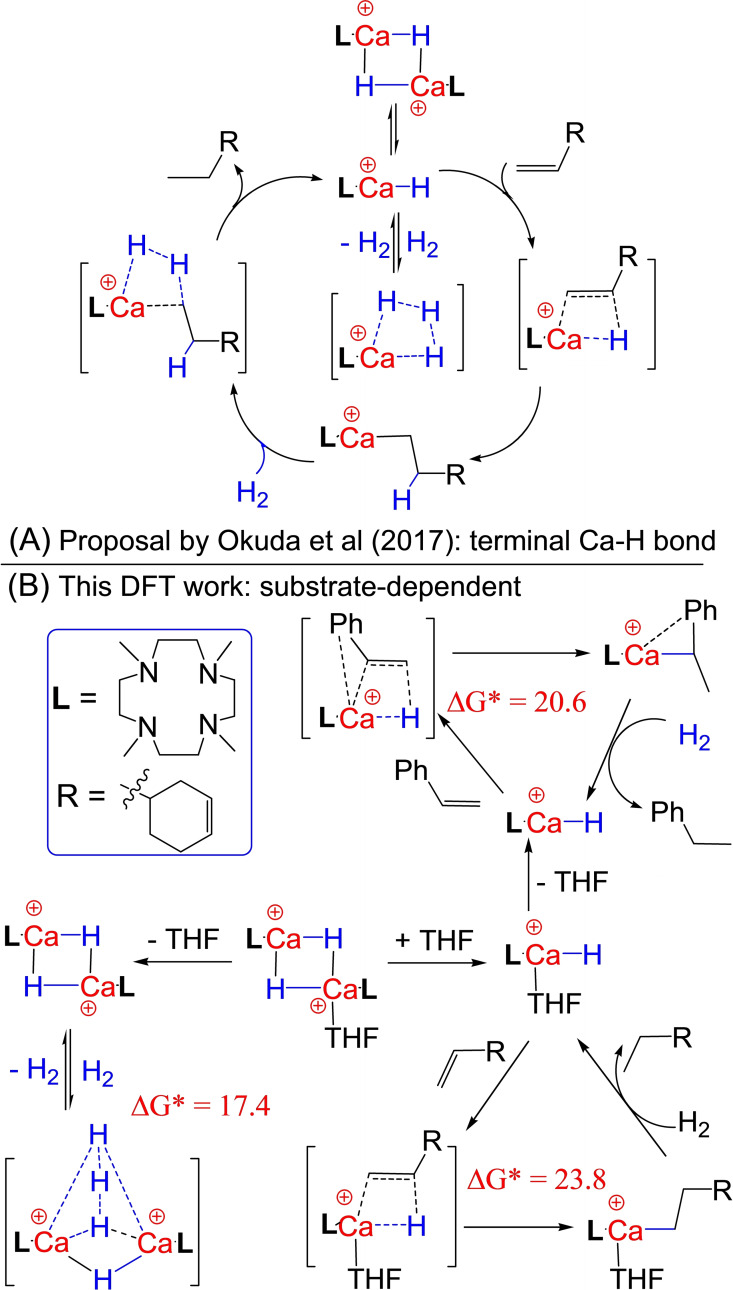
Catalytic cycle for the hydrogenation of 1‐alkenes and H_2_ isotope exchange with calcium hydride cation dimer catalyst [LCaH_2_CaL]^2+^: (A) via the terminal Ca−H bond of cation monomer LCaH^+^ as recently proposed by Okuda et al.; (B) via competitive Ca‐H−Ca bridges and terminal Ca−H bonds in a highly substrate‐dependent mechanism in this DFT mechanistic work.

While the homogeneous hydrogenation of alkenes catalyzed by transition‐metal complexes^[1]^ is well‐established, “frustrated” Lewis pair (FLP)^[2]^ and main‐group‐metal^[3]^ hydrogenation catalysts are mostly limited to activated (conjugated) C=C double bonds. Rare examples are the hydrogenation of alkenes catalyzed by borane HB(C_6_F_5_)_2_ catalyst under elevated heating at 140 °C in benzene,^[4]^ by the super‐bulky neutral calcium hydride monomer complexes of TpCaH (ligand Tp=NNN‐tridentate hydrotris(3‐adamantyl‐5‐isopropyl‐pyrazolyl)borate) under moderate heating at 40 °C in benzene^[5]^ and by the non‐THF‐coordinated neutral *β*‐diketiminato calcium hydride dimer complex, [(BDI)CaH_2_Ca(BDI)] (ligand BDI = HC{(Me)CN‐2,6‐*i*‐Pr_2_C_6_H_3_}_2_.^[6]^ Recently, Okuda et al. reported that the double Ca−H−Ca bridged, THF‐coordinated calcium hydride cation dimer **1^2+^** ⋅THF ([LCaH_2_CaL]^2+^⋅THF, ligand L = 1,4,7,10‐tetramethyl‐1,4,7,10‐tetraazacyclododecane)^[7]^ showed remarkable catalytic activity in the hydrogenation of unactivated 1‐alkenes under moderate heating at 60 °C in THF solution.^[8]^ The use of larger macrocyclic NNNNN‐pentadentate ligand of 1,4,7,10,13‐pentamethyl‐1,4,7,10,13‐pentaazacyclopentadecane) led to even improved catalytic activity in such catalysis.^[3f]^ It was proposed^[8]^ that the cation dimer **1^2+^** ⋅THF may dissociate into reactive cation monomer species **1 m^+^** LCaH^+^ to induce facile 1‐alkene addition to terminal Ca−H bonds, followed by H_2_ splitting over the resultant Ca−C bonds to release the hydrogenated alkane product (Scheme 1A). The terminal Ca−H bond of **1 m^+^** was also suggested to induce very facile H_2_ isotope exchange within 5 minutes at room temperature.^[8]^ The same catalytic hydrogenation mechanism was also proposed with the neutral calcium hydride monomer catalysts.[^5^, ^9^]

Despite the high reactivity of terminal Ca−H bonds expected for calcium hydride monomer complexes stabilized by bulky ligands,[^5^, ^8^] dimers or higher calcium hydride oligomers are usually formed via more stable Ca−H−Ca bridges that may react via a cooperative FLP‐like reactivity as observed for similar M−H−M bridges (metal M=Li, K, Al).^[10]^ In view of the remarkable activity of the Ca−H−Ca bridged cation dimer **1^2+^** ⋅THF in catalyzing the hydrogenation of unactivated 1‐alkenes, it is still highly desirable to clarify the role of potential Ca−H−Ca bridges, terminal Ca−H bonds and coordinating THF molecules in the actual catalytic hydrogenation of 1‐alkenes and H_2_ isotope exchange and reactions.^[8]^ Indeed, our extensive DFT calculations disclose the highly substrate‐dependent catalytic activity of the same cation dimer catalyst **1^2+^** ⋅THF, involving cooperative Ca−H−Ca bridges of **1^2+^** for H_2_ isotope exchange, competitive Ca−H−Ca bridges of **1^2+^** and the terminal Ca−H bond of **1 m^+^** ⋅THF for the anti‐Markovnikov addition of unactivated 1‐alkenes, and the terminal Ca−H bond of **1 m^+^** for the Markovnikov addition of conjugation‐activated styrene, respectively (Scheme 1B).

## Results and Discussion

To gain deep mechanistic insight into the cation dimer **1^2+^** ⋅THF catalyzed hydrogenation of 1‐alkenes and H_2_ isotope exchange reactions, state‐of‐the‐art dispersion‐corrected DFT calculations are performed at the PW6B95‐D3/def2‐QZVP+COSMO‐RS//TPSS‐D3/def2‐TZVP+COSMO level in THF solution (see below for computational details), and final free energies (at 298.15 K and 1 M concentration) are used in our discussion unless specified otherwise. In THF solution, the weakly (or non‐) coordinating counter‐anions BAr_4_^−^ should be solvated into separated ions, and thus not considered further. Consistent with experiment,^[7]^ our DFT calculations show that the THF coordination to **1^2+^** is indeed −2.8 kcal/mol exergonic, with a decisive stabilizing dispersion contribution of −7.6 kcal/mol otherwise the coordination should be 4.8 kcal/mol endergonic and thus thermodynamically unstable. For comparison, the THF coordination to the cation monomer **1 m^+^** is only −1.5 kcal/mol exergonic with a smaller dispersion contribution of −4.2 kcal/mol. The formation of double Ca−H−Ca bridged **1^2+^** from two **1 m^+^** monomers is −18.4 kcal/mol exergonic again with a sizable dispersion contribution of −6.2 kcal/mol. The THF coordination to **1^2+^** ⋅THF smoothly leads to two **1 m^+^** ⋅THF cation monomers that is 15.3 kcal/mol endergonic, indicating a rapid dimer‐to‐monomer equilibrium even at room temperature (see Supporting Information Table S1).

In two related mechanistic studies on 1‐alkene hydrogenation reactions, the THF‐coordinated [(THF)(BDI)CaH]_2_ ^[9]^ and the non‐THF‐coordinated [(BDI)CaH_2_Ca(BDI)]^[6]^ calcium hydride dimers were used as catalyst. Dispersion‐uncorrected DFT calculations suggested that the dimer [(THF)(BDI)CaH]_2_ is about 7.7 kcal/mol higher in free energy than two (THF)(BDI)CaH monomers in benzene solution,^[9]^ in contrast to previous experiment^[3b]^ and our dispersion‐corrected DFT results (−18.2 kcal/mol lower than two monomers). Moreover, dispersion‐uncorrected DFT calculations suggested that the dimer [(BDI)CaH_2_Ca(BDI)] is 40.4 kcal/mol lower in enthalpy than two (BDI)CaH monomers in gas‐phase,^[6]^ which however should be further enhanced by 17.5 kcal/mol and decreased by 26.8 kcal/mol due to dispersion interactions and solvation in benzene, respectively, according to our dispersion‐corrected DFT calculations. It is thus crucial to include both dispersion corrections and suitable solvation model in modeling such catalytic reactions in solution.

**Figure 1:**
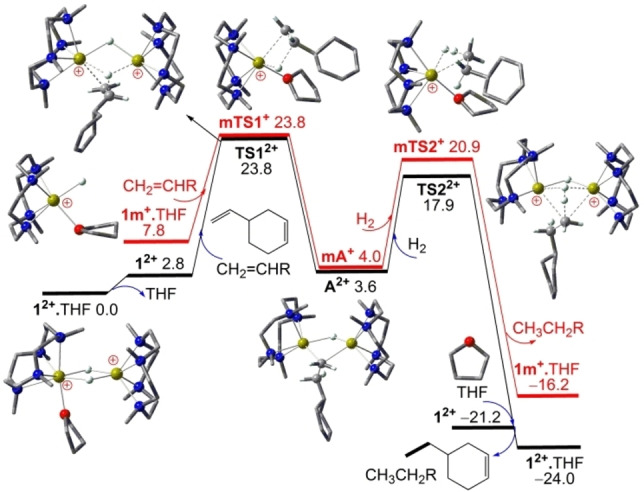
DFT computed free energy profile (in kcal/mol, at 298 K and 1 M) for the **1^2+^** ⋅THF catalyzed hydrogenation of unactivated 1‐alkene CH_2_=CHR (R=3‐cyclohexenyl) either via cation monomer **1 m^+^** ⋅THF (in red line) or double Ca−‐H−Ca bridged cation dimer **1^2+^** (in black line).

As shown in Figure 1, starting from the stable dinuclear complex **1^2+^** ⋅THF, the anti‐Markovnikov addition of unactivated 1‐alkene CH_2_=CHR (R=3‐cyclohexenyl) to a Ca−H−Ca bridge of **1^2+^** after THF‐elimination is 3.6 kcal/mol endergonic over a sizable free energy barrier of 23.8 kcal/mol (via transition state **TS1^2+^**) to selectively form the dinuclear complex **A^2+^** [LCaHR'CaL]^2+^ containing both a Ca−H−Ca and a Ca−R’−Ca (alkyl R’=CH_2_CH_2_R) bridge. The alternative Markovnikov addition with the hydride added to the terminal alkene carbon is kinetically 7.6 kcal/mol less favorable (via **TS1a^2+^**, see Supporting Information Figure S1 and Table S1). The subsequent H_2_ addition over the Ca−R’−Ca bridge of **A^2+^** is highly exergonic and kinetically 5.8 kcal/mol more favorable (via **TS2^2+^**) to form the hydrogenated product CH_3_CH_2_R along with regenerated **1^2+^** ⋅THF after THF coordination. Such a catalytic 1‐alkene hydrogenation is thus −23.7 kcal/mol exergonic over a sizable barrier of 23.8 kcal/mol involving anti‐Markovnikov alkene addition to cooperative Ca−H−Ca bridges.

On the other hand, via the THF‐coordinated cation monomer **1 m^+^** ⋅THF that is 7.8 kcal/mol higher in free energy than **1^2+^** ⋅THF, the anti‐Markovnikov addition of unactivated CH_2_=CHR to the terminal Ca−H bond is still 4.0 kcal/mol endergonic but over nearly the same free energy barrier of 23.8 kcal/mol (via **mTS1^+^**) to selectively form the calcium alkyl complex **mA^+^** [LCaHR’⋅THF]^+^, with the alternative Markovnikov addition being kinetically 6.0 kcal/mol less favorable (via **mTS1a^+^**, see Supporting Information Figure S1 and Table S1). The subsequent hydrogenolysis of the Ca−C bond of **mA^+^** with H_2_ is highly exergonic and kinetically 2.9 kcal/mol more favorable (via **mTS2^+^**) to form the hydrogenated product CH_3_CH_2_R along with regenerated **1 m^+^** ⋅THF. When cyclohexene is used as an internal alkene substrate in **1 m^+^** ⋅THF catalyzed hydrogenation, a 3.3 kcal/mol higher barrier (via **cTS1^+^**, see Supporting Information Table S1) is found for the anti‐Markovnikov addition to the terminal Ca−H bond, consistent with the experimentally observed selective hydrogenation of terminal alkene.^[8]^

Interestingly, after THF elimination, the terminal Ca−H bond of solvent‐free **1 m^+^** is kinetically 0.7 kcal/mol less reactive (via **mTS10^+^**, see Supporting Information Figure S1 and Table S1) than that of **1 m^+^** ⋅THF towards CH_2_=CHR addition, mainly due to a less Lewis‐basic hydride (Mulliken atomic charges: −0.16 *e* in **1 m^+^** vs. −0.53 *e* in **1 m^+^** ⋅THF). Together with the positive THF affinity of **1 m^+^**, this leads to a 2.1 kcal/mol higher barrier of 25.9 kcal/mol for CH_2_=CHR addition without the coordinating THF. Interestingly, despite nearly the same free energy barriers (23.8 kcal/mol) at 298 K for the competitive mechanisms via **TS1^2+^** and **mTS1^+^**, our DFT calculations show that the former via the solvent‐free dimer **1^2+^** becomes kinetically 1.4 kcal/mol more favorable than the latter via the THF‐coordinated **1 m^+^** ⋅THF (barriers: 24.2 versus 25.6 kcal/mol) upon heating at 60 °C (333 K) in experiment,^[8]^ mainly due to entropy‐favored THF‐elimination from **1^2+^** ⋅THF to reach the solvent‐free **TS1^2+^**. In contrary to the usual intuition that the dimer‐to‐monomer conversion should be favored upon heating due to favorable entropy effects, our DFT calculations show that the equilibrium of **1^2+^** ⋅THF+THF→**1 m^+^** ⋅THF+**1 m^+^** ⋅THF in solution is hardly affected by such temperature change due to negligible entropy difference with an unchanged number of molecules on both sides (see Supporting Information Table S1).

**Figure 2:**
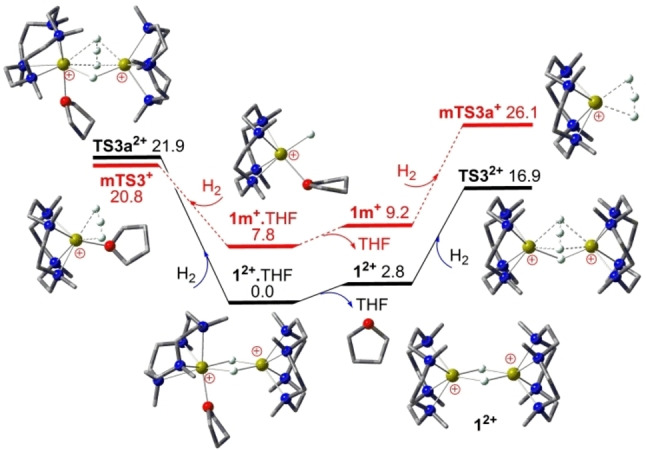
DFT computed free energy profile (in kcal/mol, at 298 K and 1 M) for the **1^2+^** ⋅THF catalyzed H_2_ isotope exchange either via cation monomer **1 m^+^** ⋅THF (in red line) or solvent‐free cation dimer **1^2+^** (in black line).

As seen in Figure 2, very facile H−H−H type H_2_ isotope exchange may occur via one of two cooperative Ca−H−Ca bridges of the solvent‐free cation dimer **1^2+^** over a low barrier of 16.9 kcal/mol (via **TS3^2+^**) after THF‐elimination from stable **1^2+^** ⋅THF catalyst. Interestingly, three exchanging hydrogen atoms are placed evenly between two calcium ions and perpendicular to the remaining Ca−H−Ca bridge, suggesting potentially strong cooperative effects. With the coordinating THF within **1^2+^** ⋅THF, similar H_2_ isotope exchange (via **TS3a^2+^**) becomes kinetically 4.5 kcal/mol less favorable. On the other hand, facile H_2_ isotope exchange may also occur via terminal Ca−H bonds that are more reactive but higher in free energy than Ca−H−Ca bridges. Indeed, the THF‐coordinated cation monomer **1 m^+^** ⋅THF is intrinsically 3.9 kcal/mol (via **mTS3^+^**) more reactive than **1^2+^** for H_2_ isotope exchange but 7.8 kcal/mol higher in free energy, eventually leading to a 3.9 kcal/mol higher barrier. Interestingly, the solvent‐free cation monomer **1 m^+^** with a less Lewis‐basic hydride turns out to be intrinsically 3.9 kcal/mol less reactive than **1 m^+^** ⋅THF in mediating H_2_ isotope exchange, which is further disfavored by its positive THF affinity of 1.4 kcal/mol. It is thus clear that very efficient H_2_ isotope exchange observed at room temperature^[8]^ is actually catalyzed by cooperative Ca−H−Ca bridges of solvent‐free **1^2+^** over a low barrier of 16.9 kcal/mol (via **TS3^2+^**) rather than recently proposed terminal Ca−H bonds.

**Figure 3:**
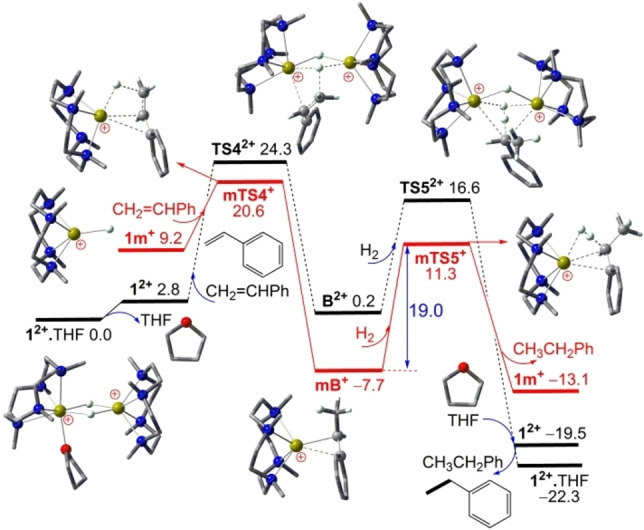
DFT computed free energy profile (in kcal/mol, at 298 K and 1 M) for the **1^2+^** ⋅THF catalyzed hydrogenation of conjugation‐activated styrene CH_2_=CHPh via solvent‐free cation monomer **1 m^+^** (in red line) or solvent‐free cation dimer **1^2+^** (in black line).

As shown in Figure 3, starting from the stable cation dimer catalyst **1^2+^** ⋅THF, the Markovnikov addition of styrene (CH_2_=CHPh) as a typical π‐conjugation‐activated 1‐alkene to a cooperative Ca−H−Ca bridge of **1^2+^** after THF‐elimination is 0.2 kcal/mol endergonic over a sizable barrier of 24.3 kcal/mol (via **TS4^2+^**), selectively leading to the dinuclear calcium‐complex **B^2+^** [LCaHR“CaL]^2+^ containing both a Ca−H−Ca and a Ca−R”−Ca (alkyl R“=CH(CH_3_)Ph) bridge. Note that the hydride is now added to the terminal rather than the inner alkene carbon as directed by strong Ca^+^…Ph cation‐π interactions; the anti‐Markovnikov addition encounters a 2.7 kcal/mol higher barrier (via **TS4a^2+^**; see Supporting Information Figure S2 and Table S1) and thus is kinetically disfavored. The subsequent hydrogenolysis of the Ca−R”−Ca bridge of **B^2+^** with H_2_ is highly exergonic and kinetically 7.7 kcal/mol more favorable (via **TS5^2+^**) to form the hydrogenated product CH_3_CH_2_Ph along with regenerated catalyst **1^2+^** ⋅THF after THF‐coordination. Such dimeric catalytic mechanism is −22.3 kcal/mol exergonic over a sizable barrier of 24.3 kcal/mol involving Markovnikov 1‐alkene addition to cooperative Ca−H−Ca bridges.

Interestingly, starting from the stable complex **1^2+^** ⋅THF, the Markovnikov styrene addition to the terminal Ca−H bond of the solvent‐free cation monomer **1 m^+^** (via **mTS4^+^**) is now kinetically very efficient and again directed by strong Ca^+^…Ph ion‐π interaction, which is now −7.7 kcal/mol exergonic over a moderate barrier of 20.6 kcal/mol to selectively form the calcium benzyl complex **mB^+^** LCaR“^+^. The alternative anti‐Markovnikov styrene addition is 6.4 kcal/mol less favorable (via **mTS4a^+^**, see Supporting Information Figure S2 and Table S1) and is kinetically highly disfavored. The subsequent H_2_ hydrogenolysis of the Ca−C bond of **mB^+^** is −14.6 kcal/mol exergonic over a moderate barrier of 19.0 kcal/mol (via **mTS5^+^**) to form the hydrogenated product CH_3_CH_2_Ph along with regenerated catalyst **1^2+^** ⋅THF after **1 m^+^** dimerization and THF‐coordination, which is kinetically 1.6 kcal/mol more favorable than the preceding Markovnikov styrene addition. It is thus clear that the catalytic styrene hydrogenation via the terminal Ca−H bond of the solvent‐free cation monomer **1 m^+^** encounters only a moderate barrier of 20.6 kcal/mol, which is kinetically 3.7 kcal/mol more favorable than that via cooperative Ca−H−Ca bridges of the solvent‐free cation dimer **1^2+^** and reasonably accounts for the efficient styrene hydrogenation observed at room temperature.^[8]^

## Conclusion

Extensive dispersion‐corrected DFT calculations disclose a novel substrate‐dependent catalytic mechanism of 1‐alkene hydrogenation and H_2_ isotope exchange reactions with the same calcium hydride cation dimer catalyst **1^2+^** ⋅THF. It is shown that cooperative Ca−H−Ca bridges of solvent‐free dimer **1^2+^** are favored for H_2_ isotope exchange, competitive Ca−H−Ca bridges of **1^2+^** and the terminal Ca−H bond of cation monomer **1^+^** ⋅THF are involved in the anti‐Markovnikov addition of unactivated 1‐alkenes, while the terminal Ca−H bond of solvent‐free monomer **1^+^** is preferred for the Markovnikov addition of conjugation‐activated styrene as directed by strong cation‐π interactions. The novel mechanism can reasonably explain the known experimental observations and may be a useful guide for rational catalyst design.

## Computational Methods

All DFT calculations were performed with the TURBOMOLE 7.4 suite of programs.^[11]^ The structures were fully optimized at the TPSS−D3/def2‐TZVP+COSMO level in THF solution, which combines the TPSS meta‐GGA density functional^[12]^ with the BJ‐damped DFT−D3 dispersion correction^[13]^ and the def2‐TZVP basis set,^[14]^ using the Conductor‐like Screening Model (COSMO)^[15]^ for THF solvent (dielectric constant ϵ=7.58 and diameter R_solv_=3.18 Å). The density‐fitting RI−J approach^[16]^ was used to accelerate the calculations. The optimized structures were characterized by frequency analysis (no imaginary frequency for true minima and only one imaginary frequency for transition states) to provide thermal free‐energy corrections (at 298.15 K and 1 atm) according to the modified ideal gas‐rigid rotor‐harmonic oscillator model.^[17]^

More accurate solvation free energies in THF solution were computed with the COSMO‐RS model^[18]^ (parameter file: BP_TZVP_C30_1601.ctd) using the COSMOtherm package^[19]^ based on the TPSS−D3 optimized structures, corrected by +1.89 kcal/mol to account for the 1 mol/L reference concentration in solution. To check the effects of the chosen DFT functional on the reaction energies and barriers, single‐point calculations at both TPSS−D3^[12]^ and hybrid‐meta‐GGA PW6B95‐D3^[20]^ levels were performed using the larger def2‐QZVP^[14]^ basis set. Final reaction free energies (ΔG) were determined from the electronic single‐point energies plus TPSS−D3 thermal corrections and COSMO‐RS solvation free energies. As noted previously for similar hydrogenation reactions,[^10^, ^21^] the reaction energies from both DFT functionals are in very good mutual agreement of 0.0±1.4 kcal/mol (mean±standard deviation) though as expected 0.2±1.6 kcal/mol higher barriers were found at the PW6B95‐D3 level. In our discussion, the more reliable PW6B95‐D3+COSMO‐RS free energies (in kcal/mol, at 298.15 K and 1 mol/L concentration) were used unless specified otherwise. The applied DFT methods along with the large AO basis set provide usually accurate electronic energies leading to errors for chemical energies (including barriers) on the order of typically 1–2 kcal/mol. This has been thoroughly tested for the huge data base GMTKN55^[22]^ which is the common standard in the field of DFT benchmarking. For general recommendations on DFT based computational chemistry studies see Ref. [23].

## Conflict of interest

The authors declare no conflict of interest.

1

## Supporting information

As a service to our authors and readers, this journal provides supporting information supplied by the authors. Such materials are peer reviewed and may be re‐organized for online delivery, but are not copy‐edited or typeset. Technical support issues arising from supporting information (other than missing files) should be addressed to the authors.

Supporting Information

## Data Availability

The data that support the findings of this study are available in the supplementary material of this article.
