# Supplementary material for: Calcium Hydride Cation Dimer Catalyzed Hydrogenation of Unactivated 1‐Alkenes and H2 Isotope Exchange: Competitive Ca−H−Ca Bridges and Terminal Ca−H Bonds
Source: Chemistry. 2022 Nov 14;29(1):e202202602. doi: 10.1002/chem.202202602 (PMC10100058; doi:10.1002/chem.202202602)
Supplement: Supplementary file 1 — Supporting Information [file CHEM-29-0-s001.pdf]

Chemistry–A European Journal

Supporting Information

Calcium Hydride Cation Dimer Catalyzed Hydrogenation of Unactivated 1-Alkenes and H_2 Isotope Exchange: Competitive Ca–H–Ca Bridges and Terminal Ca–H Bonds

Zheng-Wang Qu,* Hui Zhu, and Stefan Grimme

Supporting Information

Table of Contents

Figure S1. DFT-computed free energy profile in THF solution for less favorable mechanisms of catalytic hydrogenation of unactivated CH_2=CHR (R= cyclohexenyl) S2
Figure S2. DFT-computed free energy profile in THF solution for less favorable mechanisms of catalytic hydrogenation of conjugation-activated CH_2=CHPh S3
Table S1. DFT-computed energies in THF solution. S4
Table S2. TPSS-D3/def2-TZVP + COSMO(THF) optimized Cartesian coordinates S10

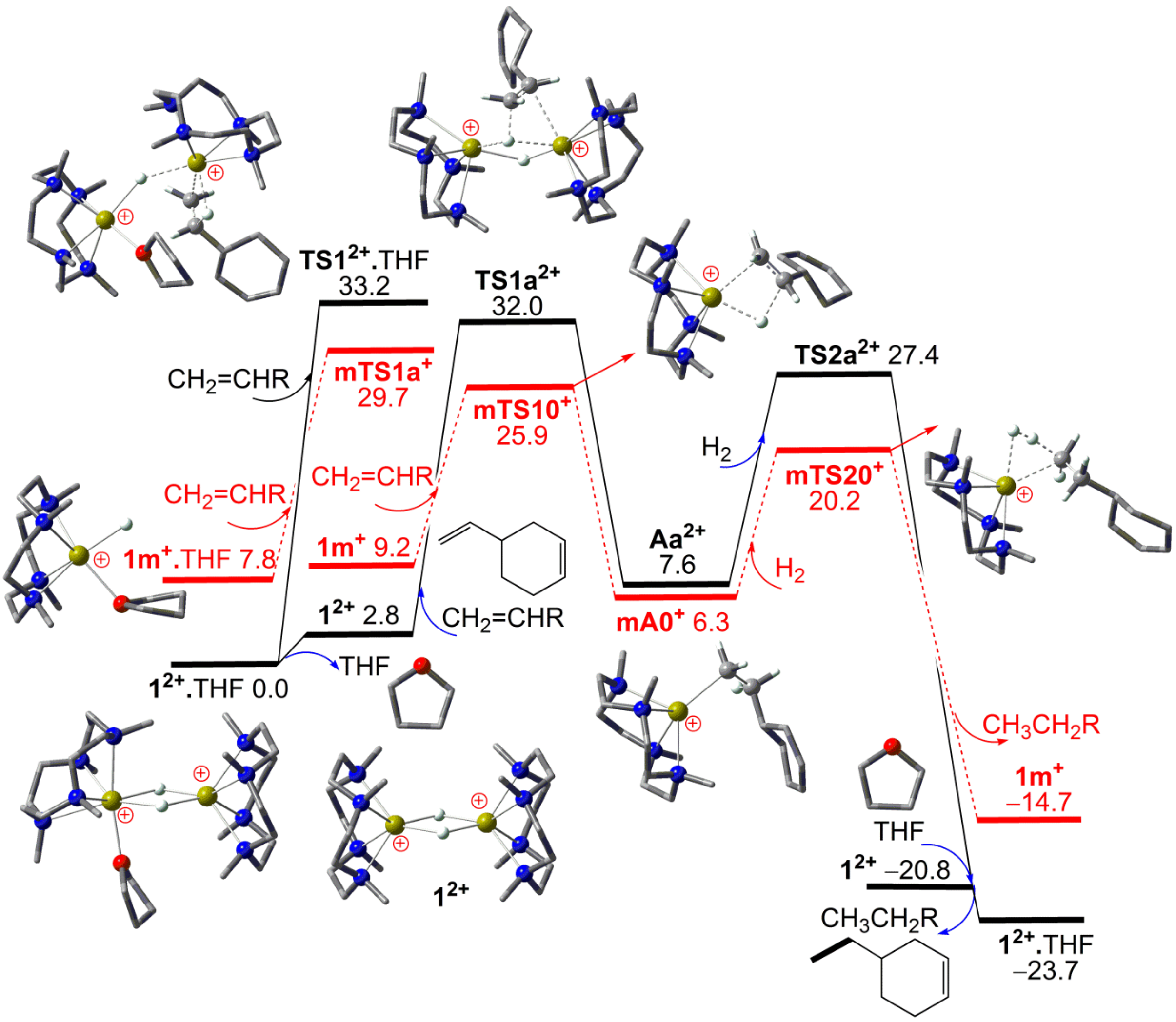

Figure S1. DFT-computed free energy profile (in kcal/mol, at 298 K and 1 M concentration in toluene solution) for *kineitically less favorable* mechanisms of catalytic hydrogenation of unactivated 1-akene CH_2=CHR (R = 3-cyclohexenyl) using the same calcium hydride cation dimer catalyst **1**$^{2+}$**.**THF in THF solution. Most H-atoms are omitted for clarity while crucial Ca, H, N and O atoms are high-lighted as yellow-green, white, blue and red balls in ball-and-stick models. Mechanisms involving terminal Ca-H bonds and cooperative Ca-H-Ca bridges are shown in red and in black lines, respectively. See also Table S1 for detailed DFT-computed energies.

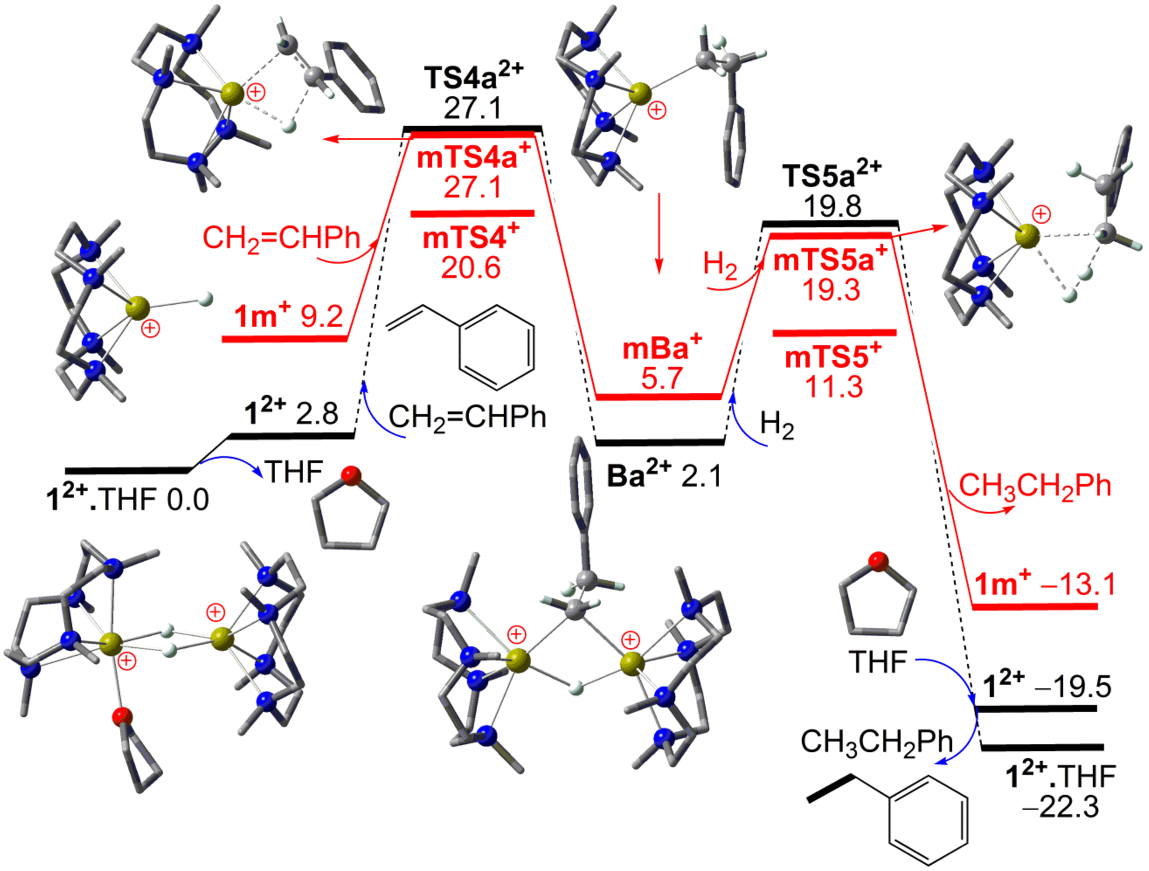

Figure S2. DFT-computed free energy profile (in kcal/mol, at 298 K and 1 M concentration in toluene solution) for *kineitically less favorable* mechanisms of catalytic hydrogenation of conjugation activated styrene CH_2=CHPh using the same calcium hydride cation dimer catalyst $\mathbf{1}^{2+}$**.**THF in THF solution. Most H-atoms are omitted for clarity while crucial Ca, H, N and O atoms are high-lighted as yellow-green, white, blue and red balls in ball-and-stick models. Mechanisms involving terminal Ca-H bonds and cooperative Ca-H-Ca bridges are shown in red and in black lines, respectively. See also Table S1 for detailed DFT-computed energies.

Table S1. DFT-computed energies in toluene solution. TPSS-D3/def2-TZVP + COSMO computed imaginary frequency (ImF), zero-point energies (ZPE), enthalpic (Hc) and Gibbs free-energy (Gc) corrections; the COSMO-RS computed solvation enthalpic (Hsol) and Gibbs free-energy (Gsol) corrections in THF solution; TPSS-D3/def2-QZVP and PW6B95-D3/def2-QZVP single-point energies (TPSS-D3 and PW6B95-D3 (E_P)); total PW6B95-D3 Gibbs free energies ($G_P = E_P + Gc + Gsol$) at 298 K, relative electronic energies (ΔE_T and ΔE_P) and final Gibbs free-energies (ΔG_T and ΔG_P) at the TPSS-D3 and PW6B95-D3 levels at 298 K. Each structure is labeled either by its molecular formula or a specific name in bold, with singly and doubly charged cations indicated by the + and 2+ superscripts, respectively. Dimeric and monomeric transition structures (with only one imaginary frequency) are indicated by the "**TS**" and "**mTS**" prefixes, respectively. See also main-text **Figures 1**, **2** and **3** and ESI **Figures S1** and S2 for structural labelling.

Reactions in THF (1M)	ImF cm^{-1}	ZPE kcal /mol	Hc kcal /mol	Gc kcal /mol	Hsol kcal /mol	Gsol kcal /mol	TPSS-D3 E_h	PW6B95-D3 E_h	G_P E_h	ΔE_T kcal /mol	ΔE_P kcal /mol	*ΔG_P kcal /mol*	ΔG_T kcal /mol	
THF	0	71.89	75.64	54.80	-6.76	-4.40	-232.60386	-232.84221	-232.75888					
One THF is -1.5 kcal/mol bound to cation monomer ***1m***$^+$*.*														
**1m**$^+$ + 2THF	0	400.19	421.41	337.47	-80.00	-64.36	-1836.91120	-1838.53617	-1838.09191	0.00	0.00	*0.00*	0.00	
**1m**$^+$ ·THF + THF	0	401.81	422.88	352.09	-67.84	-56.08	-1836.94546	-1838.57198	-1838.09423	-21.49	-22.47	*-1.46*	-0.48	
**1m**$^+$ ·(THF)$_2$	0	403.37	424.60	366.74	-58.21	-49.81	-1836.97125	-1838.60153	-1838.09345	-37.68	-41.02	*-0.96*	2.37	
.. THF is -2.8 kcal/mol bound to double Ca-H-Ca bridged dimer ***1***$^{2+}$														
**1**$^{2+}$ ·THF - THF	0	517.73	544.53	490.34	-131.38	-121.99	-2743.46265	-2745.76467	-2745.17767	0.00	0.00	*0.00*	0.00	
**1**$^{2+}$	0	516.49	542.91	476.67	-141.26	-127.96	-2743.43373	-2745.73194	-2745.17323	18.15	20.54	*2.79*	0.40	
**1**$^{2+}$ ·(THF)$_2$ - 2THF	0	519.47	546.37	505.57	-124.17	-117.03	-2743.46666	-2745.77044	-2745.15427	-2.52	-3.62	*14.68*	15.79	*Mono*
2***1m**$^+$ ·THF - 2THF	0	516.05	543.19	485.01	-108.66	-94.58	-2743.47549	-2745.77511	-2745.15292	-8.06	-6.55	*15.53*	14.02	*7.77*
2***1m**$^+$	0	512.81	540.25	455.75	-132.98	-111.14	-2743.40699	-2745.70348	-2745.14828	34.93	38.40	*18.44*	14.97	*9.22*
1m$^+$***.****THF catalyzed hydrogenation of unactivated CH_2=CHR (R = 3-Cyclohexenyl)*														
Selective anti-Markovnikov addition to terminal Ca-H bond over a barrier 23.8 kcal/mol														
**1m**$^+$.THF + CH_2=CHR	0	440.14	463.08	387.42	-70.21	-57.60	-1916.59104	-1918.31664	-1917.78502	0.00	0.00	*7.77*	0.00	
**mTS1**$^+$ (anti-Markovnikov)	376i	441.27	463.76	404.34	-59.81	-50.94	-1916.60023	-1918.32567	-1917.75949	-5.76	-5.67	*23.79*	15.93	
**mA**$^+$	0	444.12	467.13	405.63	-63.00	-53.50	-1916.62815	-1918.35521	-1917.79105	-23.29	-24.21	*3.99*	-2.86	
**mTS1a**$^+$ (Markovnikov)	567i	441.77	464.17	404.92	-59.69	-50.74	-1916.59300	-1918.31744	-1917.75000	-1.23	-0.51	*29.74*	21.25	
**mAa**$^+$	0	443.95	466.95	406.20	-61.74	-52.61	-1916.62110	-1918.34807	-1917.78158	-18.86	-19.72	*9.93*	3.02	
..followed by faster hydrogenolysis of Ca-C bond with H_2														
**1m**$^+$.THF + CH_2=CHR + H_2	0	446.47	471.48	386.54	-71.08	-56.60	-1917.77163	-1919.49258	-1918.95775	0.00	0.00	*7.77*	0.00	
**mTS2**$^+$	846i	453.60	476.55	415.95	-59.51	-50.42	-1917.80107	-1919.52240	-1918.93688	-18.47	-18.71	*20.87*	13.34	

**mTS2a**$^+$	800i	454.08	476.95	416.74	-58.59	-50.05	-1917.79870	-1919.52057	-1918.93320	-16.98	-17.57	*15.41*	15.99
**1m**$^+$.THF + CH_3CH_2R	0	454.63	477.88	401.62	-69.84	-57.29	-1917.82637	-1919.55067	-1918.99593	-34.35	-36.46	*-16.18*	-21.85
*..Alkene addition to THF-free monomer **1m**$^+$ encounters a 2.1 kcal/mol higher barrier of 25.9 kcal/mol*													
**1m**$^+$ + CH_2=CHR	0	366.62	385.97	317.99	-75.61	-61.49	-1683.95293	-1685.43861	-1685.02382	0.00	0.00	*9.22*	0.00
**1m**$^+$. CH_2=CHR	0	368.08	387.50	333.77	-65.16	-54.13	-1683.97109	-1685.45785	-1685.00922	-11.40	-12.08	*18.38*	9.84
**mTS10**$^+$ (anti-Markovnikov)	394i	367.72	386.32	334.53	-59.13	-50.11	-1683.96900	-1685.45345	-1684.99719	-10.08	-9.31	*25.93*	15.94
**mA0**$^+$	0	370.91	389.78	337.39	-63.01	-52.77	-1683.99830	-1685.48506	-1685.02848	-28.47	-29.15	*6.30*	-2.24
**mTS10a**$^+$ (Markovnikov)	475i	368.18	386.59	335.46	-58.46	-49.56	-1683.96929	-1685.45370	-1684.99507	-10.27	-9.47	*27.26*	17.24
**mA0a**$^+$	0	370.69	389.70	336.92	-61.92	-52.13	-1683.99348	-1685.47919	-1685.02234	-25.44	-25.46	*10.15*	0.95
*..THF may slightly stabilize **mA0**$^+$ by 2.3 kcal/mol*													
**mA0**$^+$ + THF	0	442.80	465.42	392.19	-69.77	-57.17	-1916.60216	-1918.32728	-1917.78736	0.00	0.00	*0.00*	0.00
**mA**$^+$	0	444.12	467.13	405.63	-63.00	-53.50	-1916.62815	-1918.35521	-1917.79105	-16.31	-17.53	*-2.32*	-1.10
..followed by faster hydrogenolysis of Ca-C bond with H_2													
**1m**$^+$ + CH_2=CHR + H_2	0	372.96	394.37	317.12	-76.48	-60.49	-1685.13352	-1686.61455	-1686.19655	0.00	0.00	*9.22*	0.00
**mA**$^+$.H_2	0	379.03	399.73	343.14	-64.79	-54.43	-1685.17692	-1686.65847	-1686.19536	-27.23	-27.56	*9.96*	1.07
**mTS20**$^+$ (anti-Markovnikov)	954i	379.72	398.84	345.90	-61.05	-51.30	-1685.17207	-1686.65155	-1686.17906	-24.19	-23.22	*20.19*	10.01
**mA0a**$^+$.H_2	0	379.93	399.88	345.70	-60.97	-51.53	-1685.17791	-1686.65962	-1686.18782	-27.85	-28.28	*14.70*	5.91
**mTS20a**$^+$ (Markovnikov)	824i	379.64	398.88	345.96	-59.79	-50.47	-1685.17412	-1686.65382	-1686.17992	-25.47	-24.64	*19.66*	9.60
**1m**$^+$ + CH_3CH_2R	0	381.11	400.77	332.19	-75.25	-61.17	-1685.18826	-1686.67265	-1686.23472	-34.35	-36.46	*-14.73*	-21.85
*Cation dimer **1**$^{2+}$.THF catalyzed hydrogenation of unactivated CH_2=CHR*													
Without THF elimination, 1-alkene addtion encounters a high barrier of 33.2 kcal/mol													
**1**$^{2+}$.THF + CH_2=CHR	0	699.84	736.01	635.25	-147.25	-132.30	-3288.31594	-3291.19375	-3290.38623	0.00	0.00	*0.00*	0.00
**TS1**$^{2+}$.THF	523i	700.01	736.17	650.85	-144.31	-129.71	-3288.29060	-3291.16690	-3290.33340	15.90	16.85	*33.15*	32.21
..After THF-elimination, anti-Markovnikov 1-alkene addtion encounters a reduced but still sizeable barrier of 23.8 kcal/mol													
**1**$^{2+}$ + CH_2=CHR	0	626.71	658.75	566.78	-150.38	-133.88	-3055.68316	-3058.31881	-3057.62290	0.00	0.00	*2.79*	0.00
**1**$^{2+}$. CH_2=CHR	0	627.23	659.85	581.24	-140.79	-127.78	-3055.70096	-3058.34056	-3057.61492	-11.17	-13.65	*7.80*	7.49
**TS1**$^{2+}$ (anti-Markovnikov)	616	627.38	659.21	582.29	-141.94	-128.24	-3055.67615	-3058.31607	-3057.58948	4.40	1.72	*23.76*	23.66
**A**$^{2+}$	0	630.81	662.91	585.25	-143.12	-129.19	-3055.71147	-3058.35134	-3057.62155	-17.76	-20.42	*3.64*	3.51
**TS1a**$^{2+}$ (Markovnikov)	727	628.22	659.97	583.51	-142.36	-128.58	-3055.66910	-3058.30542	-3057.57743	8.83	8.40	*31.33*	28.96
**Aa**$^{2+}$	0	630.66	662.68	585.61	-141.91	-128.32	-3055.70967	-3058.34877	-3057.61704	-16.64	-18.80	*6.47*	5.85

..followed by faster hydrogenolysis of Ca-C bond with H_2													
**1**$^{2+}$ + CH_2=CHR + H_2	0	633.04	667.15	565.91	-151.25	-132.88	-3056.86376	-3059.49475	-3058.79563	0.00	0.00	*2.79*	0.00
**TS2**$^{2+}$ (anti-Markovnikov)	774i	640.39	672.54	595.00	-142.91	-129.13	-3056.88241	-3059.51694	-3058.77153	-11.71	-13.92	*17.92*	17.34
**TS2a**$^{2+}$ (Markovnikov)	736i	640.24	672.35	595.35	-141.82	-128.35	-3056.87263	-3059.50496	-3058.75773	-5.57	-6.40	*26.57*	24.62
**1**$^{2+}$ + CH_3CH_2R	0	641.20	673.55	580.98	-150.02	-133.56	-3056.91850	-3059.55285	-3058.83381	-34.35	-36.46	*-21.16*	-21.85
*..THF-induced cleavage of **A**$^{2+}$ is 8.1 kcal/mol endergonic and thus is unlikely, though THF is -2.3 kcal/mol bound to **mA**$^{+}$*													
**mA**$^{+}$ + THF	0	442.80	465.42	392.19	-69.77	-57.17	-1916.60216	-1918.32728	-1917.78736	0.00	0.00	*0.00*	0.00
**mA**$^{+}$.THF	0	444.12	467.13	405.63	-63.00	-53.50	-1916.62815	-1918.35521	-1917.79105	-16.31	-17.53	*-2.32*	-1.10
**A**$^{2+}$ + 2THF	0	774.59	814.19	694.84	-156.63	-137.98	-3520.91918	-3524.03577	-3523.13932	0.00	0.00	*0.00*	0.00
**mA**$^{+}$.THF + **1m**$^{+}$.THF	0	774.04	814.37	702.93	-124.09	-105.19	-3520.96975	-3524.08498	-3523.12639	-31.74	-30.88	*8.11*	7.25
*Catalytic H_2 isotope exchange using cation dimer complex **1**$^{2+}$.THF*													
*THF-free dimer **1**$^{2+}$ is very reactive over a low barrier of 16.9 kcal/mol via cooperative Ca-H-Ca bridges.*													
**1**$^{2+}$ + H_2	0	522.82	551.31	475.79	-142.13	-126.96	-2744.61432	-2746.90788	-2746.34596	0.00	0.00	*2.79*	0.00
**TS3**$^{2+}$	913	525.51	552.08	485.42	-139.51	-127.24	-2744.60251	-2746.89729	-2746.32348	7.41	6.65	*16.90*	14.87
*..while cation monomer **1m**$^{+}$.THF is kinetically 3.9 kcal/mol less favorable via the terminal Ca-H bond*													
**1m**$^{+}$.THF + H_2	0	336.25	355.64	296.43	-61.96	-50.69	-1605.52219	-1606.90571	-1606.50808	0.00	0.00	*7.77*	0.00
**mTS3**$^{+}$	1158i	338.40	356.10	305.91	-56.54	-48.27	-1605.51888	-1606.90090	-1606.48731	2.08	3.02	*20.80*	12.09
*..direct **1**$^{2+}$.THF is kinetically 5.0 kcal/mol less favorable.*													
**1**$^{2+}$.THF + H_2	0	595.95	628.58	544.26	-139.00	-125.39	-2977.24710	-2979.78283	-2979.10929	0.00	0.00	*0.00*	0.00
**TS3a**$^{2+}$	814i	598.26	628.94	554.23	-138.05	-126.00	-2977.22492	-2979.75988	-2979.07444	13.91	14.40	*21.86*	21.38
*.THF-free cation monomer **1m**$^{+}$ is kinetically 7.8 kcal/mol less favorable for H2 isotope exchange*													
**1m**$^{+}$ + H_2	0	262.74	278.53	227.00	-67.36	-54.57	-1372.88409	-1374.02768	-1373.74687	0.00	0.00	*9.22*	0.00
**1m**$^{+}$.H_2	0	265.25	279.94	236.29	-65.25	-54.57	-1372.88741	-1374.03123	-1373.73863	-2.09	-2.23	*14.39*	5.32
**mTS3a**$^{+}$	1346i	264.39	278.49	235.65	-61.45	-51.64	-1372.87810	-1374.01854	-1373.72229	3.76	5.74	*24.65*	13.44
With conjugation-activated styrene CH_2=CHPh as substrate: Cation-Pi interaction directed Markovnikov alkene addition													
*Markovnikov addition of styrene to terminal Ca-H bond of THF-free **1m**$^{+}$ encounters a moderate barrier of 20.6 kcal/mol.*													
**1m**$^{+}$ + CH_2=CHPh	0	338.69	357.30	290.83	-76.32	-61.90	-1681.55738	-1683.04480	-1682.67396	0.00	0.00	*9.22*	0.00
**1m**$^{+}$.CH_2=CHPh	0	340.72	359.04	307.63	-61.88	-51.92	-1681.58382	-1683.07218	-1682.66166	-16.59	-17.18	*16.94*	8.30
**mTS4**$^{+}$ (Markovnikov)	142i	340.27	358.06	307.96	-60.13	-50.57	-1681.58334	-1683.06898	-1682.65579	-16.29	-15.17	*20.62*	10.29
**mB**$^{+}$	0	343.28	361.43	310.58	-60.95	-51.64	-1681.62586	-1683.11657	-1682.70091	-42.97	-45.03	*-7.69*	-14.85

**mTS4a**$^+$ (anti-Markovnikov)	572i	339.84	357.75	307.31	-59.87	-50.62	-1681.57270	-1683.05761	-1682.64555	-9.62	-8.04	*27.05*	16.25
**mBa**$^+$	0	342.95	361.16	309.98	-61.39	-51.63	-1681.60536	-1683.09424	-1682.67952	-30.11	-31.02	*5.73*	-2.57
..THF is not bound to benzyl calcium cation mB$^+$ due to strong cation-Pi interactions													
**mB**$^+$ + THF	0	415.17	437.07	365.37	-67.70	-56.04	-1914.22972	-1915.95878	-1915.45980	0.00	0.00	*0.00*	0.00
**mB**$^+$.THF	0	416.61	438.81	379.73	-65.20	-55.22	-1914.24434	-1915.97265	-1915.45249	-9.18	-8.70	*4.59*	4.12
..followed by faster hydrogenolysis of Ca-C bond with H_2													
**1m**$^+$ + CH_2=CHPh + H_2	0	345.02	365.70	289.95	-77.19	-60.90	-1682.73797	-1684.22074	-1683.84669	0.00	0.00	*9.22*	0.00
**mB**$^+$.H_2	0	352.13	371.49	318.56	-62.56	-52.72	-1682.80246	-1684.28846	-1683.86180	-40.46	-42.50	*-0.26*	-7.45
**mTS5**$^+$	1123i	352.19	370.59	319.24	-58.89	-50.03	-1682.79197	-1684.27537	-1683.84335	-33.88	-34.28	*11.32*	2.50
**mBa**$^+$.H_2	0	350.39	370.57	315.57	-61.30	-51.63	-1682.78834	-1684.27272	-1683.84909	-31.61	-32.61	*7.72*	-0.50
**mTS5a**$^+$	1012i	352.06	370.25	319.39	-59.33	-49.89	-1682.77931	-1684.26308	-1683.83059	-25.94	-26.57	*19.33*	10.73
**1m**$^+$ + CH_3CH_2Ph	0	353.06	372.07	304.70	-75.81	-61.60	-1682.78923	-1684.27562	-1683.88219	-32.16	-34.43	*-13.05*	-20.00
*.. THF-coordinated **1m**$^+$.THF is kinetically 5.1 kcal/mol less favorable*													
**mTS4**$^+$ + THF	1123	424.08	446.23	374.04	-65.64	-54.43	-1915.39582	-1917.11758	-1916.60223	0.00	0.00	*0.00*	0.00
**mTS4**$^+$.THF	813	425.53	447.94	388.62	-64.76	-54.75	-1915.40767	-1917.12911	-1916.59405	-7.43	-7.23	*5.14*	4.93
*.. Markovnikov addition of styrene to double Ca-H-Ca bridged cation dimer **1**$^{2+}$ is kinetically 3.7 kcal/mol less favorable*													
**1**$^{2+}$ + CH_2=CHPh	0	598.77	630.08	539.62	-151.09	-134.29	-3053.28761	-3055.92500	-3055.27305	0.00	0.00	*2.79*	0.00
**1**$^{2+}$.CH_2=CHPh	0	600.12	631.85	554.66	-141.13	-127.54	-3053.30821	-3055.94774	-3055.26406	-12.93	-14.27	*8.43*	6.98
**TS4**$^{2+}$ (Markovnikov)	541	600.33	631.26	556.29	-142.18	-128.35	-3053.28695	-3055.92372	-3055.23875	0.41	0.81	*24.31*	21.13
**B**$^{2+}$	0	602.96	634.42	558.54	-143.21	-129.30	-3053.32175	-3055.96418	-3055.27713	-21.42	-24.59	*0.23*	0.60
**TS4a**$^{2+}$ (anti-Markovnikov)	670	599.71	630.83	555.03	-141.85	-128.00	-3053.27894	-3055.91792	-3055.23438	5.44	4.45	*27.05*	25.26
**Ba**$^{2+}$	0	603.19	634.53	558.23	-143.63	-129.36	-3053.31813	-3055.96058	-3055.27414	-19.15	-22.33	*2.11*	2.49
*..with THF-induced **B**$^{2+}$ cleavage being slightly favored*													
**B**$^{2+}$ + THF	0	674.85	710.06	613.34	-149.96	-133.70	-3285.92561	-3288.80640	-3288.03601	0.00	0.00	*0.00*	0.00
**mB**$^+$ + **1m**$^+$.THF	0	673.20	708.66	607.87	-122.03	-103.32	-3285.96746	-3288.84633	-3288.03626	-26.26	-25.06	*-0.15*	-1.35
..followed by faster hydrogenolysis of Ca-C bond with H_2													
**1**$^{2+}$ + CH_2=CHPh + H_2	0	605.11	638.48	538.74	-151.96	-133.29	-3054.46820	-3057.10094	-3056.44578	0.00	0.00	*2.79*	0.00
**TS5**$^{2+}$	1065i	611.69	643.18	567.28	-143.18	-128.96	-3054.49149	-3057.12529	-3056.42377	-14.61	-15.28	*16.60*	14.48
**TS5a**$^{2+}$	766i	612.13	643.47	567.49	-142.95	-128.75	-3054.48540	-3057.12092	-3056.41873	-10.79	-12.53	*19.77*	18.72
**1**$^{2+}$ + CH_3CH_2Ph	0	613.14	644.85	553.49	-150.58	-133.98	-3054.51946	-3057.15582	-3056.48127	-32.16	-34.43	*-19.48*	-20.00

With small ethylene CH_2=CH_2 as substrate: smaller steric hindrance													
Alkene addition to termianl Ca-H bond of ***1m***$^+$*.THF is -0.4 kcal/mol exergonic over a moderate barrier of 19.2 kcal/mol*													
**1m**$^+$.THF + CH_2=CH_2	0	361.47	381.29	315.75	-64.07	-52.83	-1682.98321	-1684.45362	-1684.02861	0.00	0.00	*7.77*	0.00
**mTS6**$^+$	345i	362.89	381.92	329.21	-56.49	-48.34	-1682.99142	-1684.46101	-1684.01041	-5.15	-4.64	*19.19*	10.91
**mC**$^+$.THF	0	365.72	385.28	331.27	-57.63	-49.25	-1683.02229	-1684.49402	-1684.04158	-24.52	-25.35	*-0.37*	-7.30
**mC**$^+$ + THF	0	364.17	383.79	315.96	-68.52	-56.53	-1682.98978	-1684.45755	-1684.03811	-4.12	-2.47	*1.81*	-7.61
..followed by very faster hydrogenolysis of Ca-C bond with H_2													
**1m**$^+$.THF + CH_2=CH_2 + H_2	0	367.80	389.70	314.88	-64.94	-51.83	-1684.16381	-1685.62956	-1685.20134	0.00	0.00	*7.77*	0.00
**mTS7**$^+$	822i	375.01	394.63	340.67	-55.59	-47.59	-1684.19299	-1685.65917	-1685.18910	-18.31	-18.58	*15.45*	7.95
**1m**$^+$.THF + CH_3CH_3	0	376.00	396.12	329.92	-63.72	-52.54	-1684.22466	-1685.69333	-1685.24527	-38.19	-40.01	*-19.80*	-25.74
Double Ca-H-Ca bridged cation dimer ***1***$^{2+}$ *as catalyst:*													
Competitive CH_2=CH_2 addition to Ca-H-Ca bridges is kinetically 0.5 kcal/mol less favorable than to **1m**$^+$*.THF but can be favored upon heating*													
**1**$^{2+}$ + CH_2=CH_2	0	548.04	576.96	495.12	-144.25	-129.11	-2822.07534	-2824.45579	-2823.86649	0.00	0.00	*2.79*	0.00
**TS6**$^{2+}$	642i	549.93	577.99	508.57	-141.57	-127.99	-2822.06591	-2824.44909	-2823.83958	5.92	4.20	*19.68*	18.60
**C**$^{2+}$	0	552.25	580.82	510.21	-140.74	-127.72	-2822.10666	-2824.49217	-2823.87961	-19.65	-22.82	*-5.44*	-5.06
..followed by very fast hydrogenolysis of Ca-C bond with H_2													
**1**$^{2+}$ + CH_2=CH_2 + H_2	0	554.37	585.37	494.24	-145.12	-128.11	-2823.25593	-2825.63173	-2825.03922	0.00	0.00	*2.79*	0.00
**TS7**$^{2+}$	742i	561.47	589.69	519.94	-139.48	-126.87	-2823.27355	-2825.65265	-2825.02323	-11.06	-13.12	*12.83*	12.10
**1**$^{2+}$ + CH_3CH_3	0	562.57	591.79	509.29	-143.90	-128.82	-2823.31679	-2825.69550	-2825.08315	-38.19	-40.01	*-24.78*	-25.74
..the second CH_2=CH_2 addition to ***C***$^{2+}$ *is prevented by a sizable barrier of 27.7 kcal/mol thus is unlikely.*													
**C**$^{2+}$ + CH_2=CH_2	0	583.80	614.87	528.66	-143.72	-128.86	-2900.74827	-2903.21602	-2902.57288	0.00	0.00	*0.00*	0.00
**TS6a**$^{2+}$	639i	585.58	615.76	542.55	-143.29	-128.90	-2900.72463	-2903.19097	-2902.52876	14.84	15.72	*27.68*	26.80
1-hexene (CH_2=CHBu with Bu = Butyl) as a typical unactivated 1-alkene substrate													
Selective anti-Markovnikov alkene addition to terminal Ca-H bond of ***1m***$^+$*.THF encounters a sizable barrier of 24.6 kcal/mol*													
**1m**$^+$.THF + CH_2=CHBu	0	431.88	454.84	379.38	-68.08	-55.66	-1840.34748	-1841.98469	-1841.46279	0.00	0.00	*7.77*	0.00
**mTS8**$^+$ (anti-Markovnikov)	352i	432.91	455.45	395.72	-57.43	-48.85	-1840.35473	-1841.99181	-1841.43603	-4.55	-4.47	*24.56*	16.71
**mD**$^+$.THF	0	436.51	459.28	398.78	-60.26	-50.85	-1840.38406	-1842.02459	-1841.46711	-22.95	-25.04	*5.06*	-0.63
..followed by faster Ca-C bond hydrogenolysis with H_2													
**1m**$^+$.THF + CH_2=CHBu + H_2	0	438.21	463.24	378.50	-68.95	-54.66	-1841.52807	-1843.16063	-1842.63552	0.00	0.00	*7.77*	0.00
**mTS9**$^+$	803i	445.36	468.36	407.03	-58.23	-49.31	-1841.55554	-1843.18825	-1842.61519	-17.24	-17.33	*20.53*	12.85

**1m**$^+$.THF + CH_3CH_2Bu	0	446.35	469.66	393.50	-67.80	-55.36	-1841.58348	-1843.21923	-1842.67435	-34.77	-36.77	*-16.60*	-22.37

*Monomer **1m**$^+$ catalyzed hydrogenation of Cc = cyclohexene encounters a free energy barrier of 27.1 kcal/mol*

Cc + 0.5***1**$^{2+}$	0	348.49	365.78	311.03	-77.42	-68.19	-1606.52060	-1607.92229	-1607.53079	0.00	0.00	*1.40*	0.00
**1m**$^+$.Cc	0	348.72	366.20	316.77	-62.47	-52.14	-1606.52496	-1607.92752	-1607.50279	-2.74	-3.28	*18.97*	18.12
**cTS1**$^+$	522i	348.37	365.17	317.25	-56.91	-48.62	-1606.52104	-1607.92090	-1607.48981	-0.28	0.87	*27.12*	24.57
**cA**$^+$	0	351.63	368.70	320.05	-60.57	-51.02	-1606.54550	-1607.94657	-1607.51484	-15.63	-15.24	*11.41*	9.62
Cc + 0.5***1**$^{2+}$ + H_2	0	354.83	374.19	310.16	-78.29	-67.20	-1607.70119	-1609.09823	-1608.70352	0.00	0.00	*1.40*	0.00
**cA**$^+$.H_2	0	360.75	378.78	328.60	-59.64	-50.49	-1607.72860	-1609.12567	-1608.67947	-17.20	-17.22	*16.49*	15.11
**cTS2**$^+$	922i	360.67	377.88	329.15	-58.27	-49.19	-1607.72283	-1609.11783	-1608.66867	-13.58	-12.30	*23.26*	20.58
0.5***1**$^{2+}$ + CcH_2	0	363.37	380.80	326.45	-77.08	-67.87	-1607.75274	-1609.15287	-1608.73628	-32.35	-34.29	*-19.16*	-18.62

Temperature effects upon heating at 333 K (as compared to reaction at 298 K using unactivated CH_2=CHR (R = 3-Cyclohexenyl) as substrate):

*Dimeric mechanism via **TS1**$^{2+}$ becomes kinetically 1.4 kcal/mol more favorable than monomeric mechanism via **mTS1**$^+$*

												333 K		*298 K*
**1m**$^+$.THF + CH_2=CHR	0	440.14	468.16	378.58	-68.56	-56.22	-1916.59104	-1918.31664	-1917.79753	0.00	0.00	*7.77*	0.00	*7.77*
**mTS1**$^+$ (anti-Markovnikov)	376i	441.27	468.88	397.47	-58.71	-49.96	-1916.60023	-1918.32567	-1917.76918	-5.76	-5.67	*25.55*	17.70	*23.79*
**1**$^{2+}$.THF + CH_2=CHR - THF	0	627.95	667.77	571.24	-138.73	-126.53	-3055.71209	-3058.35154	-3057.64016	0.00	0.00	*0.00*	0.00	*0.00*
**TS1**$^{2+}$ (anti-Markovnikov)	616i	627.38	666.56	573.39	-139.77	-126.75	-3055.67615	-3058.31607	-3057.60162	22.55	22.25	*24.19*	24.48	*23.76*

*..The barrier for monomeric mechanism via THF-free **1m**$^+$ and **mTS4**$^+$ is insensitive to temperature.*

**1m**$^+$ + CH_2=CHPh	0	338.69	361.39	283.00	-74.04	-60.34	-1681.55738	-1683.04480	-1682.68458	0.00	0.00	*7.82*	0.00	*9.22*
**mTS4**$^+$	142i	340.27	362.17	302.10	-58.71	-49.53	-1681.58334	-1683.06898	-1682.66378	-16.29	-15.17	*20.87*	11.93	*20.62*

*..mainly due to entropy-favored THF elimination from dimer **1**$^{2+}$.THF and monomer **1m**$^+$.THF*

**1**$^{2+}$ + THF	0	588.38	625.34	521.29	-145.60	-130.65	-2976.03758	-2978.57415	-2977.94623	0.00	0.00	*0.00*	0.00	*0.00*
**1**$^{2+}$.THF	0	589.62	627.08	536.50	-136.43	-125.10	-2976.06651	-2978.60688	-2977.94858	-18.15	-20.54	*-1.47*	0.92	*-2.79*
**1m**$^+$ + THF	0	328.29	349.53	275.26	-71.11	-58.53	-1604.30735	-1605.69395	-1605.34318	0.00	0.00	*0.00*	0.00	*0.00*
**1m**$^+$.THF	0	329.92	351.09	291.50	-59.70	-50.66	-1604.34160	-1605.72977	-1605.34327	-21.49	-22.47	*-0.05*	0.93	*-1.46*

..with dimer-to-monomer equlibrium being insensitive to small temperature changes

**1**$^{2+}$.THF + THF	0	661.51	703.44	588.84	-142.99	-129.23	-3208.67036	-3211.44910	-3210.71127	0.00	0.00	*0.00*	0.00	*0.00*
**1m**$^+$.THF + **1m**$^+$.THF	0	659.83	702.19	583.00	-119.40	-101.32	-3208.68320	-3211.45953	-3210.68653	-8.06	-6.55	*15.52*	14.01	*15.53*

Table S2. TPSS-D3/def2-TZVP + COSMO optimized Cartesian coordinates (in Å) in THF solution. Each structure is labeled by the specific name (See also **Table S1**, main-text **Figures 1**, **2** and **3** and ESI **Figures S1** and S**2**), followed by the number of atoms, the total energy, and the detailed atomic coordinates (in double-column text list).

1^{2+}**.**CH_2=CHPh : loose complex of CH_2=CHPh
108
Energy = -3053.310725490

Ca	0.2198019	0.0852148	1.6472619
Ca	-0.4305862	1.0903093	-1.7038933
H	0.7345277	1.5797365	0.0933439
N	1.5329788	-1.2040611	3.4912136
N	-0.6052636	-0.4176737	-3.7457618
N	-1.7018325	1.4058483	2.5924007
N	-0.5794410	3.5692399	-2.2352541
N	1.1833305	1.7942481	3.3167750
N	1.5402739	1.6023796	-3.2362167
N	-1.3616828	-1.5786599	2.7567245
N	-2.7081898	1.5440104	-2.7610760
C	-1.1737885	2.6218319	3.2622536
C	0.7227116	3.9409552	-2.8499752
C	0.7143147	-2.3805010	3.8905400
C	-2.0236696	-0.3979866	-4.1914951
C	0.0509428	2.3262354	4.1174775
C	1.2146556	2.9115248	-3.8609075
C	-0.7672279	-2.0517664	4.0312716
C	-2.6312861	0.9988216	-4.1426400
C	2.8755227	-1.6825455	3.0890129
C	-0.2226734	-1.8082407	-3.4120349
C	-2.5839148	1.8065999	1.4796082
C	-0.7751079	4.3794519	-1.0122749
C	1.6750599	-0.2657460	4.6360929
C	0.2833384	0.1148208	-4.8109356
C	-2.4410718	0.5400104	3.5449855
C	-1.6994668	3.7968093	-3.1841812
C	2.1605256	1.1100671	4.2018200
C	1.6438819	0.5408323	-4.2722607
C	-2.6439719	-0.8700284	3.0009447
C	-2.9476038	3.0115633	-2.7963523
C	1.8643096	2.9033861	2.6107241
C	2.8285151	1.7056659	-2.5122320
C	-1.6113361	-2.7286734	1.8590677
C	-3.8111828	0.8827226	-2.0269583
H	-1.9486684	3.0953989	3.8839303
H	0.6511962	4.9230846	-3.3412492
H	1.0807723	-2.8042333	4.8381996
H	-2.1154014	-0.7952820	-5.2135169
H	-0.9133875	3.3336339	2.4736088
H	1.4448339	4.0409208	-2.0350980
H	0.8586226	-3.1460553	3.1217895
H	-2.5795203	-1.0736926	-3.5359802
H	2.7695936	-2.4127100	2.2862877
H	-0.8491616	-2.1604018	-2.5887638
H	-2.0000412	2.4084276	0.7777160
H	-0.0133126	4.1010425	-0.2799035

H	0.7049079	-0.1775031	5.1305427
H	-0.2186844	0.9649389	-5.2791221
H	-1.8758310	0.5046061	4.4798058
H	-1.3665700	3.5032702	-4.1825793
H	-0.2004770	1.5916394	4.8865215
H	0.4520000	2.7461398	-4.6261838
H	-0.9107744	-1.2743268	4.7856900
H	-2.0342857	1.6860782	-4.7472335
H	3.3959705	-2.1538941	3.9362228
H	-0.3499950	-2.4754800	-4.2772949
H	-3.4410916	2.3997422	1.8326277
H	-0.7044647	5.4552995	-1.2303312
H	2.3730725	-0.6808350	5.3790748
H	0.4291523	-0.6388555	-5.5994924
H	-3.4217609	0.9800342	3.7827111
H	-1.9492704	4.8671346	-3.2371462
H	0.3531857	3.2444131	4.6442105
H	2.0999273	3.3125515	-4.3778436
H	-1.2981936	-2.9447939	4.3950443
H	-3.6354228	0.9699986	-4.5925874
H	3.4814058	-0.8489239	2.7272215
H	0.8213614	-1.8426918	-3.0943403
H	-2.9446166	0.9186128	0.9596470
H	-1.7625103	4.1819108	-0.5890191
H	1.1580203	3.4125526	1.9542564
H	2.7280302	2.3872639	-1.6659646
H	-0.6852161	-3.2871293	1.7103018
H	-3.5910802	-0.1780990	-1.8913340
H	3.0986258	1.0222612	3.6465691
H	2.1463628	-0.3126771	-3.8078499
H	-3.1727585	-0.8311997	2.0446965
H	-3.2805661	3.3079115	-1.7972713
H	2.3733191	1.7187394	5.0939227
H	2.2773653	0.8693131	-5.1103079
H	-3.2782951	-1.4387967	3.6978071
H	-3.7637379	3.2599149	-3.4915266
H	2.6642206	2.4964740	1.9885125
H	3.1018381	0.7206890	-2.1295877
H	-1.9484796	-2.3503291	0.8912945
H	-3.9133022	1.3429218	-1.0425496
H	2.2868300	3.6257078	3.3261299
H	3.6292976	2.0612745	-3.1777946
H	-2.3643053	-3.4093165	2.2842549
H	-4.7657691	0.9858934	-2.5638080
C	2.3459370	-1.2998580	-0.2808251
C	1.5208673	-2.3615744	-0.2913074
H	1.9638012	-0.2946626	-0.4691306
H	3.4137410	-1.3765229	-0.0967676
H	0.4645000	-2.1714965	-0.4911550
H	-0.9563110	-0.4263989	-0.2059567

C	1.8713142	-3.7627829	-0.0473778
C	3.1716304	-4.1797011	0.2944632
C	0.8616657	-4.7364764	-0.1483347
C	3.4440937	-5.5216805	0.5373169
H	3.9749013	-3.4519451	0.3668494
C	1.1343901	-6.0815159	0.0933851
H	-0.1436215	-4.4297730	-0.4266082
C	2.4266203	-6.4774672	0.4412660
H	4.4521641	-5.8276162	0.8013203
H	0.3415443	-6.8188447	0.0092796
H	2.6437643	-7.5240062	0.6331045

1^{2+}.CH_2=CHR : loose complex of CH_2=CHR

112

Energy = -3055.701858657

Ca	-0.1058196	0.5488432	1.8824261
Ca	0.2100969	0.4579146	-1.6668165
H	1.2652907	1.0224755	0.2291924
N	1.6246532	-0.5884195	3.3598261
N	0.5376489	-0.5247041	-4.1043670
N	-2.0198142	1.6276727	3.2030577
N	-0.5405418	2.8568331	-1.7429033
N	0.8760344	2.3725102	3.3820568
N	2.0386026	1.8168164	-2.8787084
N	-1.2619680	-1.3212087	3.1754149
N	-2.0280676	0.5301543	-2.9244699
C	-1.5294194	2.8852873	3.8283530
C	0.6286487	3.7126852	-2.0656513
C	1.0742982	-1.8728396	3.8672952
C	-0.8128730	-0.7660458	-4.6805805
C	-0.1289820	2.7418626	4.4126563
C	1.4599422	3.1457037	-3.2074875
C	-0.3847265	-1.7566473	4.2920671
C	-1.8035198	0.3498290	-4.3797585
C	2.8937101	-0.8529368	2.6432351
C	1.3140205	-1.7795473	-4.2304998
C	-3.2118084	1.9261426	2.3759858
C	-1.0475450	3.2106836	-0.4054490
C	1.8731587	0.3513246	4.4845794
C	1.2349470	0.5436328	-4.8701636
C	-2.3842427	0.6370936	4.2510310
C	-1.6204766	2.9948210	-2.7500122
C	2.0867829	1.7856317	4.0165681
C	2.4347174	1.1090756	-4.1227831
C	-2.5413140	-0.7729301	3.6966902
C	-2.6337198	1.8593804	-2.6547219
C	1.2809681	3.5746040	2.6181430
C	3.2300813	1.9947980	-2.0172566
C	-1.5562648	-2.4640776	2.2837886
C	-2.9436178	-0.5199825	-2.4202263
H	-2.2175218	3.2250089	4.6173340
H	0.3101689	4.7357890	-2.3167126
H	1.6694735	-2.2489733	4.7131179
H	-0.7465997	-0.9036706	-5.7710607
H	-1.5330781	3.6542028	3.0500345
H	1.2369703	3.7725759	-1.1585754
H	1.1738238	-2.6052405	3.0601081
H	-1.1750976	-1.7114891	-4.2650267
H	2.7135815	-1.5846886	1.8541730
H	0.8297449	-2.5669204	-3.6512921
H	-2.9730504	2.7321277	1.6803333
H	-0.2296710	3.0823439	0.3082303
H	1.0194834	0.3042512	5.1652709
H	0.5178306	1.3410756	-5.0776518
H	-1.6066378	0.6492613	5.0187419
H	-1.1619471	3.0081913	-3.7419358
H	-0.1222484	1.9721587	5.1878662
H	0.8412607	3.0405401	-4.1021569
H	-0.4818610	-1.0326275	5.1046715
H	-1.4409179	1.2951092	-4.7905708
H	3.6610161	-1.2444159	3.3276055
H	1.3776271	-2.0974488	-5.2818810
H	-4.0602421	2.2434402	3.0000438
H	-1.4063198	4.2505151	-0.3639100
H	2.7547640	0.0269943	5.0582946
H	1.5639553	0.1535174	-5.8454252
H	-3.3214503	0.9384425	4.7433053
H	-2.1417266	3.9566935	-2.6272805
H	0.1534962	3.6851067	4.9038277
H	2.2612850	3.8570448	-3.4594162
H	-0.7224520	-2.7248363	4.6912940
H	-2.7533196	0.1290505	-4.8906084
H	3.2598501	0.0647178	2.1793640
H	2.3244815	-1.6374914	-3.8463070
H	-3.5023501	1.0440357	1.8012551
H	-1.8624483	2.5370359	-0.1366731
H	0.3974589	4.0792870	2.2215708
H	2.9361293	2.4527833	-1.0720310
H	-0.6212671	-2.9065385	1.9344525
H	-2.5468972	-1.5075341	-2.6656957
H	2.8895472	1.8239594	3.2754535
H	3.1154627	0.3025034	-3.8360555
H	-3.2568623	-0.7766144	2.8703125
H	-3.0587904	1.8176467	-1.6480962
H	2.4091394	2.3988623	4.8716016
H	2.9962089	1.7783474	-4.7925046
H	-2.9528499	-1.4260994	4.4807110
H	-3.4661947	2.0563290	-3.3474086
H	1.9203526	3.2695573	1.7854201
H	3.6643633	1.0167590	-1.7992969
H	-2.1133940	-2.1046983	1.4158264
H	-3.0057926	-0.4359280	-1.3332945
H	1.8249632	4.2863051	3.2565729
H	3.9881391	2.6188272	-2.5148732
H	-2.1341784	-3.2413693	2.8053547
H	-3.9421799	-0.4243952	-2.8726044
C	2.4819725	-1.7136936	-0.9374519
C	1.3765966	-2.3531037	-0.5380803
H	2.7518870	-0.7512187	-0.5067125
H	3.1543498	-2.1457598	-1.6743454

H	0.7354660	-1.8760253	0.2109014
H	-1.1399443	-0.0541182	0.0406817
C	0.9087445	-3.6970867	-1.0086847
C	1.1062767	-4.7614762	0.0945592
C	-0.5739725	-3.6610635	-1.4269823
H	1.5127336	-4.0005230	-1.8739899
C	0.3827825	-6.0447177	-0.2165637
H	0.7616565	-4.3671530	1.0638039
H	2.1781430	-4.9561978	0.2210778
C	-1.0135651	-5.0048731	-2.0161826
H	-1.1853130	-3.4212072	-0.5488285
H	-0.7423747	-2.8558534	-2.1526914
C	-0.5459828	-6.1600606	-1.1713205
H	0.6596959	-6.9153593	0.3756185
H	-2.1065148	-5.0338160	-2.1082282
H	-0.6247634	-5.1151330	-3.0398890
H	-1.0047919	-7.1290551	-1.3599942

$1H^+$: triple Ca-H-Ca bdridged dimer cation
93
Energy = -2744.156434671

Ca	16.7940387	10.7880588	14.6722310
H	19.0335252	10.6468873	14.7738987
N	17.4022970	13.1771653	13.8447062
N	15.0329160	11.6228438	12.9006064
N	14.3898323	10.7983642	15.7076893
N	16.7997750	12.3657136	16.6886817
C	16.2540811	13.7888833	13.1436135
H	16.5708788	14.6792209	12.5750209
H	15.5377298	14.1332399	13.8943562
C	15.5780002	12.8051910	12.1934386
H	14.7877010	13.3273941	11.6294530
H	16.3021568	12.4397267	11.4600220
C	13.7714007	11.9737434	13.5903087
H	12.9497158	12.0793114	12.8620259
H	13.9037804	12.9521529	14.0593243
C	13.3721101	10.9462262	14.6425950
H	12.3924206	11.2262706	15.0643728
H	13.2468438	9.9652490	14.1758485
C	14.3313349	11.9395260	16.6500994
H	13.4617598	11.8376862	17.3211494
H	14.1739439	12.8526693	16.0693438
C	15.5943529	12.0736208	17.4953274
H	15.4314629	12.8495265	18.2625437
H	15.7887080	11.1327627	18.0172608
C	16.8224012	13.7642102	16.2124894
H	17.1083014	14.4503815	17.0276315
H	15.8087374	14.0413448	15.9099548
C	17.7861244	13.9573675	15.0439102
H	17.8600715	15.0323110	14.8085449
H	18.7845997	13.6173198	15.3316399
C	18.5658745	13.0713851	12.9409329
H	18.2965450	12.4939307	12.0530001
H	19.3559614	12.5320284	13.4687127
H	18.9169410	14.0660841	12.6198940
C	14.8071378	10.5288180	11.9342482
H	14.4233902	9.6477945	12.4520611
H	15.7607235	10.2534051	11.4792771
H	14.0902928	10.8258192	11.1507617
C	14.1684807	9.5261265	16.4257212
H	14.9625313	9.3760136	17.1578622
H	14.2266939	8.7025712	15.7095509
H	13.1819193	9.5128134	16.9191576
C	18.0073692	12.0621249	17.4827425
H	18.8971568	12.2285025	16.8738697
H	17.9811201	11.0011490	17.7428470
H	18.0544011	12.6848863	18.3924356
Ca	18.8383389	8.4199156	15.0093084
H	17.2019494	9.2216048	16.2666726
N	19.2449342	6.4091681	16.6625592
N	21.2181722	8.5869854	16.0865490
N	20.5727282	8.3560979	13.1272560
N	18.5966826	6.1603596	13.7574428
C	20.6891902	6.3100265	16.9703595
H	20.8538981	5.6553868	17.8426164
H	21.1838814	5.8322477	16.1206347
C	21.3256189	7.6678502	17.2423117
H	22.3778882	7.5190757	17.5365048
H	20.8259683	8.1501687	18.0870986
C	22.1924431	8.2189769	15.0334562
H	23.2090420	8.5303557	15.3273843
H	22.2096652	7.1286600	14.9515704
C	21.8597338	8.8349532	13.6781394
H	22.6904528	8.6351495	12.9802411
H	21.7724389	9.9202564	13.7791553
C	20.6715571	6.9756436	12.6090887
H	21.1794626	6.9616573	11.6299523
H	21.2951549	6.3945676	13.2942428
C	19.3015733	6.3167211	12.4642039
H	19.4189215	5.3436305	11.9585173
H	18.6608280	6.9380871	11.8327105
C	19.1694726	5.0598994	14.5600679
H	18.8533318	4.0798408	14.1651118
H	20.2583769	5.0994258	14.4692538
C	18.7673098	5.1577261	16.0287791
H	19.1421942	4.2730381	16.5693313
H	17.6774237	5.1444447	16.1170498
C	18.4705343	6.6673086	17.8935416
H	18.8025528	7.6000574	18.3535995
H	17.4167341	6.7852843	17.6331146
H	18.5867521	5.8473023	18.6214930
C	21.4480048	9.9748951	16.5371975
H	21.2670527	10.6627899	15.7104953
H	20.7282251	10.2128858	17.3240145
H	22.4709569	10.1041530	16.9293334
C	20.1180515	9.2818540	12.0700862
H	19.1421814	8.9650582	11.6991976
H	19.9914942	10.2694787	12.5184718
H	20.8416904	9.3236353	11.2380670
C	17.1597753	5.9274631	13.5041170

H	16.6300382	5.8069212	14.4523124
H	16.7562216	6.8107892	13.0032115
H	17.0018755	5.0217386	12.8953240
H	17.2611655	8.9845261	13.4726241

1m$^+$.CH_2=CHPh : loose complex of styrene
62
Energy = -1681.560390993

Ca	22.0095397	-0.3910110	13.8205725
H	20.5034498	-0.2789278	12.3438212
N	21.6567715	-2.8987696	13.8464892
N	23.6001552	-1.3074003	12.1592516
N	24.4119703	0.3462361	14.5016159
N	22.4166034	-1.2279139	16.1862600
C	22.8120666	-3.4822965	13.1153224
H	22.5983471	-4.5262787	12.8399200
H	23.6701229	-3.5045747	13.7928212
C	23.1573893	-2.6943523	11.8565412
H	23.9310315	-3.2331445	11.2891179
H	22.2769648	-2.6135574	11.2141013
C	24.9857924	-1.2896909	12.6857430
H	25.7098604	-1.4695176	11.8764261
H	25.0919102	-2.1166189	13.3929427
C	25.3232142	0.0309783	13.3667784
H	26.3695000	0.0060204	13.7069831
H	25.2410195	0.8536916	12.6515587
C	24.7546726	-0.4912748	15.6830763
H	25.6654388	-0.1060959	16.1657887
H	24.9890204	-1.4989694	15.3318699
C	23.6293669	-0.5444203	16.7090096
H	23.9920319	-1.0433301	17.6202679
H	23.3326826	0.4684976	16.9973866
C	22.6365186	-2.6976467	16.1307752
H	22.5965927	-3.1248922	17.1440569
H	23.6446216	-2.8784172	15.7502517
C	21.6158186	-3.3973028	15.2448215
H	21.7849885	-4.4840072	15.2818836
H	20.6050765	-3.2205844	15.6227155
C	20.3926393	-3.2643611	13.1642550
H	20.3958112	-2.8744305	12.1467738
H	19.5546507	-2.8057689	13.6941159
H	20.2599858	-4.3569842	13.1477681
C	23.4932756	-0.4802894	10.9342043
H	23.8168991	0.5423603	11.1439467
H	22.4463268	-0.4549152	10.6233302
H	24.1182359	-0.8854956	10.1249641
C	24.5893864	1.7738325	14.8549242
H	23.9405997	2.0354723	15.6930255
H	24.3243290	2.3944758	13.9974416
H	25.6313168	1.9811201	15.1406666
C	21.2677076	-0.9241476	17.0698176
H	20.3642723	-1.4041563	16.6888842
H	21.1029855	0.1548853	17.0904958
H	21.4491015	-1.2797834	18.0948263
C	21.4421705	2.6099427	12.9027724
C	21.3910616	2.8301551	14.2305419
H	22.2295691	3.0577256	12.3030100
H	20.7126808	1.9953162	12.3798945
H	22.1476984	3.4715475	14.6802861
C	20.3946867	2.3096484	15.1762127
C	19.3797433	1.4058221	14.7955131
C	20.4460748	2.7345882	16.5177812
C	18.4524296	0.9520017	15.7313813
H	19.3243666	1.0541536	13.7657515
C	19.5042078	2.2929210	17.4451474
H	21.2242689	3.4298014	16.8234110
C	18.5034926	1.3995931	17.0547778
H	17.6780578	0.2551125	15.4235492
H	19.5525290	2.6426647	18.4721319
H	17.7695221	1.0533654	17.7761762

1m$^+$.CH_2=CHR : loose complex of CH_2=CHR
66
Energy = -1683.948294174

Ca	22.5162770	-0.1714748	14.0192072
H	21.0674527	0.7806027	12.8400464
N	21.5577974	-2.4969466	14.0495379
N	23.4416766	-1.3276627	12.0020720
N	25.0663518	-0.0697545	14.1675348
N	23.1766563	-1.2516276	16.2529690
C	22.3583638	-3.3034408	13.0889516
H	21.8360439	-4.2447994	12.8614424
H	23.3003318	-3.5739424	13.5730322
C	22.6338621	-2.5571335	11.7891406
H	23.1345950	-3.2348510	11.0814253
H	21.6930542	-2.2464667	11.3276854
C	24.8673753	-1.6603001	12.2387585
H	25.3578191	-1.9529079	11.2978306
H	24.9112217	-2.5295008	12.9003734
C	25.6287213	-0.4927172	12.8541111
H	26.6898278	-0.7631790	12.9600542
H	25.5858620	0.3753800	12.1907754
C	25.4624141	-1.0367838	15.2272326
H	26.5182593	-0.8868434	15.4980989
H	25.3866039	-2.0447316	14.8121263
C	24.6099004	-0.9228470	16.4849394
H	25.0336111	-1.5749066	17.2637832
H	24.6452599	0.0993920	16.8726852
C	22.9985522	-2.7184732	16.0751797
H	23.0698348	-3.2292165	17.0470209
H	23.8219961	-3.0919981	15.4619715
C	21.6667167	-3.0629706	15.4197920
H	21.5392437	-4.1555153	15.4052299
H	20.8402886	-2.6537619	16.0078914
C	20.1372864	-2.4788506	13.6258680
H	20.0437156	-1.9803568	12.6598423
H	19.5512021	-1.9168159	14.3563093
H	19.7329432	-3.4990895	13.5536386
C	23.3068922	-0.4472526	10.8170548
H	23.8967978	0.4612274	10.9573734

H	22.2594224	-0.1598133	10.7107823
H	23.6560295	-0.9568044	9.9066764
C	25.5995125	1.2738912	14.4946574
H	25.2580156	1.5804452	15.4845471
H	25.2415912	1.9969017	13.7571652
H	26.6991831	1.2741333	14.4895740
C	22.3962374	-0.8022028	17.4291360
H	21.3369981	-1.0212487	17.2830256
H	22.5178510	0.2753059	17.5513830
H	22.7359327	-1.3075837	18.3451493
C	22.3640455	2.6922883	15.2778129
C	21.2837006	2.3903839	16.0118631
H	23.3338211	2.8557016	15.7393076
H	22.2822496	2.8515324	14.2024893
H	21.3942451	2.2820976	17.0929989
C	19.8916003	2.2325188	15.4813653
C	19.3068107	0.8599640	15.8658919
C	18.9672842	3.3505106	16.0103174
H	19.9285169	2.2847979	14.3857218
C	17.8287358	0.7692879	15.5960191
H	19.5114413	0.6422975	16.9250669
H	19.8224814	0.0802883	15.2841586
C	17.5787160	3.2492125	15.3662269
H	18.8767376	3.2543748	17.1005912
H	19.4113569	4.3300715	15.8044390
C	17.0604023	1.8361844	15.3545896
H	17.3874950	-0.2265555	15.5946929
H	16.8713241	3.8950780	15.9020167
H	17.6126541	3.6339405	14.3358891
H	16.0017926	1.6966013	15.1402877

1m^{+}.H_2 : loose cmplex of H_2
48
Energy = -1372.890596600

Ca	22.3761521	-0.6563793	14.6038477
H	20.6326511	0.2845253	15.2707450
N	21.8731724	-2.9934200	13.9240765
N	23.3330172	-0.9343610	12.2861849
N	24.8094426	-0.0451593	14.7764702
N	23.3386730	-2.1138976	16.3813144
C	22.7645460	-3.3304197	12.7836395
H	22.4315447	-4.2611189	12.3003945
H	23.7646068	-3.5216209	13.1816241
C	22.8190345	-2.2216529	11.7385822
H	23.4374535	-2.5539925	10.8918734
H	21.8182313	-2.0267170	11.3436248
C	24.8113354	-0.9947059	12.4540645
H	25.3073109	-0.8962956	11.4772711
H	25.0688446	-1.9848934	12.8384516
C	25.3416944	0.0850081	13.3918690
H	26.4412392	0.0514505	13.3939317
H	25.0541661	1.0752579	13.0270737
C	25.4726025	-1.1731441	15.4869209
H	26.4935923	-0.8874548	15.7799873
H	25.5657984	-2.0078341	14.7876056

C	24.6983782	-1.6160043	16.7232381
H	25.2742444	-2.3861675	17.2564980
H	24.5743762	-0.7766201	17.4127227
C	23.4080800	-3.4958606	15.8363741
H	23.5826889	-4.2171862	16.6483857
H	24.2731316	-3.5537906	15.1703329
C	22.1403464	-3.8868813	15.0842481
H	22.2193370	-4.9344707	14.7591229
H	21.2741059	-3.8228445	15.7480290
C	20.4500013	-3.1108802	13.5258397
H	20.2412429	-2.4489129	12.6825789
H	19.8167248	-2.8118592	14.3645429
H	20.2023897	-4.1414395	13.2340180
C	22.9753626	0.1561531	11.3466609
H	23.3924838	1.1027601	11.6945007
H	21.8877011	0.2493606	11.2970096
H	23.3640138	-0.0484547	10.3388575
C	25.0433930	1.2191251	15.5147488
H	24.6202363	1.1486721	16.5190548
H	24.5560419	2.0426115	14.9888257
H	26.1178616	1.4357363	15.5999421
C	22.4734803	-2.0744988	17.5844738
H	21.4664133	-2.4133571	17.3325007
H	22.4094402	-1.0461553	17.9481745
H	22.8735042	-2.7151991	18.3832573
H	21.8663066	1.8570373	14.1150533
H	22.4829617	2.0311020	13.7213348

1m^{+}.(THF)$_2$: cation monomer with two THFs
72
Energy = -1836.931350232

Ca	0.0165545	-0.0044588	-0.3445284
H	-0.7621317	-0.5312136	-2.2868268
N	1.5902284	2.0707529	0.5160492
N	0.9652497	-0.4612683	2.1057774
N	1.4227582	-2.1406661	-0.3514153
N	2.0250712	0.4199802	-1.9154423
C	2.3570361	1.5864978	1.6877265
H	2.8201435	2.4350253	2.2168626
H	3.1767805	0.9613034	1.3268689
C	1.4999984	0.8047760	2.6690645
H	2.0888355	0.5997008	3.5776938
H	0.6400642	1.4065879	2.9791912
C	2.0514105	-1.4665989	1.9713144
H	2.3142579	-1.8798636	2.9579295
H	2.9428208	-0.9583108	1.5970451
C	1.6810745	-2.6079919	1.0318033
H	2.4808978	-3.3649547	1.0521990
H	0.7707371	-3.1022771	1.3834903
C	2.6970628	-1.8372053	-1.0509856
H	3.1819568	-2.7730439	-1.3711057
H	3.3748424	-1.3616880	-0.3374535
C	2.5003693	-0.9402305	-2.2680472
H	3.4456332	-0.8885316	-2.8328551
H	1.7418441	-1.3704984	-2.9279826

C	3.0952691	1.2229812	-1.2871791
H	3.8294809	1.5533327	-2.0395576
H	3.6355339	0.5907131	-0.5781547
C	2.5294005	2.4420847	-0.5720510
H	3.3568355	3.0553884	-0.1803811
H	1.9778818	3.0679542	-1.2788259
C	0.8231129	3.2731642	0.9027846
H	0.1340772	3.0344012	1.7146593
H	0.2431719	3.6245778	0.0482184
H	1.4941719	4.0796130	1.2385715
C	-0.0581407	-0.9650768	3.0472907
H	-0.3922357	-1.9553414	2.7419640
H	-0.9172907	-0.2893835	3.0503250
H	0.3464561	-1.0342651	4.0688974
C	0.6818369	-3.1786377	-1.1024012
H	0.4059171	-2.7875384	-2.0828862
H	-0.2390280	-3.4180502	-0.5677184
H	1.2825008	-4.0949869	-1.2127381
C	1.5605367	1.0866554	-3.1529722
H	1.2168926	2.0990274	-2.9267347
H	0.7159188	0.5164706	-3.5440685
H	2.3737482	1.1509517	-3.8935589
O	-2.0528980	-1.3313008	0.3430707
C	-2.3746317	-2.5378146	1.0969399
C	-3.1369108	-1.1269044	-0.6135819
C	-3.5022668	-2.5331033	-1.0508497
C	-3.4150252	-3.3197120	0.2706263
H	-1.4428991	-3.0921689	1.2323857
H	-2.7610707	-2.2471418	2.0792974
H	-3.9709489	-0.6239759	-0.1057449
H	-2.7204779	-0.5110776	-1.4134216
H	-4.4929279	-2.5891593	-1.5090201
H	-2.7574773	-2.8859381	-1.7714551
H	-4.3811622	-3.3177987	0.7826593
H	-3.1136994	-4.3591465	0.1207279
O	-1.6558997	1.7217910	-0.1931211
C	-2.0491047	2.4685493	-1.3927320
C	-3.1684086	3.4000345	-0.9406421
C	-3.8220968	2.6035050	0.1992255
C	-2.6130990	1.9751858	0.8803908
H	-2.3728925	1.7403184	-2.1424962
H	-1.1644012	2.9931983	-1.7639190
H	-3.8602562	3.6295600	-1.7541391
H	-2.7556232	4.3398327	-0.5592350
H	-4.3986046	3.2302021	0.8834900
H	-4.4805115	1.8278162	-0.2042263
H	-2.1512490	2.6632270	1.5986204
H	-2.8161571	1.0189160	1.3676253

1m^{+}**.**THF : cation monomer with one THF

59

Energy = -1604.322459658

Ca	0.2789719	0.1641078	-0.3038791
H	-0.7087164	0.0886280	-2.1818247
N	1.7907112	1.7057970	1.1357133
N	0.6547619	-1.0097720	1.9505034
N	1.3108044	-2.1243289	-0.7382585
N	2.4139285	0.5933881	-1.5434987
C	2.2853436	0.8745463	2.2638876
H	2.7175158	1.5180997	3.0456489
H	3.0986411	0.2469553	1.8918269
C	1.1985503	0.0073277	2.8892470
H	1.6025942	-0.4756181	3.7923666
H	0.3597058	0.6319951	3.2106206
C	1.6446472	-2.0966098	1.7294013
H	1.7001377	-2.7460333	2.6165172
H	2.6302676	-1.6411498	1.6079792
C	1.3125286	-2.9369809	0.5036403
H	2.0267593	-3.7711956	0.4300305
H	0.3179941	-3.3794706	0.6067918
C	2.7019236	-1.8742145	-1.1981173
H	3.1056441	-2.7741936	-1.6871308
H	3.3242259	-1.6895107	-0.3183790
C	2.7986278	-0.6996969	-2.1658826
H	3.8216043	-0.6476382	-2.5699022
H	2.1214171	-0.8566702	-3.0095189
C	3.4625442	1.0862114	-0.6205323
H	4.3245740	1.4783540	-1.1824395
H	3.8272726	0.2390422	-0.0333623
C	2.9338522	2.1743000	0.3070695
H	3.7529492	2.5417320	0.9438113
H	2.5806965	3.0272775	-0.2786387
C	1.0696758	2.8845850	1.6675028
H	0.2250142	2.5621647	2.2777294
H	0.6871980	3.4800318	0.8355109
H	1.7344656	3.5121600	2.2798593
C	-0.5874450	-1.5799229	2.5188869
H	-1.0424505	-2.2754243	1.8098136
H	-1.2953246	-0.7748303	2.7229208
H	-0.3869325	-2.1178830	3.4573940
C	0.5497721	-2.8332195	-1.7930778
H	0.5093711	-2.2185357	-2.6926483
H	-0.4753963	-2.9917342	-1.4499052
H	1.0096044	-3.8064428	-2.0242072
C	2.1502597	1.5839212	-2.6122053
H	1.8634704	2.5423120	-2.1723253
H	1.3185496	1.2215242	-3.2202557
H	3.0410635	1.7400497	-3.2391704
O	-1.5685182	1.5024344	0.4357105
C	-2.6679716	1.2818164	1.3790583
C	-1.9777333	2.5022147	-0.5602431
C	-3.4956816	2.5127923	-0.4955308
C	-3.7485662	2.2935885	1.0040720
H	-3.0126050	0.2501228	1.2530219
H	-2.2772431	1.4189424	2.3912916
H	-1.5486596	3.4690189	-0.2708210
H	-1.5710157	2.1661746	-1.5191743
H	-3.9177091	3.4499679	-0.8656209
H	-3.9034676	1.6839951	-1.0833905
H	-3.6040165	3.2288318	1.5541280

H	-4.7497245	1.9142432	1.2208362

1m^{+} : calcium hydride cation monomer
46
Energy = -1371.697634832

Ca	16.6823803	11.1154613	14.8814267
H	17.9970915	9.5473815	14.8456210
N	17.0154998	13.1729864	13.4909830
N	14.7443437	11.2096885	13.2994186
N	14.6179710	11.0629700	16.2985326
N	16.8873188	13.0246166	16.4925877
C	15.7102222	13.4777807	12.8482564
H	15.8423615	14.2231433	12.0495243
H	15.0619162	13.9334483	13.6015249
C	15.0398018	12.2370110	12.2648361
H	14.1215935	12.5372247	11.7385969
H	15.6932055	11.7704939	11.5225165
C	13.5473973	11.5897862	14.0946619
H	12.6303297	11.4196229	13.5106515
H	13.6012381	12.6638424	14.2922942
C	13.4497142	10.8212508	15.4096676
H	12.5109306	11.0903596	15.9160049
H	13.4084557	9.7461293	15.2143930
C	14.5096310	12.3879401	16.9633100
H	13.7630700	12.3504298	17.7709471
H	14.1413557	13.1057904	16.2255165
C	15.8402580	12.8661467	17.5375966
H	15.6810831	13.8109871	18.0779689
H	16.2168837	12.1430079	18.2662052
C	16.6718653	14.2737840	15.7160646
H	16.9763809	15.1493906	16.3089447
H	15.5989955	14.3746331	15.5315703
C	17.4317379	14.2807104	14.3925058
H	17.2938075	15.2560744	13.9029721
H	18.5040014	14.1651220	14.5735875
C	18.0600197	12.9432622	12.4663001
H	17.7672538	12.1189603	11.8121082
H	18.9971455	12.6751031	12.9609476
H	18.2220412	13.8401374	11.8510924
C	14.5440143	9.8922139	12.6525728
H	14.3399184	9.1313597	13.4092446
H	15.4551458	9.6109906	12.1181531
H	13.7040524	9.9176382	11.9434249
C	14.7126271	9.9800508	17.3047309
H	15.5780774	10.1442312	17.9506913
H	14.8412971	9.0229581	16.7925915
H	13.8099057	9.9345337	17.9309411
C	18.2270058	13.0282295	17.1244302
H	19.0009616	13.1274455	16.3599165
H	18.3801900	12.0820998	17.6498447
H	18.3308509	13.8572721	17.8393481

1^{2+}**.**(THF)$_2$: cation dimer with two THFs
118
Energy = -3208.662083857

Ca	-3.6231949	1.6730924	-1.7451178
N	-4.8470917	2.2804230	0.4869791
N	-4.1310102	4.2680475	-1.6153279
N	-5.2824777	2.3788971	-3.7510116
N	-5.9828959	0.3735729	-1.6513663
H	-3.0848149	0.0053588	-0.3295516
H	-1.4227779	1.4862177	-1.4991039
C	-5.4926388	3.6082296	0.3754124
C	-5.8372825	1.1979032	0.7090184
C	-3.9366422	2.2733765	1.6502219
C	-4.5413074	4.6283048	-0.2322749
C	-5.2286124	4.5987475	-2.5640077
C	-2.9380534	5.0715560	-1.9616420
C	-5.1273815	3.8494539	-3.8854690
C	-6.6714652	2.0275518	-3.3715083
C	-4.9888319	1.7713780	-5.0697994
C	-6.7734948	0.5897411	-2.8883292
C	-6.7260178	0.9278299	-0.4916514
C	-5.7931466	-1.0776756	-1.4495722
Ca	-0.8364973	-0.1311758	-0.1043765
H	-5.8336419	3.9657781	1.3595871
H	-6.3826118	3.5133075	-0.2508438
H	-6.4701947	1.4329216	1.5802804
H	-5.2577339	0.3011499	0.9472952
H	-3.2756251	3.1384916	1.6173947
H	-3.3386778	1.3610184	1.6091760
H	-4.5024402	2.3070019	2.5941691
H	-5.0061092	5.6258568	-0.2161510
H	-3.6295001	4.6969583	0.3687261
H	-5.2309684	5.6799717	-2.7702306
H	-6.1791270	4.3760932	-2.0753015
H	-2.6933268	4.9366443	-3.0145476
H	-2.0886142	4.7408945	-1.3581097
H	-3.1137899	6.1422079	-1.7789605
H	-5.8836204	4.2562767	-4.5767359
H	-4.1488606	4.0225921	-4.3429396
H	-7.3531866	2.1798048	-4.2233953
H	-7.0007270	2.7042377	-2.5799117
H	-5.0220687	0.6816045	-4.9998200
H	-3.9909675	2.0747071	-5.3831533
H	-5.7263276	2.0936503	-5.8212208
H	-7.8315943	0.3296538	-2.7280002
H	-6.3901911	-0.0945881	-3.6497761
H	-7.5237555	0.2299988	-0.1922628
H	-7.2235436	1.8472962	-0.8076827
H	-5.1523341	-1.2381549	-0.5800633
H	-5.3038887	-1.5001280	-2.3302592
H	-6.7568341	-1.5904997	-1.3033990
N	-1.7674102	-1.9007693	1.5571183
N	1.4137785	-1.0761748	-1.0657679
N	-1.2431684	-2.3733541	-1.4281072
N	0.9307108	-0.6733238	1.9558130
C	-2.8039286	-1.2888359	2.4144220
C	-2.4176700	-2.9296048	0.7088810
C	-0.7144815	-2.5061300	2.4048775

C	1.2406889	-2.4977726	-1.4598725
C	2.4579276	-0.9616281	-0.0128351
C	1.8478654	-0.3066085	-2.2520839
C	-1.5192017	-3.4242032	-0.4156049
C	-0.0492327	-2.7301351	-2.2304151
C	-2.4130676	-2.2595835	-2.3202850
C	0.2185827	-1.4601600	2.9940354
C	2.0030914	-1.4946147	1.3399150
C	1.5539408	0.4894675	2.6261670
H	-2.3673261	-0.5008547	3.0332655
H	-3.5597102	-0.8478226	1.7617372
H	-3.2657755	-2.0364682	3.0780665
H	-2.7399564	-3.7883886	1.3195208
H	-3.3127530	-2.4613265	0.2894327
H	-1.1663478	-3.0881012	3.2238833
H	-0.1448004	-3.2123566	1.7969529
H	2.0958205	-2.8291092	-2.0692832
H	1.2432877	-3.1092370	-0.5554488
H	3.3717071	-1.4943564	-0.3202658
H	2.7183935	0.0974413	0.0722675
H	1.0676191	-0.3270500	-3.0156264
H	2.0290178	0.7295643	-1.9678137
H	2.7716058	-0.7231818	-2.6814915
H	-1.9937034	-4.2929666	-0.8982622
H	-0.5667254	-3.7748712	-0.0122769
H	-0.0912126	-3.7804199	-2.5590962
H	-0.0676327	-2.1157760	-3.1357633
H	-3.2688936	-1.9243326	-1.7371660
H	-2.2078223	-1.5147784	-3.0904129
H	-2.6405229	-3.2203844	-2.8084006
H	0.9356307	-1.9559215	3.6682837
H	-0.3483263	-0.7503704	3.6036162
H	2.8748213	-1.5434991	2.0114459
H	1.6381405	-2.5184670	1.2346556
H	2.1303803	1.0725695	1.9090829
H	0.7749232	1.1299559	3.0448132
H	2.2188277	0.1577762	3.4393514
H	0.7596379	2.4840309	-1.5090191
C	1.1227471	2.7652182	-0.5157470
C	0.9573388	4.2682161	-0.2404166
O	0.3044512	2.0628610	0.4691708
H	2.1509657	2.4211338	-0.3723926
C	0.2687521	4.3129289	1.1347441
H	0.3160062	4.7234665	-1.0013085
H	1.9151246	4.7918563	-0.2499909
C	-0.5566286	3.0375006	1.0996906
H	1.0035392	4.2626458	1.9437208
H	-0.3435729	5.2075213	1.2745124
H	-1.4556673	3.1569753	0.4781012
H	-0.8336875	2.6434102	2.0806617
H	-1.2025198	0.2317661	-3.5866482
C	-1.6503571	0.7536969	-4.4449797
C	-0.5925727	1.1377482	-5.4666300
O	-2.1872684	2.0117868	-3.9729521
H	-2.4839169	0.1723666	-4.8477975

C	0.0528446	2.3550770	-4.7849195
H	-1.0645218	1.4210834	-6.4124423
H	0.1181960	0.3299758	-5.6603480
C	-1.1163506	3.0057324	-4.0337430
H	0.8198482	2.0271727	-4.0769905
H	0.5158532	3.0441097	-5.4942812
H	-0.8526628	3.2748098	-3.0075645
H	-1.5209537	3.8727462	-4.5636844

1^{2+}.THF : THF-coordinated stable cation dimer
105
Energy = -2976.072868336

Ca	-1.7411574	-0.0216885	0.1275703
H	0.0124080	-0.3437867	-1.2671951
N	-1.6110930	-2.5615533	0.3614107
N	-2.7002087	-0.6691504	2.4331883
N	-4.3168397	0.7632066	0.3514812
N	-3.1729114	-1.0900137	-1.7535998
C	-2.4476852	-2.9798130	1.5103720
H	-2.2110279	-4.0141690	1.8057280
H	-3.4934998	-2.9738061	1.1936292
C	-2.2578749	-2.0579165	2.7112593
H	-2.7947010	-2.4735647	3.5782736
H	-1.1994430	-2.0077660	2.9818074
C	-4.1796141	-0.5860463	2.4598274
H	-4.5471327	-0.6118163	3.4977923
H	-4.5814170	-1.4727293	1.9638094
C	-4.7016290	0.6724397	1.7827628
H	-5.7969931	0.7133719	1.8950828
H	-4.2996381	1.5599077	2.2804471
C	-5.1112933	-0.1935430	-0.4585030
H	-6.1437483	0.1716645	-0.5778742
H	-5.1743419	-1.1370538	0.0886769
C	-4.5020953	-0.4334521	-1.8334042
H	-5.1985611	-1.0326985	-2.4403625
H	-4.3655780	0.5177798	-2.3556910
C	-3.3385299	-2.5331115	-1.4448930
H	-3.6645137	-3.0789019	-2.3442029
H	-4.1395828	-2.6360024	-0.7086163
C	-2.0624188	-3.1699416	-0.9129014
H	-2.2173815	-4.2546467	-0.8014183
H	-1.2502049	-3.0278582	-1.6320211
C	-0.2083452	-2.9341122	0.6032273
H	0.1479732	-2.4315435	1.5027293
H	0.3806793	-2.5925401	-0.2516599
H	-0.0854370	-4.0229376	0.7179251
C	-2.1219375	0.2385260	3.4499637
H	-2.4586776	1.2624896	3.2678317
H	-1.0339170	0.2126158	3.3572241
H	-2.4293334	-0.0527798	4.4661583
C	-4.6039631	2.1384916	-0.1086347
H	-4.3572077	2.2419576	-1.1662425
H	-4.0014580	2.8464391	0.4625138
H	-5.6685742	2.3856538	0.0251492
C	-2.4702701	-0.9306680	-3.0466859

H	-1.4684123	-1.3560242	-2.9752882
H	-2.3653213	0.1344439	-3.2680767
H	-3.0277127	-1.4130382	-3.8646822
Ca	1.8050663	-0.0799158	-0.0276407
H	0.1412802	0.1724027	1.3712827
N	3.3030216	1.4867325	1.3356069
N	3.0417562	1.4847609	-1.6264031
N	3.1340475	-1.5710888	-1.6414263
N	3.4155320	-1.5618380	1.3441292
C	4.2918973	2.1368747	0.4395719
H	4.7596415	2.9977853	0.9408024
H	5.0911133	1.4202659	0.2364138
C	3.6655955	2.6010333	-0.8692814
H	4.4285650	3.1105510	-1.4770416
H	2.8815173	3.3372937	-0.6678601
C	4.0883702	0.6605948	-2.2852849
H	4.5074110	1.2028830	-3.1468099
H	4.9069091	0.5183479	-1.5751399
C	3.5655060	-0.6897293	-2.7583438
H	4.3428554	-1.1840138	-3.3609914
H	2.6978058	-0.5495080	-3.4088620
C	4.3099596	-2.1316942	-0.9276565
H	4.7533237	-2.9571874	-1.5047710
H	5.0731077	-1.3528620	-0.8606977
C	3.9531272	-2.6355566	0.4661196
H	4.8403858	-3.1041827	0.9187097
H	3.1839324	-3.4105950	0.3977562
C	4.5109909	-0.6557768	1.7807380
H	5.1361596	-1.1589691	2.5341991
H	5.1547358	-0.4557086	0.9206717
C	3.9923596	0.6557494	2.3561628
H	4.8296531	1.2098322	2.8068921
H	3.2725702	0.4589881	3.1551919
C	2.4830592	2.5096741	2.0212051
H	2.0166728	3.1645469	1.2823091
H	1.6956507	2.0111200	2.5910715
H	3.0955459	3.1287190	2.6935575
C	2.1250757	2.0376019	-2.6473124
H	1.5843665	1.2298880	-3.1435395
H	1.3970184	2.6829000	-2.1528545
H	2.6702355	2.6284486	-3.3984542
C	2.3160833	-2.6685606	-2.2064390
H	2.0748129	-3.3935025	-1.4265583
H	1.3872772	-2.2493146	-2.6017877
H	2.8569655	-3.1934218	-3.0078936
C	2.7926041	-2.1765948	2.5392511
H	2.2617118	-1.4185994	3.1194666
H	2.0781472	-2.9372002	2.2219684
H	3.5487211	-2.6542053	3.1799095
O	-1.4638330	2.3095127	-0.3912529
C	-1.0938670	3.3305239	0.5798631
C	-0.3125419	4.3576903	-0.2240928
C	-1.0625390	4.3542789	-1.5663219
C	-1.4842949	2.8912272	-1.7339593
H	-0.5253591	2.8246931	1.3635813
H	-2.0050118	3.7673652	1.0076973
H	0.7228497	4.0268829	-0.3593041
H	-0.2987932	5.3399411	0.2526462
H	-1.9434508	4.9996227	-1.5045236
H	-0.4463078	4.6991625	-2.3997112
H	-2.4937494	2.7895107	-2.1414213
H	-0.7921434	2.3039160	-2.3431299

1^{2+} : double Ca-H-Ca bridged cation dimer
92
Energy = -2743.459641286

Ca	16.7865530	11.0111242	14.8701071
H	18.9377544	10.5680113	14.7936119
N	17.0779593	13.0815046	13.4731944
N	14.8342476	11.1354462	13.2745436
N	14.7220986	10.9818193	16.3028985
N	16.9582616	12.9322004	16.5001104
C	15.7728915	13.4138973	12.8490116
H	15.9018587	14.1727878	12.0621601
H	15.1341264	13.8596827	13.6161339
C	15.0911758	12.1856727	12.2538780
H	14.1570245	12.4918055	11.7599099
H	15.7265295	11.7411333	11.4831032
C	13.6438553	11.4846780	14.0940495
H	12.7219792	11.3036658	13.5205026
H	13.6798289	12.5577226	14.3010521
C	13.5741164	10.7010108	15.4014846
H	12.6197504	10.9231982	15.9024074
H	13.5855268	9.6274514	15.1942402
C	14.5807879	12.3075080	16.9552747
H	13.8248603	12.2674474	17.7543995
H	14.2134943	13.0158013	16.2078401
C	15.9007407	12.8037249	17.5375894
H	15.7344854	13.7619981	18.0516531
H	16.2671584	12.0996658	18.2897716
C	16.7624941	14.1752907	15.7084989
H	17.0773802	15.0519803	16.2948366
H	15.6914413	14.2901131	15.5213114
C	17.5262242	14.1643177	14.3875219
H	17.4246551	15.1491010	13.9069420
H	18.5923690	14.0069381	14.5728549
C	18.1097731	12.8518715	12.4374428
H	17.7956230	12.0460998	11.7689873
H	19.0389095	12.5514966	12.9271850
H	18.2835655	13.7560471	11.8352986
C	14.6349608	9.8279195	12.6090281
H	14.5015898	9.0455459	13.3582403
H	15.5246850	9.5872529	12.0212487
H	13.7603043	9.8467802	11.9419157
C	14.8261290	9.9085606	17.3165630
H	15.6795745	10.0964798	17.9730383
H	14.9873060	8.9569779	16.8052264
H	13.9177274	9.8493761	17.9343553
C	18.2927639	12.9342844	17.1411967
H	19.0723576	12.9692188	16.3780225

H	18.4162575	12.0097663	17.7114692
H	18.4102922	13.7914963	17.8207652
Ca	19.0453335	8.3713159	14.8282651
H	16.8939226	8.8142024	14.9039381
N	18.7566438	6.3009175	16.2256664
N	20.9977521	8.2499893	16.4240771
N	21.1095428	8.4026328	13.3955933
N	18.8760348	6.4493206	13.1988678
C	20.0619820	5.9704596	16.8503884
H	19.9336782	5.2118144	17.6375767
H	20.7014565	5.5249999	16.0836646
C	20.7421399	7.1997666	17.4450451
H	21.6766685	6.8949768	17.9391335
H	20.1062224	7.6436909	18.2157001
C	22.1885844	7.9020761	15.6046817
H	23.1102138	8.0846551	16.1781313
H	22.1541795	6.8288836	15.3982033
C	22.2572124	8.6852384	14.2968808
H	23.2118185	8.4640804	13.7959681
H	22.2444521	9.7588541	14.5037097
C	21.2526352	7.0770493	12.7433539
H	22.0084339	7.1181162	11.9441667
H	21.6210173	6.3693229	13.4907875
C	19.9332900	6.5789642	12.1612444
H	20.1007265	5.6207884	11.6473945
H	19.5659027	7.2823788	11.4089118
C	19.0735300	5.2067004	13.9907853
H	18.7597818	4.3294498	13.4046687
H	20.1447525	5.0933498	14.1778566
C	18.3099827	5.2169760	15.3118756
H	18.4132051	4.2325900	15.7929034
H	17.2435571	5.3725166	15.1266188
C	17.7242794	6.5298272	17.2610355
H	18.0372603	7.3363967	17.9290909
H	16.7948555	6.8287138	16.7709007
H	17.5515033	5.6258209	17.8637230
C	21.1952931	9.5579586	17.0892165
H	21.3271383	10.3404417	16.3398457
H	20.3054011	9.7972714	17.6773045
H	22.0702137	9.5406110	17.7560406
C	21.0041151	9.4755360	12.3816948
H	20.1507261	9.2865119	11.7254569
H	20.8419191	10.4269975	12.8929367
H	21.9123104	9.5355926	11.7636847
C	17.5415416	6.4451213	12.5577973
H	16.7619230	6.4096249	13.3209352
H	17.4169250	7.3691642	11.9870035
H	17.4251265	5.5873608	11.8787227

Aa^{2+} : from dimeric Markovnikov addition
112
Energy = -3055.711520750

Ca	0.2915748	0.1387193	1.5640682
Ca	-0.9240040	0.2763133	-1.7898255
H	-1.1873481	1.0005253	0.2183338
N	2.2484841	0.7801665	3.1901890
N	0.0458541	-0.5588293	-4.1274831
N	-1.8615975	-0.1735224	2.8354467
N	-2.8964765	1.9041802	-2.0893820
N	-0.1912025	2.4257856	2.6898658
N	-0.0454168	2.2655817	-3.0214649
N	0.6041767	-1.7999609	3.3992102
N	-2.8002420	-0.9314044	-3.0918545
C	-2.3500468	1.2076230	3.0931036
C	-2.2917374	3.2160282	-2.4330128
C	2.6232141	-0.4328194	3.9636600
C	-0.9442161	-1.5168633	-4.6862473
C	-1.2801844	2.1331901	3.6595119
C	-1.1800906	3.1146975	-3.4706087
C	1.4107979	-1.1885955	4.4882687
C	-2.3876252	-1.0709364	-4.5151336
C	3.4484103	1.2762727	2.4787068
C	1.3712584	-1.2188991	-4.1636393
C	-2.8686136	-0.8687889	2.0033168
C	-3.7232838	2.0769551	-0.8751124
C	1.7648166	1.8349104	4.1195209
C	0.1083014	0.6832262	-4.9425640
C	-1.6451179	-0.9194115	4.0986261
C	-3.7413932	1.3862081	-3.1919794
C	1.0088361	2.9377617	3.3990114
C	0.7697789	1.8286041	-4.1856223
C	-0.7566737	-2.1403719	3.8970254
C	-4.0339346	-0.0995293	-3.0213836
C	-0.6460385	3.4631898	1.7395791
C	0.8023913	3.0084013	-2.0638875
C	1.2657100	-3.0610002	2.9892585
C	-3.0964421	-2.2707609	-2.5314632
H	-3.2093356	1.1877125	3.7813489
H	-3.0571497	3.9171439	-2.8007947
H	3.2761236	-0.1637670	4.8082309
H	-0.7547258	-1.6883202	-5.7576267
H	-2.7001596	1.5924851	2.1314841
H	-1.8978853	3.6340111	-1.5015923
H	3.2100992	-1.0741302	3.2986485
H	-0.7816806	-2.4728956	-4.1791226
H	3.8491195	0.4835348	1.8437330
H	1.3423740	-2.1288413	-3.5603493
H	-2.9361666	-0.3431335	1.0475699
H	-3.0767796	2.3903841	-0.0553604
H	1.1176798	1.3681091	4.8648979
H	-0.9088066	0.9670690	-5.2229876
H	-1.1925906	-0.2411153	4.8255639
H	-3.2224221	1.5598222	-4.1381108
H	-0.8342569	1.6894735	4.5528049
H	-1.5698370	2.6923247	-4.3996721
H	0.7681487	-0.5153069	5.0591370
H	-2.5443988	-0.1091113	-5.0079248
H	4.2284099	1.5887073	3.1890959
H	1.6495180	-1.4823379	-5.1950398
H	-3.8521057	-0.8783213	2.4967868

H	-4.5171319	2.8215397	-1.0394754
H	2.6133820	2.2727333	4.6670984
H	0.6552950	0.4988032	-5.8794443
H	-2.6059587	-1.2436780	4.5267484
H	-4.6920480	1.9387764	-3.2464551
H	-1.7573973	3.0717257	3.9811764
H	-0.8232383	4.1276779	-3.7096225
H	1.7474713	-1.9679112	5.1885310
H	-3.0432598	-1.7933744	-5.0241063
H	3.1884881	2.1337312	1.8530605
H	2.1350270	-0.5542700	-3.7561584
H	-2.5603923	-1.9000224	1.8205197
H	-4.1866651	1.1286576	-0.5962784
H	-1.5004630	3.0891011	1.1766776
H	0.2012380	3.3147270	-1.2056459
H	2.2728582	-2.8525347	2.6249837
H	-2.2151061	-2.9116716	-2.6059626
H	1.6515666	3.4089597	2.6497660
H	1.7444139	1.5135331	-3.8024876
H	-1.2065047	-2.8142527	3.1614123
H	-4.4969664	-0.2827981	-2.0483113
H	0.7340755	3.7216446	4.1217448
H	0.9506845	2.6692827	-4.8711018
H	-0.6925400	-2.6993440	4.8435872
H	-4.7570622	-0.4222867	-3.7849550
H	0.1613906	3.6787826	1.0353335
H	1.6048493	2.3532942	-1.7130354
H	0.6966598	-3.5353913	2.1892665
H	-3.3743702	-2.1717909	-1.4782210
H	-0.9161231	4.3919473	2.2652370
H	1.2474231	3.9022014	-2.5240881
H	1.3371222	-3.7573160	3.8380336
H	-3.9252744	-2.7533328	-3.0696103
C	0.8366465	-1.1578265	-0.5792645
C	-0.1262004	-2.2717769	-0.1303788
H	0.9425967	-1.2552014	-1.6745559
H	-0.3686172	-2.2226048	0.9458356
H	-1.1008570	-2.2001116	-0.6364634
C	2.2713705	-1.3058839	-0.0568187
C	2.9482477	-2.6723316	-0.2869843
C	3.1522859	-0.2089009	-0.6790218
H	2.3100360	-1.1555309	1.0496376
C	4.3315826	-2.7400392	0.3734309
H	3.0531543	-2.8177053	-1.3710015
H	2.3170422	-3.4854936	0.0845125
C	4.6213124	-0.3809139	-0.4019856
H	2.9766419	-0.1972279	-1.7656016
H	2.8221908	0.7806060	-0.3286159
C	5.1469445	-1.5072422	0.0928603
H	4.8766798	-3.6286281	0.0301551
H	4.2278200	-2.8686775	1.4631416
H	5.2684911	0.4698043	-0.6112112
H	6.2150277	-1.5560496	0.2992115
H	0.2179873	-3.3045861	-0.3001014

A^{2+} : from dimeric anti-Markovnikov addition

112

Energy = -3055.714120401

Ca	-0.2132748	-0.8921742	1.8948104
Ca	0.3768894	-0.6338887	-1.6079448
H	-0.8457430	0.0716769	0.0456310
N	1.0725859	-2.2558071	3.7381951
N	0.6756659	-2.3431664	-3.6193007
N	-1.3284252	1.2115046	2.8574883
N	0.1500293	1.8042651	-2.3949702
N	1.6058493	0.5716998	2.8108992
N	2.4853500	0.0148441	-2.8792548
N	-1.9040413	-1.6164144	3.7207407
N	-1.6695646	-0.5768571	-3.0324236
C	-0.2614185	2.2311999	3.0118347
C	1.4712936	2.3042102	-2.8530925
C	0.0517588	-2.9453571	4.5704314
C	-0.6592450	-2.5133018	-4.2523457
C	1.0183434	1.6702314	3.6206669
C	2.2525570	1.2672519	-3.6467517
C	-1.1428658	-2.0692811	4.9161291
C	-1.4489386	-1.2143790	-4.3537355
C	2.0426766	-3.2753610	3.2786049
C	1.2277874	-3.6906558	-3.3448676
C	-2.3806338	1.7605445	1.9734810
C	-0.3696642	2.7327976	-1.3691100
C	1.7947223	-1.2234104	4.5276615
C	1.6040087	-1.6315430	-4.5370725
C	-1.9181632	0.8474433	4.1681620
C	-0.8171009	1.7278497	-3.5219485
C	2.5406049	-0.2411658	3.6326975
C	2.8303337	-1.0918094	-3.8114589
C	-2.7354737	-0.4354089	4.0834868
C	-2.0275910	0.8577517	-3.1920545
C	2.3117981	1.1140205	1.6324005
C	3.5913348	0.2074659	-1.9147836
C	-2.8025569	-2.7030768	3.2655244
C	-2.7419463	-1.2774273	-2.2901486
H	-0.6089478	3.0745588	3.6290885
H	1.3594179	3.2136529	-3.4634675
H	0.5004761	-3.3175642	5.5047780
H	-0.5605165	-2.9488800	-5.2587491
H	-0.0578344	2.6320383	2.0142752
H	2.0308416	2.5925116	-1.9578370
H	-0.2771901	-3.8239895	4.0059629
H	-1.2088830	-3.2406438	-3.6468023
H	1.5253351	-4.0198192	2.6698339
H	0.5494383	-4.2343906	-2.6862529
H	-1.9411232	1.9679138	0.9987337
H	0.3163563	2.7510454	-0.5191346
H	1.0683830	-0.6835521	5.1402016
H	1.0610446	-0.8096132	-5.0097969
H	-1.1070978	0.7266508	4.8910900
H	-0.2959808	1.3268034	-4.3953131
H	0.8193338	1.2795255	4.6212827

H	1.7098495	1.0087510	-4.5588556
H	-0.8127089	-1.1835364	5.4633892
H	-0.9203390	-0.5012948	-4.9907875
H	2.5157932	-3.7796757	4.1345334
H	1.3530023	-4.2596642	-4.2781793
H	-2.8163662	2.6768260	2.4007374
H	-0.4691795	3.7526996	-1.7709690
H	2.5043384	-1.6988761	5.2218193
H	1.9261306	-2.3045077	-5.3461592
H	-2.5591704	1.6603446	4.5434676
H	-1.1622443	2.7367213	-3.7955495
H	1.7460261	2.4871768	3.7391484
H	3.2116738	1.7034588	-3.9636597
H	-1.8061776	-2.6265029	5.5946234
H	-2.4112028	-1.4218513	-4.8455241
H	2.8196538	-2.8102143	2.6704887
H	2.1973684	-3.6092925	-2.8502863
H	-3.1748967	1.0253810	1.8320603
H	-1.3391668	2.3841410	-1.0157443
H	1.6103670	1.6807950	1.0140837
H	3.3688119	1.0526473	-1.2591415
H	-2.2234733	-3.6046416	3.0601455
H	-2.4831844	-2.3334335	-2.1746211
H	3.1959918	-0.7819663	2.9447645
H	3.3010349	-1.8836267	-3.2223201
H	-3.5138066	-0.3374220	3.3222238
H	-2.4765893	1.1819777	-2.2494064
H	3.1823693	0.4056675	4.2482673
H	3.5741510	-0.7549891	-4.5484818
H	-3.2468245	-0.6075445	5.0422975
H	-2.7892208	0.9822827	-3.9761346
H	2.7055143	0.2795419	1.0456507
H	3.7035518	-0.6947794	-1.3065974
H	-3.3093584	-2.3954095	2.3470921
H	-2.8287984	-0.8282759	-1.2973387
H	3.1416151	1.7763991	1.9189233
H	4.5426717	0.4106550	-2.4271747
H	-3.5587772	-2.9389491	4.0284623
H	-3.7055610	-1.2113028	-2.8159088
C	0.8352748	-2.4766933	0.1246565
C	-0.4500621	-3.2257026	0.5544934
H	1.6916184	-2.8790439	0.6798320
H	1.0563155	-2.8053834	-0.9097140
H	-0.4186516	-3.4341013	1.6406666
H	-1.3522603	-2.5889060	0.4033315
C	-0.7663024	-4.5590683	-0.1417444
C	0.3595125	-5.5833692	0.0374793
C	-2.0980338	-5.1692528	0.3119688
H	-0.8565725	-4.3463945	-1.2192298
C	-0.0254130	-6.9408198	-0.4905351
H	0.6189637	-5.6570357	1.1066380
H	1.2715989	-5.2312220	-0.4600328
C	-2.4669580	-6.3795802	-0.5564833
H	-2.0032198	-5.4890253	1.3591238
H	-2.8963194	-4.4172451	0.2744989
C	-1.2852717	-7.2880055	-0.7771750
H	0.7795065	-7.6572481	-0.6490577
H	-3.2854538	-6.9458671	-0.0938350
H	-2.8554555	-6.0398915	-1.5291943
H	-1.4902222	-8.2770283	-1.1846914

Ba^{2+} : from dimeric anti-Markovnikov addition

108

Energy = -3053.323166931

Ca	-0.0831514	-0.8150377	1.8421300
Ca	0.0585813	-0.6896404	-1.6673027
H	-1.1234598	-0.0893374	0.0637592
N	0.8692527	-2.5059282	3.6355821
N	-0.0285864	-2.4194914	-3.5823307
N	-1.0382557	1.3278815	2.8952855
N	0.2752125	1.7145367	-2.4637783
N	1.7727072	0.3525125	3.0644655
N	2.2464154	-0.4679336	-2.9499255
N	-1.9814434	-1.5346838	3.3525416
N	-1.9920325	-0.2262324	-3.0542601
C	0.0935952	2.1957497	3.3085656
C	1.6513579	1.9564683	-2.9715459
C	-0.3039288	-3.1535134	4.2811196
C	-1.3618763	-2.3305658	-4.2393141
C	1.2337496	1.4169879	3.9508785
C	2.1890353	0.7732754	-3.7649505
C	-1.4494122	-2.1951631	4.5734632
C	-1.8660516	-0.8988199	-4.3703977
C	1.7923242	-3.5852402	3.2129704
C	0.2012448	-3.8298110	-3.1898050
C	-1.9264840	2.1048628	2.0021943
C	-0.0147800	2.6735490	-1.3769833
C	1.5831923	-1.6140023	4.5867392
C	1.0407579	-1.9973670	-4.5279060
C	-1.8108116	0.8588111	4.0730264
C	-0.7235772	1.8770888	-3.5544374
C	2.5219214	-0.6508781	3.8676584
C	2.3533223	-1.6641131	-3.8271333
C	-2.7332651	-0.3055053	3.7289836
C	-2.0717436	1.2484191	-3.2156903
C	2.6546090	0.9354636	2.0297311
C	3.4157629	-0.4192500	-2.0435437
C	-2.8918456	-2.4540797	2.6307671
C	-3.1952993	-0.7068993	-2.3380397
H	-0.2419425	2.9784917	4.0063841
H	1.6858635	2.8612977	-3.5965167
H	-0.0070498	-3.6457301	5.2204650
H	-1.3342071	-2.7969280	-5.2357047
H	0.4484503	2.7069920	2.4084000
H	2.2881750	2.1470633	-2.1028506
H	-0.6386964	-3.9452803	3.6026437
H	-2.0567389	-2.9186191	-3.6333887
H	1.2954079	-4.2321203	2.4888998
H	-0.5800820	-4.1487467	-2.4977841
H	-1.3467326	2.4596976	1.1507036

H	0.6989089	2.5212049	-0.5626017
H	0.8422831	-1.0491080	5.1582033
H	0.6822382	-1.1280770	-5.0845212
H	-1.1049231	0.5576563	4.8516905
H	-0.3140309	1.4221652	-4.4598209
H	0.8913664	0.9432930	4.8737626
H	1.5496886	0.5853448	-4.6303667
H	-1.1195090	-1.4151737	5.2632931
H	-1.1795233	-0.3144557	-4.9879971
H	2.1049360	-4.1886923	4.0785012
H	0.1877268	-4.4896011	-4.0699282
H	-2.3619009	2.9654646	2.5330356
H	0.0657653	3.7132905	-1.7276042
H	2.1557763	-2.2107603	5.3127726
H	1.2218659	-2.7916523	-5.2676418
H	-2.4103873	1.6828642	4.4897447
H	-0.8708677	2.9450879	-3.7756776
H	2.0329586	2.1182534	4.2335300
H	3.1873409	1.0216177	-4.1558102
H	-2.2513050	-2.7474787	5.0857059
H	-2.8337999	-0.9070252	-4.8948901
H	2.6816112	-3.1612627	2.7446702
H	1.1641965	-3.9315976	-2.6878360
H	-2.7261249	1.4703462	1.6193713
H	-1.0207727	2.5001051	-0.9938872
H	2.0965387	1.6649778	1.4357681
H	3.3373618	0.4455118	-1.3804241
H	-2.3785930	-3.3942578	2.4171360
H	-3.1485162	-1.7922785	-2.2182958
H	3.1729234	-1.2003950	3.1826673
H	2.6655254	-2.5017626	-3.1959983
H	-3.3718415	-0.0417270	2.8821531
H	-2.4478294	1.6528857	-2.2719917
H	3.1727270	-0.1522505	4.6005445
H	3.1397904	-1.5219682	-4.5838038
H	-3.3973409	-0.5097479	4.5817773
H	-2.7995740	1.5172013	-3.9963834
H	3.0079846	0.1384499	1.3694551
H	3.4390101	-1.3245813	-1.4336341
H	-3.1932102	-1.9943102	1.6850824
H	-3.2161987	-0.2508883	-1.3456674
H	3.5222792	1.4446088	2.4732415
H	4.3558092	-0.3402441	-2.6091276
H	-3.7895663	-2.6756440	3.2257135
H	-4.1150096	-0.4567516	-2.8877094
C	1.1073953	-2.2453230	0.0625078
C	0.0078165	-3.2982062	0.2723085
H	1.9210829	-2.4213680	0.7835984
H	1.5991449	-2.4625333	-0.9064801
H	-0.4152479	-3.2067397	1.2931984
H	-0.8641642	-3.0894271	-0.3756716
C	0.3352823	-4.7798511	0.1027994
C	1.6450998	-5.2272924	-0.0829614
C	-0.6990424	-5.7259704	0.1265904
C	1.9201812	-6.5880457	-0.2417428
H	2.4540079	-4.5020764	-0.1054967
C	-0.4317552	-7.0846068	-0.0328923
H	-1.7268311	-5.3926791	0.2630553
C	0.8834183	-7.5208353	-0.2184161
H	2.9456043	-6.9176822	-0.3862202
H	-1.2467916	-7.8031110	-0.0159797
H	1.0951424	-8.5786385	-0.3456087

B^{2+} : from dimeric Markovnikov addition
108
Energy = -3053.327788964

Ca	0.5632026	-0.5091667	1.6128547
Ca	-0.8862472	0.5561904	-1.6231650
H	-0.4944986	0.9612518	0.4214911
N	2.3798158	-0.2432722	3.4146586
N	0.0050779	0.1958369	-4.0555624
N	-1.6326629	-0.9112872	2.7217400
N	-2.8642169	2.1371077	-1.4782531
N	0.0878368	1.5948125	3.1728227
N	-0.0743931	2.7816045	-2.4867250
N	0.6776563	-2.8038905	3.1378616
N	-2.7919227	-0.4455985	-2.9760988
C	-2.1070142	0.4021029	3.2359369
C	-2.3146034	3.5115524	-1.6243433
C	2.6863433	-1.5849544	3.9745154
C	-0.9988699	-0.6763456	-4.7252994
C	-1.0382247	1.1443695	4.0289879
C	-1.2764149	3.6239471	-2.7336043
C	1.4159499	-2.3408524	4.3432393
C	-2.4423745	-0.3049287	-4.4160776
C	3.5814451	0.3010373	2.7431412
C	1.3371773	-0.4264397	-4.2593528
C	-2.5436411	-1.3674432	1.6501566
C	-3.5725988	2.0513447	-0.1830191
C	1.9740263	0.6680720	4.5141295
C	0.0139148	1.5554262	-4.6608145
C	-1.5842983	-1.9139520	3.8148657
C	-3.7994474	1.8088026	-2.5840737
C	1.2783459	1.9141765	3.9987586
C	0.6815966	2.5798480	-3.7522536
C	-0.7342859	-3.1296264	3.4718766
C	-4.0429174	0.3082015	-2.6867819
C	-0.3030501	2.8286670	2.4587087
C	0.7995621	3.4414559	-1.4893744
C	1.3215694	-4.0608173	2.6861171
C	-3.0041063	-1.8751815	-2.6533067
H	-3.0033323	0.2678506	3.8596782
H	-3.1223401	4.2343053	-1.8144272
H	3.3344981	-1.4934221	4.8591685
H	-0.8566459	-0.6535406	-5.8166366
H	-2.3948380	0.9948524	2.3645598
H	-1.8662953	3.7780881	-0.6628037
H	3.2489594	-2.1362606	3.2156493
H	-0.7943127	-1.7013118	-4.4011167
H	3.8823654	-0.3709819	1.9380127

H	1.3718645	-1.3922441	-3.7504412
H	-2.5019904	-0.6382075	0.8359331
H	-2.8546392	2.2284970	0.6193975
H	1.3115316	0.1222245	5.1889325
H	-1.0180539	1.8535359	-4.8605178
H	-1.1941798	-1.4203494	4.7074169
H	-3.3731054	2.1844280	-3.5175214
H	-0.6390528	0.4980307	4.8149869
H	-1.7135248	3.3230154	-3.6886619
H	0.7587158	-1.6970538	4.9312015
H	-2.6375278	0.7282910	-4.7120153
H	4.4170372	0.4139327	3.4500807
H	1.5340075	-0.5753618	-5.3309988
H	-3.5795656	-1.4524963	2.0096310
H	-4.3822041	2.7930300	-0.1195548
H	2.8553083	0.9610010	5.1051140
H	0.5298548	1.5365672	-5.6320761
H	-2.6007532	-2.2548886	4.0636463
H	-4.7607659	2.3252715	-2.4437562
H	-1.5004806	2.0058745	4.5354840
H	-0.9817120	4.6784573	-2.8403778
H	1.6679527	-3.1997663	4.9832105
H	-3.1064684	-0.9362359	-5.0250618
H	3.3574313	1.2805639	2.3134148
H	2.1185145	0.2110955	-3.8455979
H	-2.2220594	-2.3388830	1.2704115
H	-4.0050292	1.0570870	-0.0529394
H	-1.1739080	2.6328348	1.8354433
H	0.2515840	3.5835220	-0.5568922
H	2.3736384	-3.8815402	2.4625751
H	-2.1031176	-2.4469653	-2.8829007
H	1.9592811	2.4935774	3.3690196
H	1.6871113	2.2439534	-3.4814710
H	-1.1522423	-3.6512340	2.6050040
H	-4.4434575	-0.0746598	-1.7439259
H	1.0070909	2.5570422	4.8509087
H	0.7971130	3.5310301	-4.2923598
H	-0.7787154	-3.8343363	4.3174874
H	-4.8011192	0.1106430	-3.4585373
H	0.5151987	3.1345711	1.8035678
H	1.6641178	2.8050634	-1.2845230
H	0.8282805	-4.4350252	1.7880967
H	-3.2259995	-1.9785964	-1.5876386
H	-0.5243109	3.6400260	3.1698867
H	1.1522395	4.4173391	-1.8534282
H	1.2548646	-4.8290108	3.4713267
H	-3.8412783	-2.2942143	-3.2297624
C	0.4902670	-1.6654953	-0.8957602
C	-0.0514551	-3.0553949	-0.5885404
H	0.2679420	-1.4175269	-1.9474453
H	-0.0622226	-3.2605980	0.4902974
H	-1.0877860	-3.1546915	-0.9227933
C	1.9196817	-1.4548114	-0.6756197
C	2.7559736	-2.3990560	-0.0227251
C	2.5539351	-0.2391995	-1.0667442
C	4.1264326	-2.1802927	0.1368253
H	2.3360901	-3.3508622	0.2822026
C	3.9175850	-0.0276000	-0.9131382
H	1.9566218	0.5362101	-1.5468301
C	4.7267432	-1.0054922	-0.3186924
H	4.7340572	-2.9497161	0.6074870
H	4.3581545	0.9046421	-1.2573881
H	5.7932325	-0.8435532	-0.1999692
H	0.5231341	-3.8770170	-1.0501922

CH_2=CH_2 : ethylene substrate
6
Energy = -78.63740833188

C	-1.4501755	0.4945139	0.0000003
C	-0.1176829	0.4944971	0.0000082
H	-2.0220005	1.4196004	-0.0000137
H	-2.0220211	-0.4305622	-0.0000101
H	0.4541445	-0.4305917	-0.0000351
H	0.4541646	1.4195756	0.0000356

CH_2=CHBu : 1-hexene substrate
18
Energy = -235.9904743754

C	3.0775964	-0.0981642	-0.4543711
C	2.0354411	-0.2856764	0.3591128
H	3.0842558	0.7028831	-1.1918822
H	3.9582970	-0.7339727	-0.4121633
H	2.0682586	-1.1031350	1.0823091
C	0.7772023	0.5329051	0.3551433
C	-0.4729617	-0.3045008	0.0244202
H	0.8720775	1.3559226	-0.3644722
H	0.6352748	0.9855549	1.3479433
C	-1.7670800	0.5150377	0.0757769
H	-0.3554014	-0.7451775	-0.9747527
H	-0.5442341	-1.1435361	0.7306328
C	-3.0092301	-0.3180264	-0.2568930
H	-1.8746638	0.9543025	1.0769082
H	-1.6888235	1.3561852	-0.6264183
H	-3.9199914	0.2894259	-0.2136517
H	-2.9357068	-0.7444314	-1.2644410
H	-3.1236968	-1.1485795	0.4498899

CH_2=CHPh : conjugation-activated styrene
16
Energy = -309.8395678946

C	-2.9585407	0.3947222	0.0471828
C	-1.9583659	-0.4969394	-0.0081570
H	-2.7855462	1.4658418	0.1125006
H	-3.9943271	0.0700297	0.0283234
H	-2.2112625	-1.5554773	-0.0731301
C	-0.5168327	-0.2142229	0.0079064
C	0.3849503	-1.2906457	-0.0620449
C	0.0104475	1.0883741	0.0902702
C	1.7635202	-1.0794439	-0.0507017
H	-0.0071859	-2.3031868	-0.1261296

C	1.3854069	1.3004726	0.1016997
H	-0.6598573	1.9414275	0.1457786
C	2.2700354	0.2182340	0.0311560
H	2.4406537	-1.9273836	-0.1055418
H	1.7725874	2.3137537	0.1659827
H	3.3428690	0.3884393	0.0401809

CH_2=CHR : unactivated, R = 3-cyclohexenyl
20
Energy = -312.2314869812

C	-3.0931958	-0.0776241	-0.4271166
C	-2.0064624	-0.0058021	0.3449077
H	-3.0147417	-0.1368528	-1.5114303
H	-4.0954175	-0.0799271	-0.0062906
H	-2.1245912	0.0541057	1.4294510
C	-0.5904605	0.0000741	-0.1518199
C	0.1224975	1.3159544	0.2274255
C	0.2109176	-1.1938207	0.4078982
H	-0.6017368	-0.0800862	-1.2479343
C	1.6099883	1.2501196	-0.0017183
H	-0.0811777	1.5541118	1.2834163
H	-0.3058329	2.1443259	-0.3515397
C	1.6181697	-1.2450263	-0.2012486
H	0.2868175	-1.0867830	1.4986366
H	-0.3235624	-2.1293670	0.2095864
C	2.2759735	0.1097368	-0.2139780
H	2.1498798	2.1966119	-0.0006647
H	2.2442055	-1.9520965	0.3588964
H	1.5696586	-1.6397260	-1.2278084
H	3.3488985	0.1405997	-0.4008727

CH_3CH_2Bu : hexane product
20
Energy = -237.2242871135

C	-0.2276111	3.2158786	0.0001266
C	0.5418781	1.8907454	-0.0002351
H	-0.8692495	3.2948331	0.8860027
H	0.4532592	4.0743590	-0.0002730
H	-0.8702376	3.2948127	-0.8850399
H	1.1983779	1.8470603	-0.8801441
H	1.1989657	1.8469541	0.8792311
C	-0.3796112	0.6655071	-0.0000609
C	0.3795764	-0.6655059	0.0001416
H	-1.0375813	0.7078890	-0.8802460
H	-1.0376079	0.7080721	0.8800966
C	-0.5418894	-1.8907611	-0.0000818
H	1.0377823	-0.7080218	-0.8798602
H	1.0373394	-0.7079278	0.8804805
C	0.2276384	-3.2158732	0.0000474
H	-1.1988821	-1.8470904	0.8794641
H	-1.1984851	-1.8469895	-0.8799170
H	-0.4531953	-4.0743757	-0.0003510
H	0.8701443	-3.2946715	-0.8852170
H	0.8693888	-3.2948946	0.8858358

CH_3CH_2Ph : styrene hydroenation product
18
Energy = -311.0694391491

C	2.7427563	-0.0001108	0.7112006
C	1.9262363	-0.0000199	-0.5945934
H	2.5110003	-0.8861607	1.3120776
H	3.8172798	-0.0000053	0.4978622
H	2.5108711	0.8857808	1.3122660
H	2.1937851	0.8824722	-1.1875804
H	2.1937353	-0.8824459	-1.1877043
C	0.4372579	0.0000010	-0.3351106
C	-0.2667233	1.2032175	-0.1905550
C	-0.2666964	-1.2031772	-0.1900594
C	-1.6338300	1.2065711	0.0918721
H	0.2623072	2.1471630	-0.3052551
C	-1.6338104	-1.2064193	0.0923428
H	0.2623630	-2.1471645	-0.3042979
C	-2.3228071	0.0000990	0.2353763
H	-2.1620664	2.1507859	0.1957667
H	-2.1620527	-2.1505972	0.1965443
H	-3.3875366	0.0001192	0.4517923

CH_3CH_2R : alkane with R = 3-cyclohexenyl
22
Energy = -313.4648493572

C	-2.9760281	-0.4145775	-0.2849308
C	-1.9223168	0.5732448	0.2276842
H	-2.8066150	-1.4248680	0.1026831
H	-3.9823880	-0.1071073	0.0198044
H	-2.9574415	-0.4684241	-1.3801547
H	-2.1687540	1.5801988	-0.1364350
H	-1.9617888	0.6226983	1.3254424
C	-0.4846914	0.2391672	-0.1993015
C	0.4647961	1.4045175	0.1246209
C	0.0508633	-1.0469028	0.4481297
H	-0.4760424	0.0943618	-1.2913486
C	1.9163660	1.0394913	-0.0469113
H	0.2891973	1.7443948	1.1586254
H	0.2246770	2.2663208	-0.5133013
C	1.4335235	-1.4152654	-0.1075520
H	0.1257046	-0.8859927	1.5332891
H	-0.6440398	-1.8795656	0.2937801
C	2.3485933	-0.2204693	-0.1725590
H	2.6337308	1.8594441	-0.0770699
H	1.8945406	-2.1974220	0.5103746
H	1.3301963	-1.8539303	-1.1123284
H	3.4107853	-0.4129585	-0.3217775

CH_3CH_3 : ethane product
8
Energy = -79.87656483085

C	-1.0051159	-0.6409098	-0.0084068
C	0.5272731	-0.6408594	-0.0083934
H	-1.4008064	0.3809238	-0.0085347
H	-1.4007168	-1.1519760	-0.8932916

H	-1.4007267	-1.1517491	0.8766047
H	0.9229636	-1.6626930	-0.0084846
H	0.9228896	-0.1298188	-0.8932860
H	0.9228684	-0.1299943	0.8766103

C^{2+} : ethylene adduct of cation dimer **1^{2+}**
98
Energy = -2822.125662920

Ca	0.0558882	-0.6480947	1.9059034
Ca	0.2249039	-0.6522880	-1.6034287
H	-1.0655721	-0.1435179	0.0966104
N	0.9801353	-2.3138747	3.7363064
N	0.5682484	-2.4738353	-3.4339565
N	-1.0203321	1.4608878	2.9245798
N	-0.1221515	1.6844746	-2.5479881
N	1.8033917	0.5878339	3.2274060
N	2.2965306	0.0023265	-2.9309681
N	-1.8811054	-1.4402103	3.3189401
N	-1.8649614	-0.7775021	-2.9928674
C	0.0513329	2.3656767	3.4131265
C	1.1572294	2.2194421	-3.0808872
C	-0.1983906	-3.0011279	4.3299262
C	-0.7530257	-2.7423779	-4.0652664
C	1.1849911	1.6175277	4.1017252
C	1.9688213	1.1560720	-3.8078478
C	-1.3897878	-2.0830856	4.5648508
C	-1.5868465	-1.4826881	-4.2679987
C	1.9570671	-3.3603485	3.3531218
C	1.1446008	-3.7674173	-2.9965874
C	-1.8838251	2.2102378	1.9853755
C	-0.6397723	2.6067663	-1.5156876
C	1.6218343	-1.4028041	4.7208613
C	1.4917095	-1.8424815	-4.4152436
C	-1.8420639	0.9534719	4.0525136
C	-1.1247855	1.5315967	-3.6353319
C	2.5534382	-0.3985890	4.0495434
C	2.6897741	-1.1741674	-3.7496963
C	-2.6988904	-0.2423224	3.6516446
C	-2.2875114	0.6252870	-3.2412147
C	2.7070285	1.2191734	2.2402714
C	3.4012037	0.3606615	-2.0131405
C	-2.7085909	-2.3910710	2.5407988
C	-2.9226103	-1.4798364	-2.2312137
H	-0.3524342	3.1205327	4.1054924
H	0.9784777	3.0679848	-3.7582909
H	0.0722957	-3.4792041	5.2844676
H	-0.6237038	-3.2467470	-5.0351814
H	0.4337787	2.9074125	2.5429592
H	1.7241801	2.6077181	-2.2298839
H	-0.4740670	-3.8052849	3.6404369
H	-1.2828524	-3.4416898	-3.4121131
H	1.5143944	-4.0166445	2.6026474
H	0.4623659	-4.2501147	-2.2955989
H	-1.2628390	2.6277695	1.1929854
H	0.1037052	2.7004870	-0.7193145
H	0.8370754	-0.8714073	5.2651622
H	0.9275759	-1.1061302	-4.9929005
H	-1.1712175	0.6747280	4.8695926
H	-0.6142358	1.1271662	-4.5133180
H	0.8134638	1.1176076	4.9992631
H	1.4071786	0.7823847	-4.6672545
H	-1.1221547	-1.2932955	5.2706189
H	-1.0656237	-0.7902379	-4.9336268
H	2.2451398	-3.9606660	4.2291984
H	1.3040578	-4.4352361	-3.8562545
H	-2.4092565	3.0291858	2.5003717
H	-0.8408186	3.6049043	-1.9333265
H	2.1857885	-1.9839015	5.4661652
H	1.8513362	-2.5965377	-5.1315602
H	-2.4951522	1.7524095	4.4365523
H	-1.5213057	2.5165950	-3.9256365
H	1.9434943	2.3418190	4.4346140
H	2.8896943	1.6101269	-4.2041545
H	-2.1964092	-2.6643642	5.0366556
H	-2.5271502	-1.7556864	-4.7711400
H	2.8541772	-2.9043462	2.9324623
H	2.1008440	-3.6040213	-2.4960008
H	-2.6110708	1.5402328	1.5258077
H	-1.5540331	2.1995748	-1.0834769
H	2.1491892	1.9408282	1.6365201
H	3.1090096	1.2121619	-1.3932310
H	-2.1409712	-3.3023679	2.3406285
H	-2.6255374	-2.5159896	-2.0511279
H	3.2509919	-0.9167334	3.3860050
H	3.1938856	-1.8808613	-3.0840779
H	-3.2940076	0.0015292	2.7679252
H	-2.7489069	0.9875079	-2.3183717
H	3.1561803	0.1130071	4.8142423
H	3.4182918	-0.8842468	-4.5219314
H	-3.4052818	-0.4730772	4.4629996
H	-3.0574357	0.6669070	-4.0266395
H	3.1108130	0.4476183	1.5794834
H	3.6164064	-0.4874332	-1.3596358
H	-2.9803683	-1.9295637	1.5873718
H	-3.0464666	-0.9818244	-1.2666729
H	3.5376920	1.7480184	2.7295961
H	4.3119599	0.6305191	-2.5677917
H	-3.6239136	-2.6631102	3.0865965
H	-3.8767551	-1.4808800	-2.7791976
C	1.4567806	-1.9262742	0.1928562
C	0.4391538	-3.0868305	0.2884666
H	2.1961655	-2.0405925	1.0019448
H	2.0731975	-2.0869172	-0.7122412
H	-0.1202556	-3.0764863	1.2404641
H	-0.3454695	-3.0323748	-0.4864393
H	0.8616610	-4.1000166	0.2196327

H_2 : dihydrogen
2
Energy = -1.180087137796

H	0.0000000	0.0000000	0.0279143
H	0.0000000	0.0000000	0.7720857

mA0a$^+$.H_2 : loose complex of H_2

68

Energy = -1685.146809031

Ca	-0.3244332	-0.2184156	-0.5006997
N	-0.5983317	-0.9906960	1.8927711
N	-0.0591846	1.8445758	0.9519422
N	-2.0403250	1.4929082	-1.3309279
N	-2.5485868	-1.3416415	-0.3804930
C	-0.8521251	0.2283120	2.7049131
H	-0.7524727	-0.0015048	3.7763645
H	-1.8892245	0.5346926	2.5462138
C	0.0932858	1.3723409	2.3546730
H	-0.0677204	2.2008628	3.0612155
H	1.1324300	1.0502552	2.4711383
C	-1.3182211	2.6197720	0.7969235
H	-1.2080308	3.6155313	1.2526386
H	-2.1073820	2.1065396	1.3516394
C	-1.7307853	2.7861035	-0.6604635
H	-2.5966910	3.4632504	-0.7130424
H	-0.9243292	3.2584228	-1.2287405
C	-3.3608293	0.9756389	-0.8775948
H	-4.1739424	1.5218602	-1.3785041
H	-3.4586056	1.1801611	0.1913512
C	-3.5274603	-0.5179370	-1.1386331
H	-4.5589472	-0.8142796	-0.8953544
H	-3.3745683	-0.7322147	-2.2003369
C	-2.9248621	-1.4264679	1.0541522
H	-3.7798370	-2.1071249	1.1852511
H	-3.2572344	-0.4366220	1.3774850
C	-1.7734901	-1.9034600	1.9328997
H	-2.1350785	-2.0243473	2.9651180
H	-1.4282409	-2.8859084	1.5997705
C	0.5804926	-1.7113074	2.4289451
H	1.4467450	-1.0478747	2.4451418
H	0.8073622	-2.5628834	1.7843943
H	0.3918008	-2.0689666	3.4518075
C	1.0993700	2.7021902	0.6091488
H	1.0190957	3.0445206	-0.4250145
H	2.0224522	2.1274727	0.7123797
H	1.1467404	3.5833637	1.2660902
C	-2.0713551	1.7246530	-2.7945551
H	-2.3792244	0.8139776	-3.3109608
H	-1.0722543	2.0025191	-3.1404437
H	-2.7752339	2.5300779	-3.0513741
C	-2.4747145	-2.6980203	-0.9717873
H	-1.7163217	-3.2903935	-0.4556429
H	-2.1921097	-2.6166980	-2.0232900
H	-3.4411810	-3.2176043	-0.8977542
C	1.4989847	-1.6281072	-1.2258732
C	2.0565731	-2.2767151	-2.4942694
H	1.2728439	-2.4476161	-0.5088700
H	3.0381505	-2.7728160	-2.3724234
H	1.3617884	-3.0446477	-2.8595832
H	2.1730399	-1.5540863	-3.3144869
C	2.4884331	-0.6934446	-0.5122884
C	3.6893673	-1.3651858	0.1874111
C	3.0122669	0.4241844	-1.4326356
H	1.9609811	-0.1639776	0.3261685
C	4.4608727	-0.3696295	1.0647835
H	4.3582307	-1.7709667	-0.5826164
H	3.3378136	-2.2128202	0.7871582
C	4.0665294	1.2777506	-0.7796727
H	3.4158825	-0.0211664	-2.3522770
H	2.1805547	1.0631781	-1.7742736
C	4.7016059	0.9322294	0.3467550
H	5.4202986	-0.7949149	1.3882977
H	3.9001862	-0.1749691	1.9943747
H	4.3095602	2.2238590	-1.2628297
H	5.4407570	1.6092083	0.7737670
H	-0.2116343	-1.0755463	-2.7608829
H	-0.8296640	-0.8129126	-3.1234180

mA0$^+$.H_2 : loose complex of H_2

68

Energy = -1685.148866390

C	-0.5082905	-6.2813671	-0.3511251
C	-0.4982802	-5.8467632	1.0912198
C	-0.4352133	-4.3188703	1.2214687
C	0.6080006	-3.7127335	0.2720394
C	0.2234192	-4.0303111	-1.1791807
C	-0.2055189	-5.4627532	-1.3652950
C	0.7867032	-2.1967848	0.5121560
C	1.8277642	-1.4188010	-0.3091971
Ca	0.6032929	0.6403983	-0.0389562
N	-1.0234357	1.0411595	1.8353786
C	-0.7945321	0.1729148	3.0140785
C	-2.3883339	0.7872404	1.2980421
C	-2.5810492	1.3281019	-0.1156874
N	-1.6853227	0.6757023	-1.1086670
C	-2.2148678	-0.6611483	-1.4667884
C	-1.5886893	1.4959188	-2.3480210
C	-0.7042096	2.7270936	-2.1813934
N	0.6964150	2.3833307	-1.8241653
C	1.4235447	1.8630450	-3.0050856
C	1.4066357	3.5718447	-1.2802280
C	1.0120808	3.8730340	0.1624661
N	1.3518302	2.7602044	1.0886204
C	2.8055149	2.7592528	1.3733716
C	-0.8671399	2.4701585	2.2192460
C	0.5941428	2.8852837	2.3631702
H	-0.7282330	3.3111995	-3.1138340
H	-1.1040979	3.3724436	-1.3950318
H	-2.5881390	1.8148638	-2.6795167
H	-1.1865921	0.8474423	-3.1313785
H	-3.6331911	1.1937000	-0.4080991
H	-2.3848855	2.4031169	-0.1360272
H	-3.1529790	1.2255127	1.9567145

H	-2.5405638	-0.2958386	1.3090661
H	-1.3905928	2.6675526	3.1666897
H	-1.3552355	3.0823689	1.4568648
H	0.6392053	3.9161017	2.7448248
H	1.0942390	2.2511764	3.1005128
H	1.5073931	4.8010910	0.4852105
H	-0.0644115	4.0514267	0.2257800
H	1.2149148	4.4595408	-1.9008491
H	2.4787599	3.3657587	-1.3419544
H	0.9173654	0.9775034	-3.3958202
H	2.4359671	1.5789488	-2.7073021
H	1.4813092	2.6162942	-3.8041065
H	-2.3274540	-1.2720717	-0.5693271
H	-1.5125049	-1.1573639	-2.1408645
H	-3.1928089	-0.5807521	-1.9633723
H	0.2178198	0.3196187	3.3971213
H	-0.9052466	-0.8721061	2.7170394
H	-1.5103318	0.3968003	3.8183244
H	3.3685606	2.6334002	0.4458534
H	3.0431836	1.9264714	2.0384206
H	3.1189096	3.6992457	1.8503053
H	2.8368659	-1.7219820	0.0048329
H	1.7398749	-1.6979589	-1.3761318
H	1.0008945	-2.0673864	1.5849078
H	-0.2373066	-1.7662060	0.3771686
H	1.5773716	-4.1923483	0.4764658
H	-1.4209867	-3.8942768	0.9785984
H	-0.2115117	-4.0366045	2.2583097
H	-0.5925204	-3.3595915	-1.4977518
H	1.0637046	-3.8063307	-1.8477509
H	-1.3894055	-6.2390133	1.5997163
H	0.3615211	-6.3030060	1.6056980
H	-0.2556844	-5.8360195	-2.3881306
H	-0.7831605	-7.3152854	-0.5581971
H	2.8930632	-0.6530052	2.1830853
H	2.9774401	-0.5475659	2.9174976

mA0$^+$: anti-Markovnikov adduct of **1m**$^+$
66
Energy = -1683.970812996

Ca	0.4745191	0.3575634	-0.5942087
N	2.5854536	-0.9110019	-1.0171862
N	2.3289923	1.9978970	-0.3174033
N	0.1875614	1.3481142	1.7083687
N	0.4484901	-1.5971651	0.9996799
C	3.7049760	-0.0900575	-0.4850085
H	4.6677060	-0.4688990	-0.8596458
H	3.7214529	-0.2059549	0.6018861
C	3.5750157	1.3857294	-0.8514947
H	4.4623951	1.9265892	-0.4906512
H	3.5517455	1.5018912	-1.9385557
C	2.4546320	2.2725020	1.1373001
H	3.0992715	3.1482077	1.3068080
H	2.9554684	1.4179714	1.6003042
C	1.1062489	2.5160408	1.8088511
H	1.2739348	2.7869888	2.8617395
H	0.6004663	3.3643622	1.3394868
C	0.5899681	0.2809857	2.6645879
H	0.2855601	0.5535794	3.6860496
H	1.6813480	0.2224340	2.6679638
C	-0.0054852	-1.0812725	2.3192946
H	0.2418330	-1.7926486	3.1216050
H	-1.0971207	-1.0156036	2.2797978
C	1.8559350	-2.0737898	1.0748551
H	1.9008464	-3.0431288	1.5935156
H	2.4248765	-1.3644597	1.6811368
C	2.4997644	-2.2123541	-0.3016852
H	3.4961656	-2.6649573	-0.1915389
H	1.9101983	-2.8890524	-0.9263690
C	2.7545569	-1.1431932	-2.4699213
H	2.7838541	-0.1884304	-2.9999562
H	1.9045147	-1.7196728	-2.8438040
H	3.6823265	-1.6933123	-2.6835027
C	2.0265596	3.2434211	-1.0598372
H	1.0940515	3.6799942	-0.6951930
H	1.9051727	3.0086824	-2.1198867
H	2.8308859	3.9840418	-0.9433824
C	-1.1938060	1.7960702	2.0020198
H	-1.8779924	0.9472015	1.9552400
H	-1.5021282	2.5331142	1.2567850
H	-1.2617825	2.2469024	3.0025577
C	-0.4389673	-2.7054018	0.5772804
H	-0.1413666	-3.0674790	-0.4095686
H	-1.4698688	-2.3481376	0.5193000
H	-0.3916468	-3.5446625	1.2866470
C	-1.0532658	0.8902216	-2.4348193
C	-2.4309032	1.1792495	-1.7990802
H	-1.1684058	0.1150398	-3.2132348
H	-0.7264370	1.7936297	-2.9840337
H	-2.3735512	2.1000551	-1.1947270
H	-3.2422336	1.3644551	-2.5303805
C	-2.9227168	0.0508354	-0.8769880
C	-4.2040684	0.4050223	-0.1110845
C	-3.1263019	-1.2653893	-1.6409945
H	-2.1431276	-0.1425516	-0.1096573
C	-4.5511521	-0.6725797	0.9246782
H	-5.0276627	0.4958232	-0.8319178
H	-4.0967716	1.3821126	0.3757538
C	-3.8069504	-2.3194322	-0.8083755
H	-3.7153836	-1.0645431	-2.5500801
H	-2.1598411	-1.6438064	-1.9998893
C	-4.4223496	-2.0599014	0.3515737
H	-5.5702060	-0.5253968	1.3051954
H	-3.8959981	-0.5781290	1.8066061
H	-3.7760071	-3.3433180	-1.1801180
H	-4.8695750	-2.8760928	0.9177880

mAa0$^+$: Markovnikov adduct of **1m**$^+$
66
Energy = -1683.964304381

Ca	16.9302113	11.0652751	14.9570364
N	17.3603175	13.2031500	13.7223122
N	15.3144916	11.0738504	13.0039479
N	14.6505489	10.7668704	15.9459683
N	16.6676157	12.8829263	16.6377545
C	16.1782270	13.4336816	12.8510704
H	16.3942972	14.2260854	12.1186185
H	15.3621601	13.8011421	13.4786822
C	15.7393412	12.1788502	12.1022998
H	14.9290853	12.4445584	11.4063432
H	16.5656361	11.7946449	11.4971386
C	13.9850371	11.3630429	13.6058176
H	13.1890988	11.2055830	12.8623902
H	13.9560302	12.4207960	13.8791142
C	13.6984008	10.5026530	14.8336048
H	12.6611684	10.6708625	15.1591776
H	13.7834902	9.4426585	14.5778336
C	14.3094033	12.0366219	16.6405903
H	13.4385966	11.8900215	17.2972205
H	14.0139196	12.7667838	15.8827344
C	15.4700043	12.5838504	17.4668458
H	15.1347147	13.4797065	18.0104871
H	15.7734229	11.8505470	18.2193082
C	16.4918692	14.1488029	15.8815568
H	16.5978876	15.0148620	16.5525833
H	15.4691708	14.1718361	15.4953320
C	17.4879489	14.2886007	14.7331322
H	17.3576166	15.2745849	14.2628514
H	18.5109759	14.2523321	15.1172516
C	18.5965032	13.1474967	12.9077128
H	18.5090605	12.3642962	12.1519188
H	19.4444045	12.9110756	13.5550682
H	18.7848188	14.1055232	12.4016206
C	15.2484878	9.8191415	12.2192382
H	14.9357406	8.9927289	12.8610154
H	16.2377401	9.5881479	11.8169178
H	14.5313356	9.9082524	11.3897470
C	14.6400449	9.6335495	16.8983821
H	15.3578663	9.8140865	17.7019958
H	14.9290801	8.7187793	16.3738020
H	13.6445719	9.4891760	17.3425967
C	17.8695351	12.9490252	17.5005501
H	18.7562757	13.1391926	16.8925793
H	18.0007566	11.9891186	18.0057271
H	17.7784376	13.7454930	18.2534777
C	18.9705439	9.8405795	15.1950073
C	19.5646262	8.7871671	16.1325996
H	19.6644559	10.7108559	15.1878489
H	20.5175884	8.3370666	15.7897211
H	19.7651538	9.2331842	17.1152602
H	18.8731700	7.9522533	16.3103356
C	18.8498083	9.3880979	13.7293217
C	20.1577400	9.3096283	12.9138129
C	18.0940649	8.0555666	13.5807077
H	18.2364298	10.1369826	13.1544196
C	19.8800044	9.1013500	11.4177945
H	20.7577360	8.4742928	13.2979505
H	20.7406247	10.2250848	13.0701102
C	18.0411823	7.5688858	12.1570401
H	18.5720690	7.2955004	14.2139394
H	17.0698792	8.1465125	13.9801210
C	18.8280790	8.0483627	11.1862163
H	20.7993562	8.8268266	10.8832400
H	19.5508329	10.0502442	10.9625881
H	17.3190886	6.7866620	11.9237820
H	18.7236229	7.6621462	10.1727958

mAa$^+$: anti-Markovnikov adduct of **1m**$^+$**.**THF

79

Energy = -1916.574746368

Ca	0.2534656	0.3902279	0.2447501
N	0.9352243	0.2366894	2.7245187
N	-1.0861521	2.7010684	-0.2354344
N	1.7192603	2.3913100	0.7328950
C	-0.2594919	-0.0671727	3.5486490
C	1.9450287	-0.8221517	2.9365703
C	1.5175162	1.5529842	3.0828049
C	-0.1060031	3.8150658	-0.2116818
C	-1.8510835	2.7890468	-1.4971606
C	-2.0271745	2.8121640	0.9062166
C	0.9518753	3.6520079	0.8706677
C	2.4439555	2.0875388	1.9942588
C	2.6853861	2.4977888	-0.3835550
C	-1.4842955	0.7209590	3.1072615
H	-0.0681714	0.1319640	4.6148788
H	-0.4489678	-1.1403889	3.4551141
H	1.5224793	-1.7848176	2.6457179
H	2.2556533	-0.8630371	3.9926820
H	2.8148913	-0.6351304	2.3075537
H	0.7001916	2.2575346	3.2580361
H	2.0790338	1.4783682	4.0273345
H	-0.6133606	4.7831065	-0.0710016
H	0.3667787	3.8477261	-1.1975459
H	-1.1698613	2.6916665	-2.3438378
H	-2.3767098	3.7540373	-1.5721823
H	-2.5865109	1.9858343	-1.5489686
C	-2.7660984	1.5096265	1.1919716
H	-1.4629940	3.1223642	1.7888871
H	-2.7669832	3.6053915	0.7133031
H	0.4817645	3.6566481	1.8568544
H	1.6309396	4.5184506	0.8400698
H	3.2077587	1.3420845	1.7561610
H	2.9671416	2.9821073	2.3657833
H	2.1588322	2.7417296	-1.3096709
H	3.1875101	1.5362654	-0.5120808
H	3.4344040	3.2813137	-0.1943340
N	-1.8730342	0.4347847	1.7006271
H	-1.2856451	1.7913643	3.1950847
H	-2.3236086	0.4981565	3.7839596
H	-3.2356913	1.1362327	0.2770088

H	-3.5772706	1.7053660	1.9104359
C	-2.5945451	-0.8568994	1.6402036
H	-1.9520848	-1.6626453	2.0009029
H	-2.8738805	-1.0690267	0.6053169
H	-3.5079807	-0.8322044	2.2536683
H	-2.8223248	-0.2105781	-2.1055024
C	-1.9437303	-0.8367039	-2.2986496
C	-1.7936311	-1.1684317	-3.7807256
H	-1.9765164	-1.7209939	-1.6577200
O	-0.7557568	-0.0613882	-1.9410059
C	-0.9516796	0.0071467	-4.2993692
H	-2.7616776	-1.2569510	-4.2785846
H	-1.2489668	-2.1094502	-3.9061995
C	0.0199390	0.2183478	-3.1486158
H	-1.5761403	0.8949358	-4.4445317
H	-0.4363917	-0.2163659	-5.2361758
H	0.8621457	-0.4815948	-3.1869556
H	0.4022600	1.2397309	-3.0651383
H	1.6668927	-1.3476741	-1.5063664
C	1.6807779	-1.5119730	-0.4089694
C	0.5394097	-2.5003000	-0.0889794
H	-0.4673163	-2.0331874	-0.0893495
C	3.0375032	-2.1536278	-0.1028670
H	0.4286224	-3.3615591	-0.7732811
H	0.6683427	-2.9226343	0.9192213
C	3.3502808	-3.4025909	-0.9628620
C	4.2023133	-1.1654331	-0.2462250
H	3.0343214	-2.4953071	0.9466559
C	4.7711764	-3.8883304	-0.8339150
H	3.1333378	-3.1658455	-2.0184153
H	2.6640953	-4.2192345	-0.7007324
C	5.5404432	-1.7548692	0.2216290
H	4.2814976	-0.8625382	-1.3009544
H	3.9742226	-0.2567323	0.3252234
C	5.7498165	-3.1572139	-0.2875999
H	4.9971528	-4.8906674	-1.2011308
H	6.3763022	-1.1183150	-0.1010172
H	5.5822077	-1.7588098	1.3228544
H	6.7527009	-3.5754395	-0.2001797

mA$^+$: Markovnikov adduct of **1m**$^+$.THF
79
Energy = -1916.581981959

Ca	0.6231377	-0.2473576	-0.6427534
N	-0.0384741	-2.5147571	-1.5072869
N	1.6847455	-0.6259500	-3.0187300
N	3.1804888	-0.0127143	-0.4018106
N	1.4685603	-1.9536047	1.0864489
C	0.8446758	-2.9498196	-2.6140907
H	0.4182045	-3.8239301	-3.1310286
H	1.7972109	-3.2712815	-2.1852275
C	1.0789429	-1.8377223	-3.6300630
H	1.7086269	-2.2212993	-4.4479992
H	0.1289195	-1.5331536	-4.0777007
C	3.1325162	-0.8477640	-2.7702711
H	3.6915740	-0.7764958	-3.7165130
H	3.2635881	-1.8702281	-2.4079567
C	3.7299595	0.1404460	-1.7742409
H	4.8249075	0.0225967	-1.7683453
H	3.5196193	1.1654359	-2.0931622
C	3.7057823	-1.2456612	0.2378080
H	4.7530035	-1.0999285	0.5453526
H	3.7039026	-2.0446022	-0.5072557
C	2.8796316	-1.6672605	1.4455664
H	3.3525494	-2.5405037	1.9211073
H	2.8685571	-0.8677458	2.1918565
C	1.3520326	-3.2822567	0.4326170
H	1.4141813	-4.0819163	1.1871736
H	2.2090002	-3.4127098	-0.2336168
C	0.0517761	-3.4402516	-0.3495307
H	-0.0468348	-4.4866109	-0.6783390
H	-0.8006498	-3.2204903	0.2989192
C	-1.4441214	-2.4400604	-1.9650373
H	-1.5283332	-1.7412260	-2.8010471
H	-2.0624844	-2.0725505	-1.1441871
H	-1.8080042	-3.4245758	-2.2965771
C	1.5152687	0.5139639	-3.9465104
H	1.9134009	1.4244839	-3.4959299
H	0.4514555	0.6611476	-4.1447215
H	2.0310991	0.3291881	-4.9012837
C	3.5563877	1.1678132	0.4057575
H	3.1044912	1.1053220	1.3988750
H	3.1955158	2.0718688	-0.0880531
H	4.6479689	1.2427980	0.5242217
C	0.6399869	-1.9260460	2.3124648
H	-0.4065823	-2.0922853	2.0569086
H	0.7215874	-0.9417008	2.7777813
H	0.9714607	-2.6925658	3.0300503
C	-0.8313523	2.3111653	-2.0965440
O	0.3849536	2.0760660	-1.3198352
C	0.6338110	3.2433990	-0.4671693
C	-0.5317086	4.2090908	-0.7043639
C	-1.6354192	3.3027229	-1.2736261
H	-0.5481861	2.7181889	-3.0741794
H	-1.3286070	1.3452265	-2.2257884
H	0.6747834	2.8885525	0.5672853
H	1.6014008	3.6678983	-0.7477228
H	-0.8305948	4.7128867	0.2176888
H	-0.2505859	4.9712766	-1.4370361
H	-2.1624920	2.7766042	-0.4734298
H	-2.3608339	3.8507261	-1.8795256
C	-1.5642913	0.0038071	0.5219113
C	-1.5961474	0.9632493	1.7223764
H	-1.9509520	-0.9807013	0.8530288
H	-2.3266705	0.3536545	-0.2062009
H	-0.9093357	0.6115967	2.5120539
H	-1.2027198	1.9495953	1.4250211
C	-2.9608534	1.2210816	2.4009471
C	-3.5188425	-0.0272680	3.0924332
C	-2.8794761	2.3717285	3.4169527

H	-3.6735712	1.5139582	1.6128215
C	-4.9359952	0.2127311	3.6297572
H	-2.8530406	-0.2898653	3.9286413
H	-3.5130131	-0.8729649	2.3954924
C	-4.1141280	2.4886452	4.2732415
H	-1.9954142	2.2282273	4.0612211
H	-2.7061784	3.3216645	2.8905779
C	-5.0454953	1.5306090	4.3526079
H	-5.2338369	-0.6010804	4.3051534
H	-5.6610714	0.1892818	2.8008370
H	-4.2439535	3.4100995	4.8416144
H	-5.9294153	1.6901062	4.9701161

mBa^{+}.H_2 : loose H_2 complex
64
Energy = -1682.760594319

Ca	-0.3161290	-0.1300595	-0.5052803
N	-2.6793635	0.5437216	-0.9610316
N	-1.7356570	-2.0105852	0.3059340
N	0.2763946	-0.5047105	1.9279427
N	-0.6596571	2.0964609	0.6399310
C	-3.5530393	-0.3539306	-0.1609368
H	-4.5952918	-0.2788058	-0.5060880
H	-3.5374557	-0.0064555	0.8756156
C	-3.1084310	-1.8120912	-0.2305884
H	-3.8340375	-2.4381458	0.3094623
H	-3.1026035	-2.1533980	-1.2692432
C	-1.7406284	-1.9881347	1.7907992
H	-2.1587842	-2.9256614	2.1875836
H	-2.4070757	-1.1836990	2.1131984
C	-0.3474083	-1.7791161	2.3752125
H	-0.4070940	-1.8211157	3.4730762
H	0.3163564	-2.5897593	2.0625836
C	-0.3093500	0.6468918	2.6637033
H	0.0887186	0.6807684	3.6891519
H	-1.3864462	0.4824161	2.7505463
C	-0.0369599	1.9874223	1.9878499
H	-0.3898221	2.7970881	2.6441636
H	1.0398561	2.1287484	1.8560552
C	-2.1320652	2.2810798	0.7516276
H	-2.3638762	3.3136944	1.0523311
H	-2.4974275	1.6302404	1.5497900
C	-2.8593848	1.9612866	-0.5505275
H	-3.9260916	2.2059754	-0.4396618
H	-2.4727983	2.5857386	-1.3605780
C	-2.9735750	0.3960886	-2.4056599
H	-2.7945051	-0.6340343	-2.7197137
H	-2.3088132	1.0473970	-2.9771781
H	-4.0172813	0.6613007	-2.6287507
C	-1.1806281	-3.2877911	-0.1983598
H	-0.1623535	-3.4259182	0.1735689
H	-1.1484445	-3.2569721	-1.2898055
H	-1.7892325	-4.1450879	0.1238687
C	1.7373810	-0.5666009	2.1602147
H	2.2184126	0.3378210	1.7830648
H	2.1512314	-1.4192034	1.6190248
H	1.9664686	-0.6738889	3.2308246
C	-0.0606347	3.2435736	-0.0793469
H	-0.4918725	3.3263666	-1.0788122
H	1.0154762	3.0852120	-0.1745338
H	-0.2360105	4.1869282	0.4578272
C	0.7107250	-0.8803691	-2.6222716
C	2.0830290	-1.4865055	-2.2631718
H	0.8523200	-0.0950659	-3.3866876
H	0.1170908	-1.6610407	-3.1315088
H	1.9442681	-2.4474961	-1.7436851
H	2.7091513	-1.7381301	-3.1412653
C	2.9400080	-0.6106942	-1.3617228
C	3.9661743	-1.1650338	-0.5796645
C	2.7430107	0.7777334	-1.2914074
C	4.7591837	-0.3695967	0.2464800
H	4.1389029	-2.2388840	-0.6192871
C	3.5400398	1.5832332	-0.4681537
H	1.9804333	1.2312579	-1.9221177
C	4.5474379	1.0117423	0.3084097
H	5.5407966	-0.8259912	0.8480281
H	3.3841582	2.6589161	-0.4456988
H	5.1648996	1.6331810	0.9505239
H	0.0696329	2.0864261	-3.5728129
H	-0.0775154	2.8127815	-3.6514943

mBa^{+} : anti-Markovnikov styrene adduct
62
Energy = -1681.579240313

Ca	-0.3192132	-0.1114601	-0.5422396
N	-2.6792653	0.5595753	-1.0069551
N	-1.7406974	-1.9821147	0.2843340
N	0.2713980	-0.4631084	1.8962715
N	-0.6618597	2.1210348	0.5789449
C	-3.5594505	-0.3328768	-0.2089505
H	-4.5983572	-0.2639032	-0.5654602
H	-3.5555774	0.0242313	0.8244890
C	-3.1111549	-1.7908573	-0.2607271
H	-3.8385266	-2.4127140	0.2819163
H	-3.0992260	-2.1427359	-1.2958192
C	-1.7506817	-1.9412241	1.7686596
H	-2.1735213	-2.8722512	2.1758239
H	-2.4154442	-1.1303845	2.0784844
C	-0.3587911	-1.7297702	2.3560607
H	-0.4230763	-1.7587693	3.4540922
H	0.3032616	-2.5465063	2.0559235
C	-0.3080092	0.6992342	2.6201655
H	0.0943832	0.7449994	3.6434746
H	-1.3851405	0.5384722	2.7134176
C	-0.0353099	2.0308276	1.9259978
H	-0.3846431	2.8495506	2.5729342
H	1.0413908	2.1680976	1.7890518
C	-2.1332409	2.3098482	0.6910226
H	-2.3626917	3.3455839	0.9830749
H	-2.4987935	1.6661323	1.4950070

C	-2.8622160	1.9797184	-0.6076167
H	-3.9293098	2.2226486	-0.4966631
H	-2.4783034	2.5990322	-1.4228587
C	-2.9548933	0.4033740	-2.4541655
H	-2.7880786	-0.6324776	-2.7557536
H	-2.2690122	1.0375964	-3.0208765
H	-3.9906143	0.6836460	-2.6955750
C	-1.1823643	-3.2640052	-0.2037592
H	-0.1654046	-3.3973072	0.1735922
H	-1.1459804	-3.2446310	-1.2955010
H	-1.7911938	-4.1188002	0.1247120
C	1.7323527	-0.5298926	2.1263177
H	2.2155076	0.3721053	1.7463075
H	2.1426957	-1.3851402	1.5862072
H	1.9633885	-0.6354581	3.1967140
C	-0.0612761	3.2494930	-0.1676142
H	-0.4788027	3.2976872	-1.1759142
H	1.0168426	3.0951242	-0.2433777
H	-0.2495454	4.2084783	0.3366709
C	0.6817769	-0.8075220	-2.6852455
C	2.0674834	-1.4119611	-2.3790762
H	0.7970351	0.0258606	-3.4029380
H	0.0906845	-1.5665964	-3.2284977
H	1.9488917	-2.3995004	-1.9070810
H	2.6847527	-1.6084908	-3.2777161
C	2.9241899	-0.5695510	-1.4464706
C	3.9402491	-1.1523468	-0.6721147
C	2.7379148	0.8185959	-1.3416252
C	4.7376120	-0.3842505	0.1759587
H	4.1031633	-2.2265022	-0.7370008
C	3.5400733	1.5964425	-0.4972752
H	1.9814942	1.2952985	-1.9626537
C	4.5400416	0.9972549	0.2683638
H	5.5118213	-0.8624657	0.7701208
H	3.3937883	2.6727324	-0.4496259
H	5.1615841	1.5975929	0.9264530

mB^{+}**.**H_2 : loose H_2 complex

64

Energy = -1682.776481696

Ca	0.0267551	-0.3131181	-0.1125533
N	0.9327197	1.5840593	-1.6012696
N	-0.2760034	1.8079989	1.2143472
N	1.2769338	-0.6284917	2.0894823
N	2.4136049	-0.8707714	-0.7038973
C	1.1755943	2.6883414	-0.6340860
H	1.4183146	3.6159370	-1.1740196
H	2.0542872	2.4349361	-0.0354984
C	-0.0192507	2.9375809	0.2786023
H	0.1413088	3.8699367	0.8399490
H	-0.9264350	3.0747387	-0.3161068
C	0.7217414	1.8137269	2.3187381
H	0.4528318	2.5720701	3.0687005
H	1.6869650	2.1144604	1.9044283
C	0.8488393	0.4605697	3.0110550
H	1.5508651	0.5548001	3.8533202
H	-0.1161624	0.1642675	3.4330595
C	2.7163867	-0.4862060	1.7412836
H	3.3426349	-0.7706612	2.6000674
H	2.9189392	0.5685661	1.5382067
C	3.1011475	-1.3303361	0.5325261
H	4.1928274	-1.3059131	0.4012455
H	2.8222507	-2.3743536	0.6951550
C	3.0876268	0.3428693	-1.2353501
H	4.0224135	0.0641149	-1.7443445
H	3.3687054	0.9733995	-0.3885114
C	2.2128684	1.1233546	-2.2052338
H	2.7859972	1.9790451	-2.5940025
H	1.9523442	0.4980503	-3.0641712
C	0.0720857	2.0862907	-2.6986642
H	-0.8837527	2.4267952	-2.3005374
H	-0.1120673	1.2816380	-3.4130499
H	0.5588474	2.9223095	-3.2222693
C	-1.6469942	1.9534923	1.7629911
H	-1.8183576	1.2043492	2.5366008
H	-2.3771680	1.8023554	0.9621969
H	-1.7885593	2.9516759	2.2017770
C	1.0724267	-1.9374966	2.7573291
H	1.2945079	-2.7519373	2.0655124
H	0.0325445	-2.0309141	3.0736666
H	1.7213367	-2.0320194	3.6400581
C	2.4416309	-1.9515147	-1.7177327
H	1.9264482	-1.6259954	-2.6244909
H	1.9270970	-2.8295516	-1.3216232
H	3.4745326	-2.2234226	-1.9792322
C	-1.1457405	-2.4609031	-0.8568915
C	-1.2611815	-3.7554943	-0.0820550
H	-0.5302213	-2.5170036	-1.7633009
H	-1.9942022	-4.4629197	-0.5080692
H	-0.2965947	-4.2755003	-0.0501575
H	-1.5642243	-3.5847801	0.9596550
C	-2.2322519	-1.5463474	-0.9466238
C	-2.1529430	-0.3857928	-1.7935271
C	-3.3947897	-1.5986586	-0.1050771
C	-3.1118997	0.6271938	-1.7655986
H	-1.3647243	-0.3498665	-2.5485031
C	-4.3342341	-0.5774497	-0.0853746
H	-3.5511340	-2.4750310	0.5181881
C	-4.2045222	0.5612176	-0.8975322
H	-3.0151307	1.4682785	-2.4482414
H	-5.1975530	-0.6713493	0.5701503
H	-4.9476313	1.3516814	-0.8740982
H	-1.9776294	-1.0846901	1.6302972
H	-1.3950489	-1.0390880	2.0990744

mB^{+}**.**THF : THF adduct

75

Energy = -1914.204830284

Ca	-0.5878900	2.4070327	1.5221463

N	0.5768068	3.7461625	3.4542189
N	-2.4623692	3.9012959	0.2991499
N	0.4933522	4.4554624	0.4954796
O	-1.6461509	0.8486745	0.0510668
C	-0.3813291	3.7980473	4.5882377
C	1.8566798	3.2105710	3.9721540
C	0.8189890	5.0979882	2.8892500
C	-1.7502255	4.8902986	-0.5475320
C	-3.4581815	3.2082855	-0.5471601
C	-3.1713337	4.5678079	1.4190480
C	-0.5193865	5.4795284	0.1250307
C	1.4358513	5.0271860	1.4962984
C	1.2463925	4.0361358	-0.7099157
C	-1.2155084	0.6048599	-1.3269476
C	-2.5348756	-0.2309138	0.4802370
C	-1.8136158	4.0719928	4.1553745
H	-0.0807116	4.5653187	5.3191168
H	-0.3202486	2.8324420	5.0987894
H	1.6960712	2.2079663	4.3694988
H	2.2522135	3.8582738	4.7695509
H	2.5943792	3.1519493	3.1703793
H	-0.1321682	5.6348489	2.8490907
H	1.4809830	5.6771828	3.5510599
H	-2.4206603	5.7147317	-0.8390933
H	-1.4648843	4.3749077	-1.4695343
H	-2.9521805	2.7214720	-1.3805130
H	-4.1962212	3.9221628	-0.9447451
H	-3.9832497	2.4474591	0.0313349
C	-3.5461821	3.5847345	2.5219551
H	-2.5253446	5.3509579	1.8233790
H	-4.0821189	5.0675830	1.0536806
H	-0.8091809	6.0126549	1.0331967
H	-0.0687479	6.2252032	-0.5472957
H	2.3248989	4.3906014	1.5106256
H	1.7629182	6.0305684	1.1857984
H	0.5621811	3.6134269	-1.4496231
H	1.9763924	3.2716734	-0.4346226
H	1.7736262	4.8853913	-1.1679729
C	-2.2928863	-0.2958123	-1.9096491
H	-0.2364198	0.1195078	-1.3068934
H	-1.1377040	1.5806741	-1.8155085
C	-2.6490210	-1.1862380	-0.7079252
H	-3.4972871	0.2238101	0.7410053
H	-2.0967614	-0.7007878	1.3646831
N	-2.3498304	3.0446604	3.2228483
H	-1.8751159	5.0431735	3.6594836
H	-2.4506875	4.1345530	5.0505484
H	-4.0907720	2.7354569	2.0999314
H	-4.2217100	4.0746319	3.2389879
H	-3.1596463	0.2923661	-2.2293830
H	-1.9250321	-0.8701867	-2.7629948
H	-3.6478115	-1.6219880	-0.7825167
H	-1.9176249	-1.9917704	-0.6108125
C	-2.7301678	1.8284094	3.9792765
H	-1.8593267	1.4235883	4.4983985
H	-3.1062689	1.0715672	3.2857631
H	-3.5127946	2.0475293	4.7199723
C	0.9788524	0.4878176	2.1764629
C	0.4188488	-0.2908470	3.3621827
H	1.8350507	1.1108303	2.4615785
C	1.2740274	-0.2512156	0.9697297
H	0.3574686	0.3423362	4.2570694
H	-0.5983018	-0.6696121	3.1819232
H	1.0227582	-1.1750038	3.6407044
C	0.7011790	-1.5179078	0.6617464
C	2.1505160	0.2780843	-0.0233943
C	0.9456943	-2.1634964	-0.5494411
H	0.0552971	-1.9989191	1.3921020
C	2.3913457	-0.3680939	-1.2288146
H	2.6608745	1.2185284	0.1862016
C	1.7801968	-1.5979798	-1.5193914
H	0.4810428	-3.1298200	-0.7374905
H	3.0681272	0.0843812	-1.9510691
H	1.9654534	-2.1043375	-2.4619243

mB^{+} : Markovnikov styrene adduct to **1m**$^{+}$

62

Energy = -1681.599680080

Ca	0.0084213	-0.1192872	-0.3668374
N	1.0292090	2.1355051	-0.0445554
N	-0.4414831	0.5556420	2.0274888
N	0.9530379	-1.9607911	1.0624204
N	2.4576380	-0.3465228	-0.9869101
C	1.1226858	2.4180462	1.4113343
H	1.3067243	3.4895545	1.5804641
H	1.9911941	1.8836122	1.8047747
C	-0.1342095	2.0044234	2.1654202
H	-0.0260861	2.2792058	3.2256103
H	-1.0001872	2.5508214	1.7809419
C	0.4868487	-0.2639431	2.8492139
H	0.2293397	-0.1760309	3.9156298
H	1.4959280	0.1410643	2.7345534
C	0.4666196	-1.7365406	2.4521659
H	1.0685771	-2.3142914	3.1689486
H	-0.5536473	-2.1257396	2.5052123
C	2.4368364	-1.8563436	1.0151069
H	2.8933029	-2.7736850	1.4154290
H	2.7404396	-1.0409712	1.6760437
C	2.9651552	-1.6127465	-0.3932005
H	4.0654493	-1.6208703	-0.3705478
H	2.6530140	-2.4245889	-1.0565686
C	3.1289792	0.8308840	-0.3743221
H	4.1615855	0.9136567	-0.7456347
H	3.1924175	0.6672645	0.7046505
C	2.3835630	2.1301570	-0.6587099
H	2.9792969	2.9807015	-0.2964236
H	2.2553364	2.2640704	-1.7358955
C	0.1759771	3.1447549	-0.7141914
H	-0.8259540	3.1327047	-0.2792679
H	0.0957762	2.8960447	-1.7751785

H	0.5951596	4.1558228	-0.6092677
C	-1.8368100	0.3313596	2.4709907
H	-2.0914828	-0.7260222	2.3899992
H	-2.5151164	0.8901772	1.8240468
H	-1.9726599	0.6526847	3.5145823
C	0.5242554	-3.3045389	0.6070711
H	0.8850825	-3.4876369	-0.4076276
H	-0.5674478	-3.3529605	0.6041065
H	0.9160712	-4.0922667	1.2670382
C	2.7203569	-0.3592969	-2.4454757
H	2.2643298	0.5129990	-2.9174549
H	2.2810436	-1.2576022	-2.8803821
H	3.8010906	-0.3535790	-2.6503106
C	-1.1052421	-0.1056147	-2.6204230
C	-0.5418859	-1.4517533	-3.0135700
H	-0.9600802	0.7197502	-3.3144179
H	-1.3124761	-2.1790963	-3.3183328
H	0.1714853	-1.3532548	-3.8351315
H	0.0108056	-1.9773408	-2.1974203
C	-2.2270125	-0.0501855	-1.7529296
C	-2.9591196	1.1585977	-1.4952067
C	-2.6103114	-1.1811694	-0.9493939
C	-4.0069370	1.2018504	-0.5926451
H	-2.7026570	2.0493349	-2.0656617
C	-3.6803461	-1.1165281	-0.0487047
H	-2.1434806	-2.1476905	-1.1349597
C	-4.3949945	0.0623321	0.1413644
H	-4.5478626	2.1361006	-0.4571510
H	-3.9573799	-2.0131649	0.5019594
H	-5.2232953	0.1082874	0.8411677

mC$^+$**.**THF : THF adduct of **mC**$^+$

65

Energy = -1682.990680386

Ca	0.1270306	0.2944884	-0.1146315
N	-1.6836805	0.2435658	-1.8660040
N	-0.4697189	-2.2050755	-0.6929428
N	-0.6166720	-0.8577826	2.0705677
N	-1.8777382	1.5672629	0.8736498
C	-2.3433640	-1.0819286	-1.9165113
H	-2.9703272	-1.1687224	-2.8179228
H	-3.0138705	-1.1630379	-1.0572644
C	-1.3354928	-2.2253195	-1.9008362
H	-1.8733954	-3.1827716	-1.9820965
H	-0.6772830	-2.1568147	-2.7713916
C	-1.2232784	-2.7085385	0.4844168
H	-1.3036188	-3.8058749	0.4374095
H	-2.2427185	-2.3196161	0.4290561
C	-0.5874260	-2.3217914	1.8152622
H	-1.0971200	-2.8663429	2.6255603
H	0.4618821	-2.6306680	1.8314324
C	-1.9953977	-0.4092078	2.3910563
H	-2.2685591	-0.7144953	3.4131562
H	-2.6866815	-0.9171971	1.7145669
C	-2.1569630	1.0987797	2.2539912
H	-3.1712288	1.3858270	2.5719362
H	-1.4587883	1.6143706	2.9196472
C	-3.0287868	1.2791279	-0.0199209
H	-3.8362513	2.0068810	0.1562530
H	-3.4295365	0.2968268	0.2440484
C	-2.6490203	1.3105776	-1.4971592
H	-3.5635545	1.2359186	-2.1061736
H	-2.1745275	2.2656132	-1.7388895
C	-1.0749454	0.5673701	-3.1760300
H	-0.3391924	-0.1945424	-3.4450971
H	-0.5649068	1.5291565	-3.1005468
H	-1.8367179	0.6111031	-3.9693156
C	0.7031855	-3.0749647	-0.9312236
H	1.3753120	-3.0384482	-0.0724456
H	1.2427929	-2.7183263	-1.8109196
H	0.3951266	-4.1176685	-1.1034412
C	0.2842335	-0.5457427	3.2012884
H	0.3347553	0.5346498	3.3571134
H	1.2860068	-0.9150881	2.9755866
H	-0.0633835	-1.0168363	4.1331841
C	-1.6161679	3.0236799	0.9002826
H	-1.3819664	3.3771262	-0.1039472
H	-0.7534371	3.2211578	1.5400628
H	-2.4867783	3.5737915	1.2898233
C	3.1282386	-0.6157959	-1.1092694
O	2.4031041	-0.5205288	0.1582071
C	3.3256289	-0.0481380	1.1974041
C	4.6826362	0.1363076	0.5151227
C	4.3067284	0.3311648	-0.9621199
H	3.4486265	-1.6546057	-1.2484064
H	2.4354080	-0.3318701	-1.9069167
H	2.9268217	0.8938177	1.5872955
H	3.3469896	-0.7958730	1.9941566
H	5.2310443	0.9845942	0.9307592
H	5.2942940	-0.7629690	0.6360603
H	3.9842920	1.3584937	-1.1514356
H	5.1237579	0.0819946	-1.6433563
C	1.1046912	2.3668230	-1.1110456
C	1.9268903	3.3248280	-0.2206284
H	0.2908086	2.9593062	-1.5788652
H	1.7406045	2.0725243	-1.9723603
H	1.3275786	3.7034712	0.6208180
H	2.7901753	2.8142025	0.2304448
H	2.3356280	4.2178722	-0.7290089

mC$^+$: ethylene adduct to terminal Ca-H bond

52

Energy = -1450.375961959

Ca	0.0341402	0.2293782	-0.1865753
N	-1.7739085	0.2693617	-1.9149582
N	-0.4735341	-2.1643659	-0.7062408
N	-0.5222968	-0.8452586	2.0075434
N	-1.8208603	1.5893823	0.7947871
C	-2.3785759	-1.0887421	-1.9331732
H	-3.0184331	-1.2097690	-2.8202272

H	-3.0295783	-1.1800419	-1.0596796
C	-1.3307838	-2.1989645	-1.9220694
H	-1.8362850	-3.1715017	-2.0161363
H	-0.6692112	-2.0998470	-2.7871526
C	-1.2029718	-2.7083354	0.4695204
H	-1.2595513	-3.8056554	0.4091220
H	-2.2302050	-2.3366880	0.4302097
C	-0.5571569	-2.3182359	1.7963544
H	-1.0934437	-2.8171960	2.6170452
H	0.4764170	-2.6739033	1.8300031
C	-1.8638113	-0.3367346	2.3992475
H	-2.0745259	-0.5872737	3.4497549
H	-2.6118512	-0.8566949	1.7949460
C	-1.9978656	1.1721752	2.2119299
H	-2.9761408	1.4970793	2.5960328
H	-1.2365627	1.6924892	2.7996348
C	-3.0386851	1.2825174	-0.0013608
H	-3.8355016	2.0064954	0.2263245
H	-3.4057635	0.3007048	0.3090207
C	-2.7764198	1.2895813	-1.5050760
H	-3.7275407	1.1386091	-2.0367906
H	-2.3908019	2.2648941	-1.8148234
C	-1.2277200	0.6137633	-3.2477704
H	-0.4776577	-0.1222124	-3.5461298
H	-0.7495600	1.5954624	-3.1984616
H	-2.0184222	0.6382182	-4.0115207
C	0.7689160	-2.9328004	-0.9493257
H	1.4115232	-2.8939982	-0.0667691
H	1.3081241	-2.4893768	-1.7904475
H	0.5513350	-3.9859360	-1.1788283
C	0.4791939	-0.5185892	3.0491294
H	0.5238170	0.5622083	3.2002874
H	1.4634428	-0.8644795	2.7232718
H	0.2312390	-0.9975894	4.0073490
C	-1.5121886	3.0366674	0.7347293
H	-1.3463441	3.3415969	-0.3011491
H	-0.6007540	3.2343529	1.3044504
H	-2.3319144	3.6396691	1.1513456
C	2.1985557	1.2931670	-0.5554176
C	2.6728497	2.0155540	0.7286881
H	2.1401462	2.0397286	-1.3717843
H	2.9960006	0.5963178	-0.8760988
H	1.9294737	2.7489693	1.0805793
H	2.8181795	1.3071794	1.5586696
H	3.6226084	2.5729747	0.6358138

mD$^+$**.**THF : THF adduct of **mD**$^+$
77
Energy = -1840.340597004

Ca	0.0425079	-0.3806853	0.4349919
N	2.0566771	0.4536086	1.9622082
N	0.0129201	-1.5996426	2.6303939
N	-0.1990899	2.1811223	0.7790838
H	-2.8079526	-1.8749718	-1.7011158
C	2.1743537	-0.4623755	3.1266289
C	3.3690825	0.5206391	1.2837532
C	1.6722871	1.8142563	2.4164324
C	0.8191122	-0.9078777	3.6620552
C	-1.4148851	-1.6867072	3.0303482
C	0.5362761	-2.9636332	2.3917075
C	-1.2777371	2.4199670	1.7726968
C	1.1009007	2.6808468	1.2989616
C	-0.5224952	2.8905097	-0.4782485
H	2.7473227	0.0095048	3.9401555
H	2.7509265	-1.3310220	2.7964365
H	3.6429651	-0.4730146	0.9232202
H	4.1516890	0.8778699	1.9707800
H	3.3154012	1.1973225	0.4302593
H	0.9414752	1.7117836	3.2219519
H	2.5445580	2.3283608	2.8496927
H	0.2535834	-0.0413834	4.0141631
H	0.9754249	-1.5609006	4.5351379
C	-2.1399777	-0.3486852	2.9229419
H	-1.8847293	-2.4175829	2.3667950
H	-1.5080370	-2.0669644	4.0597429
H	1.5794419	-2.9135351	2.0690982
H	-0.0499480	-3.4299656	1.5979765
H	0.4840843	-3.5761641	3.3047168
C	-2.5209443	1.5901366	1.4869150
H	-0.8908095	2.1728783	2.7638713
H	-1.5463510	3.4875478	1.7928206
H	1.7972191	2.7114668	0.4555116
H	0.9983125	3.7140811	1.6663176
H	-1.4492143	2.4978170	-0.9036609
H	0.2833650	2.7397136	-1.1982508
H	-0.6464314	3.9708021	-0.3092545
N	-2.2388952	0.1348241	1.5221717
H	-1.6189989	0.4102033	3.5125365
H	-3.1458387	-0.4517043	3.3594175
H	-3.3104991	1.8600839	2.2053187
H	-2.9080619	1.8217895	0.4904999
C	-3.3248996	-0.5863662	0.8227766
H	-3.3833691	-0.2312907	-0.2081927
H	-4.2937853	-0.4158386	1.3178057
H	-3.1048980	-1.6535374	0.8011651
C	-1.7638043	-2.0389817	-2.0067617
C	-0.9061454	-2.3718716	-0.7648493
H	-1.8081507	-2.8508045	-2.7573465
H	-1.4625839	-3.1015881	-0.1443494
H	-0.0123200	-2.9366062	-1.1030802
H	2.5404686	1.6510290	-1.7667380
C	2.0214769	0.8396791	-2.2893876
C	2.9039770	0.1854058	-3.3499734
H	1.0724407	1.2074961	-2.6875005
O	1.7285021	-0.2051890	-1.3081221
C	3.5927033	-0.9403022	-2.5639830
H	3.6060120	0.8967984	-3.7901229
H	2.2873638	-0.2356709	-4.1505318
C	2.4629374	-1.4202706	-1.6675495
H	4.4191945	-0.5446040	-1.9644456

H	3.9739738	-1.7373114	-3.2062224
H	1.7787041	-2.0961096	-2.1901270
H	2.7926096	-1.8910462	-0.7375833
C	-1.2877690	-0.7732831	-2.7343708
C	-2.1053936	-0.3763658	-3.9662502
H	-0.2363176	-0.9014197	-3.0257119
H	-1.3034690	0.0745198	-2.0239728
C	-1.5954854	0.9033694	-4.6393456
H	-2.0868980	-1.2008552	-4.6927755
H	-3.1580764	-0.2414968	-3.6779087
C	-2.4121248	1.3046759	-5.8717731
H	-1.6091423	1.7246124	-3.9079452
H	-0.5438077	0.7630191	-4.9265657
H	-2.0243070	2.2218812	-6.3286994
H	-2.3871071	0.5131951	-6.6300081
H	-3.4614264	1.4780568	-5.6049407

mTS10a$^+$: Markovnikov addition to **1m**$^+$
66
Energy = -1683.937714625

Ca	-0.2139013	0.3104605	-0.6298778
N	-1.2239879	0.3815709	1.7595004
N	1.7310528	0.1383758	1.0105056
N	1.4225440	2.2042826	-1.1377574
N	-1.5232453	2.4042975	-0.4138456
C	-0.0792909	0.6934165	2.6562626
H	-0.3687821	0.5361069	3.7063407
H	0.1565578	1.7552189	2.5530819
C	1.1551620	-0.1477547	2.3513426
H	1.9070093	0.0176172	3.1381800
H	0.9008396	-1.2121727	2.3738350
C	2.4330610	1.4491800	1.0201936
H	3.3927429	1.3607073	1.5518789
H	1.8238616	2.1584517	1.5851797
C	2.6827237	1.9842203	-0.3829487
H	3.2686713	2.9138769	-0.3159004
H	3.2832541	1.2722640	-0.9562533
C	0.7417558	3.4401987	-0.6702803
H	1.2477657	4.3291412	-1.0774487
H	0.8387455	3.4949827	0.4175243
C	-0.7306390	3.4792583	-1.0657778
H	-1.1464373	4.4695838	-0.8251635
H	-0.8289007	3.3352302	-2.1449629
C	-1.7840233	2.7132231	1.0117044
H	-2.5571352	3.4915480	1.1054698
H	-0.8682581	3.1254089	1.4433655
C	-2.2254906	1.4822506	1.7948945
H	-2.4434375	1.7741949	2.8336756
H	-3.1533435	1.0837038	1.3755164
C	-1.8819415	-0.8598818	2.2253302
H	-1.1635247	-1.6812368	2.2385464
H	-2.6964738	-1.1162683	1.5456413
H	-2.2874452	-0.7350798	3.2404467
C	2.6859665	-0.9364435	0.6548330
H	3.0962009	-0.7580183	-0.3419016
H	2.1687248	-1.8992464	0.6513462
H	3.5191474	-0.9816583	1.3718564
C	1.7440588	2.3242705	-2.5789534
H	0.8280817	2.4798325	-3.1483649
H	2.2002828	1.3931976	-2.9229881
H	2.4413239	3.1582527	-2.7567275
C	-2.7928454	2.2119879	-1.1508069
H	-3.3757992	1.4111186	-0.6897031
H	-2.5566726	1.9249727	-2.1782316
H	-3.3989306	3.1299961	-1.1513117
C	-1.6271390	-1.7174979	-1.3126839
C	-1.4917763	-1.1822320	-2.5789521
H	-2.5279100	-1.4899431	-0.7402076
H	-0.7575504	-1.5714017	-3.2745117
H	-2.3462818	-0.7118280	-3.0519580
H	-0.6115383	0.3904516	-2.6961192
C	-0.5959816	-2.5697439	-0.6163169
C	-1.2163097	-3.7942499	0.0829402
C	0.5491362	-3.0330747	-1.5313214
H	-0.1021743	-2.0044259	0.2220245
C	-0.1892775	-4.4872945	0.9852828
H	-1.5606614	-4.4868508	-0.6949067
H	-2.0978053	-3.4939404	0.6592380
C	1.4603166	-4.0120434	-0.8386710
H	0.1267578	-3.4859685	-2.4395775
H	1.1416317	-2.1762554	-1.8888066
C	1.1399125	-4.6449870	0.2956618
H	-0.5608601	-5.4708959	1.2985340
H	-0.0529043	-3.9158778	1.9179592
H	2.4288506	-4.1938991	-1.3016490
H	1.8604083	-5.3218977	0.7523127

mTS10$^+$: anti-Markovnikov addition to **1m**$^+$
66
Energy = -1683.937936004

Ca	0.3401240	0.2259172	0.4422248
N	0.5931326	1.3730300	-1.8166490
N	-1.9692737	0.3650282	-0.5988465
N	-1.0606862	1.4554508	2.0919088
N	1.4672416	2.4751047	0.8936507
C	-0.7599669	1.8392647	-2.2222257
H	-0.7537348	2.1491663	-3.2781683
H	-1.0032057	2.7281794	-1.6353957
C	-1.8319370	0.7740087	-2.0201325
H	-2.7883828	1.1467864	-2.4177150
H	-1.5803714	-0.1249222	-2.5905699
C	-2.6827512	1.4083042	0.1843252
H	-3.7609106	1.3753253	-0.0356213
H	-2.3249775	2.3882540	-0.1432076
C	-2.4762361	1.2541791	1.6882197
H	-3.1367056	1.9569020	2.2187125
H	-2.7565854	0.2451356	2.0012955
C	-0.6973419	2.8927106	2.0898250
H	-1.1199960	3.4016774	2.9698199
H	-1.1504484	3.3567516	1.2098881

C	0.8136958	3.1026150	2.0741401
H	1.0303297	4.1810236	2.1123686
H	1.2629563	2.6562787	2.9655618
C	1.2277763	3.2926640	-0.3243398
H	1.8558805	4.1965526	-0.3026700
H	0.1890951	3.6322576	-0.3062241
C	1.5058343	2.5314217	-1.6160544
H	1.4357573	3.2290016	-2.4645013
H	2.5271680	2.1401538	-1.6096099
C	1.1575484	0.4975460	-2.8689611
H	0.4932905	-0.3500770	-3.0456879
H	2.1294732	0.1194524	-2.5431451
H	1.2866034	1.0452597	-3.8141756
C	-2.7168118	-0.9123604	-0.5259190
H	-2.7622410	-1.2539298	0.5086163
H	-2.1911404	-1.6728197	-1.1071946
H	-3.7364359	-0.7947180	-0.9242366
C	-0.8365043	0.8575294	3.4277230
H	0.2018095	1.0075752	3.7339938
H	-1.0257416	-0.2164800	3.3621875
H	-1.4924848	1.3112465	4.1856116
C	2.9218207	2.3642046	1.1477264
H	3.4144469	1.8775412	0.3036018
H	3.0860172	1.7586644	2.0418971
H	3.3760152	3.3550024	1.2987455
C	2.4899230	-1.2627664	0.6478320
C	1.6131214	-2.2387301	1.0659107
H	3.0637222	-0.6840420	1.3673639
H	2.7660848	-1.1539144	-0.4001306
H	1.5632699	-2.4616135	2.1280909
H	-0.2043648	-1.5797370	1.3333205
C	1.1107845	-3.3400120	0.1523872
C	-0.0206510	-4.1827563	0.7531192
C	0.6913721	-2.8034252	-1.2233236
H	1.9698388	-4.0130480	-0.0075227
C	-0.3437986	-5.3870479	-0.1393718
H	-0.9017589	-3.5400287	0.8570123
H	0.2577862	-4.5178183	1.7594106
C	-0.0138907	-3.8292633	-2.0689075
H	0.0083306	-1.9469890	-1.0566642
H	1.5581746	-2.4136707	-1.7696096
C	-0.4609859	-4.9944107	-1.5877574
H	-1.2793360	-5.8594608	0.1873466
H	0.4313344	-6.1620888	-0.0354256
H	-0.1439138	-3.5940608	-3.1246449
H	-0.9361304	-5.7050671	-2.2629183

mTS1a$^+$: Markovnikov addition to **1m**$^+$**.**THF
79
Energy = -1916.544535978

Ca	0.0738902	0.4415908	-0.5347600
N	-1.6104065	-1.3732378	-1.6566607
N	1.2885450	-1.8281281	-0.8989970
N	-0.8171147	1.3053296	-2.8197821
H	0.2286172	2.5013022	-0.0640593
C	-0.9831915	-2.7083120	-1.4942719
C	-2.9818720	-1.4353426	-1.1079608
C	-1.6984669	-1.0090471	-3.0920139
C	0.5064113	-2.7080883	-1.8049694
C	2.6137777	-1.5301496	-1.5044929
C	1.4919253	-2.5217826	0.3913647
C	0.2789248	1.2429184	-3.8200954
C	-1.9572023	0.4776066	-3.2862778
C	-1.2699874	2.7081944	-2.6761530
H	-1.4777417	-3.4553977	-2.1362382
H	-1.1510671	-3.0200836	-0.4587754
H	-2.9458692	-1.7798040	-0.0754114
H	-3.6066104	-2.1317936	-1.6888700
H	-3.4454602	-0.4480891	-1.1309497
H	-0.7624451	-1.2876160	-3.5809341
H	-2.4963305	-1.5880502	-3.5837245
H	0.6737194	-2.3788517	-2.8322927
H	0.8833811	-3.7405923	-1.7420371
C	2.5162142	-0.5186547	-2.6407376
H	3.2530288	-1.1389345	-0.7082302
H	3.0859787	-2.4536347	-1.8741371
H	0.5294578	-2.7099747	0.8735476
H	2.0928243	-1.8901903	1.0458934
H	2.0051714	-3.4850353	0.2498533
C	1.6243700	1.6804239	-3.2642263
H	0.3425062	0.2192906	-4.1962115
H	0.0329497	1.8780253	-4.6857454
H	-2.8409767	0.7847107	-2.7196879
H	-2.1735169	0.6767953	-4.3476849
H	-0.4424592	3.3262153	-2.3279837
H	-2.0636809	2.7561205	-1.9269675
H	-1.6523373	3.0968786	-3.6337562
N	2.1214776	0.8262196	-2.1549519
H	1.7790970	-0.8551442	-3.3734507
H	3.4825755	-0.4684209	-3.1663960
H	2.3551175	1.7058822	-4.0893791
H	1.5502857	2.6959212	-2.8653678
C	3.3135277	1.4965106	-1.5900386
H	3.0346726	2.4915725	-1.2432714
H	4.1028453	1.5921798	-2.3522551
H	3.7094100	0.9270218	-0.7482014
C	1.7661101	0.9396192	1.5234540
C	1.4414380	2.2387980	1.1718113
H	2.6082750	0.4579869	1.0241167
C	1.1873558	0.1941779	2.7051341
H	0.8312568	2.8425114	1.8346953
H	2.1201633	2.8202496	0.5617152
C	2.2799505	-0.5539786	3.4935542
C	0.4241184	1.0978613	3.6851178
H	0.4703041	-0.5879568	2.3859733
C	1.6512668	-1.4957860	4.5284850
H	2.9179039	0.1868161	3.9935671
H	2.9283929	-1.1181746	2.8122697
C	0.0039332	0.3477706	4.9230121
H	1.0596017	1.9535614	3.9595420

H	-0.4576771	1.5309878	3.1974784
C	0.5376376	-0.8237284	5.2888445
H	2.4127105	-1.8546552	5.2332404
H	1.2609702	-2.3974013	4.0298429
H	-0.7783855	0.7949645	5.5357075
H	0.1706176	-1.3267325	6.1827845
H	-3.1974874	1.5730413	0.1590788
C	-2.5993266	1.6184609	1.0776601
C	-3.4776849	1.5180864	2.3176993
H	-1.9428282	2.4905025	1.0416895
O	-1.7359033	0.4311232	1.1129668
C	-3.6265010	-0.0018222	2.4836199
H	-4.4324820	2.0318831	2.1850159
H	-2.9675650	1.9491291	3.1852157
C	-2.2255938	-0.4891755	2.1414773
H	-4.3599834	-0.3948692	1.7717127
H	-3.9254391	-0.2974822	3.4918960
H	-1.5576960	-0.4325605	3.0061815
H	-2.1877395	-1.5014494	1.7322513

mTS1$^+$: anti-Markovnikov to **1m$^+$.**THF
79
Energy = -1916.550977256

Ca	-0.2581686	-0.1037536	0.5546809
N	1.3781451	0.7436178	2.5269007
N	-0.1798909	-1.8565203	2.4761661
N	-1.0590005	2.1796801	1.4602441
H	-1.3326764	0.4276883	-1.1876478
C	1.6251716	-0.4066166	3.4313237
C	2.6834013	1.2683291	2.0736493
C	0.6505747	1.8175142	3.2478678
C	0.3704689	-1.2245103	3.7011779
C	-1.5772606	-2.3035686	2.7185608
C	0.6492309	-3.0297355	2.1193433
C	-2.2740959	1.9187820	2.2730139
C	-0.0092709	2.8084475	2.2993781
C	-1.3935507	3.0952375	0.3453897
H	2.0434227	-0.0657710	4.3921784
H	2.3881753	-1.0327148	2.9594817
H	3.2240938	0.4863623	1.5390236
H	3.2925645	1.6029433	2.9280486
H	2.5359774	2.1128052	1.3990488
H	-0.1053834	1.3535951	3.8852089
H	1.3369435	2.3598534	3.9175322
H	-0.4028580	-0.5869049	4.1353548
H	0.6012954	-1.9960221	4.4527136
C	-2.5682907	-1.1441849	2.7617948
H	-1.8413970	-2.9884687	1.9080057
H	-1.6443075	-2.8740650	3.6585271
H	1.6658459	-2.7114075	1.8753711
H	0.2221662	-3.5241527	1.2464619
H	0.6997857	-3.7482981	2.9519192
C	-3.2245128	0.9264241	1.6178138
H	-1.9595278	1.5440924	3.2503625
H	-2.8144664	2.8611922	2.4565413
H	0.7372493	3.2332383	1.6216104
H	-0.4244494	3.6463022	2.8821119
H	-2.1327643	2.6267002	-0.3044561
H	-0.4948966	3.2799196	-0.2471314
H	-1.7758701	4.0560200	0.7261339
N	-2.6433594	-0.4307706	1.4656148
H	-2.2760239	-0.4288638	3.5345683
H	-3.5585642	-1.5310803	3.0499459
H	-4.1582267	0.8871138	2.2020880
H	-3.4817498	1.2684445	0.6118425
C	-3.4969279	-1.1878437	0.5253275
H	-3.4880014	-0.6742802	-0.4375704
H	-4.5302397	-1.2573043	0.9001426
H	-3.1068109	-2.1981037	0.3909144
C	-1.0716207	-1.3344790	-1.9438436
C	-0.4934238	-2.2169218	-1.0601571
H	-2.1539431	-1.3385559	-2.0371131
C	-0.3310908	-0.8036768	-3.1508892
H	-1.1012454	-2.8590935	-0.4290687
H	0.5749372	-2.4261191	-1.0896943
C	-0.1162806	-1.9213772	-4.1967409
C	-1.0230524	0.3814923	-3.8317186
H	0.6641525	-0.4800952	-2.8256332
C	0.8306316	-1.4418589	-5.3058939
H	-1.0875586	-2.1950942	-4.6308733
H	0.2856140	-2.8155532	-3.7093281
C	-0.3630337	0.7491340	-5.1334224
H	-2.0838831	0.1355496	-4.0009723
H	-1.0333266	1.2349220	-3.1438067
C	0.4869718	-0.0553986	-5.7825934
H	0.8056741	-2.1403009	-6.1529410
H	1.8705191	-1.4582253	-4.9415305
H	-0.5916712	1.7314198	-5.5462767
H	0.9585610	0.2869249	-6.7032694
H	2.0225805	2.4379617	-0.8300382
C	1.7605937	1.6285523	-1.5216596
C	2.8343332	1.4260338	-2.5835209
H	0.7552518	1.7896730	-1.9178464
O	1.7234777	0.3774590	-0.7575534
C	3.8115581	0.4731509	-1.8770209
H	3.2969796	2.3698857	-2.8805644
H	2.4056258	0.9531331	-3.4727430
C	2.8628298	-0.4563854	-1.1318669
H	4.4408314	1.0241826	-1.1703526
H	4.4583279	-0.0705641	-2.5694230
H	2.5006368	-1.2680507	-1.7733032
H	3.2804840	-0.8831453	-0.2161982

mTS20a$^+$: hydrogenoylsis with H_2
68
Energy = -1685.141465590

Ca	-0.3628934	-0.1541810	-0.4991923
N	-0.4544434	-1.2314507	1.8148817
N	-0.1525855	1.7284480	1.1849710
N	-2.2071282	1.5115414	-1.0065154

N	-2.4745688	-1.4456449	-0.3847941
C	-0.7508279	-0.1268010	2.7654362
H	-0.5934786	-0.4652319	3.8005997
H	-1.8102744	0.1254770	2.6761341
C	0.1054878	1.1093744	2.5127698
H	-0.0669088	1.8381684	3.3192699
H	1.1669330	0.8456003	2.5438809
C	-1.4562413	2.4447238	1.1925705
H	-1.3665823	3.3871403	1.7537526
H	-2.1817848	1.8273671	1.7275140
C	-1.9654469	2.7445663	-0.2111892
H	-2.8821017	3.3495765	-0.1406480
H	-1.2319307	3.3435841	-0.7583847
C	-3.4645572	0.8468682	-0.5702269
H	-4.3365589	1.3863560	-0.9699553
H	-3.5264558	0.9140070	0.5192945
C	-3.5367931	-0.6124685	-1.0076191
H	-4.5344058	-1.0129876	-0.7722488
H	-3.4044622	-0.6842627	-2.0905758
C	-2.7861735	-1.7418523	1.0353144
H	-3.5774542	-2.5037546	1.1063979
H	-3.1825808	-0.8317378	1.4930546
C	-1.5626397	-2.2257202	1.8061636
H	-1.8610352	-2.4823802	2.8338250
H	-1.1697166	-3.1392525	1.3516209
C	0.7916435	-1.9173625	2.2292978
H	1.6146825	-1.2020111	2.2758036
H	1.0415423	-2.6888430	1.4983962
H	0.6754318	-2.3816526	3.2195794
C	0.9390348	2.6813731	0.8765376
H	0.7748712	3.1333766	-0.1042234
H	1.8941191	2.1513929	0.8571012
H	0.9885042	3.4842645	1.6268714
C	-2.3046101	1.8795530	-2.4389581
H	-2.4953573	0.9883965	-3.0375264
H	-1.3553300	2.3120565	-2.7642144
H	-3.1100103	2.6118594	-2.6030407
C	-2.3115452	-2.6995413	-1.1562520
H	-1.5135770	-3.3054328	-0.7211980
H	-2.0361709	-2.4474921	-2.1830705
H	-3.2394723	-3.2899767	-1.1597938
C	1.5129248	-1.5087090	-1.4393553
C	2.1332028	-2.1882687	-2.6580985
H	1.2175699	-2.2994862	-0.7207131
H	3.0625378	-2.7367424	-2.4415187
H	1.4250307	-2.9085282	-3.0868003
H	2.3623839	-1.4640017	-3.4509907
C	2.4437913	-0.5284444	-0.7115286
C	3.7337603	-1.1404344	-0.1283946
C	2.8066715	0.6912673	-1.5810726
H	1.9213969	-0.1174539	0.1887895
C	4.4683305	-0.1388906	0.7711172
H	4.3871765	-1.4306179	-0.9603526
H	3.4929795	-2.0544087	0.4264388
C	3.8370966	1.5804399	-0.9374593
H	3.1673224	0.3498683	-2.5607000
H	1.9073082	1.2875278	-1.8100693
C	4.5632558	1.2190781	0.1267219
H	5.4754343	-0.5031296	1.0118026
H	3.9511388	-0.0494686	1.7409147
H	3.9758551	2.5696976	-1.3717461
H	5.2727584	1.9258445	0.5549492
H	0.2874706	-0.9089002	-2.3076786
H	-0.4711085	-0.5276238	-2.7090668

mTS20^{+} : hydrogenoylsis with H_2
68
Energy = -1685.140052006

C	-0.8845925	-5.8496572	0.3509744
C	0.0517306	-5.6734012	1.5167902
C	0.4946957	-4.2129926	1.6711554
C	0.9067107	-3.6026938	0.3239682
C	-0.2864152	-3.6284993	-0.6428438
C	-1.0511888	-4.9252293	-0.6012990
C	1.4692317	-2.1780698	0.4964839
C	1.9830362	-1.4557828	-0.7493213
Ca	0.7496326	0.6155369	-0.1476050
N	0.6608710	2.4088480	1.6336877
C	1.8299313	2.3883353	2.5451498
C	-0.5777765	2.2233280	2.4353133
C	-1.7709242	1.8237227	1.5771950
N	-1.5756810	0.5105242	0.9030139
C	-1.7858162	-0.5798630	1.8840739
C	-2.5558865	0.3498662	-0.2060823
C	-2.2351947	1.2185655	-1.4168253
N	-0.9312196	0.8772519	-2.0444208
C	-1.0755405	-0.3503004	-2.8615501
C	-0.4737409	1.9908769	-2.9210797
C	0.1103849	3.1545310	-2.1276352
N	1.2826195	2.7417974	-1.3173159
C	2.4693025	2.5501594	-2.1833220
C	0.6167497	3.7022225	0.8992321
C	1.5970193	3.7473471	-0.2679253
H	-3.0470765	1.1148782	-2.1522744
H	-2.2073947	2.2718412	-1.1278599
H	-3.5736863	0.5828564	0.1416316
H	-2.5517066	-0.7070331	-0.4889639
H	-2.6753189	1.7948831	2.2032650
H	-1.9445662	2.5798317	0.8079101
H	-0.8240820	3.1406666	2.9910304
H	-0.3705377	1.4482939	3.1787779
H	0.8360215	4.5341636	1.5853664
H	-0.4035167	3.8551930	0.5375092
H	1.6082106	4.7628403	-0.6920881
H	2.6083455	3.5301735	0.0863261
H	0.3857812	3.9617336	-2.8234364
H	-0.6446523	3.5643640	-1.4514628
H	-1.3005483	2.3558221	-3.5482301
H	0.2813665	1.5805578	-3.5966213
H	-1.4066008	-1.1809935	-2.2347225

H	-0.1095048	-0.6082257	-3.3009869
H	-1.8092962	-0.2059718	-3.6679487
H	-1.0489523	-0.5122064	2.6870627
H	-1.6678724	-1.5432987	1.3838020
H	-2.7927859	-0.5286925	2.3233035
H	2.7466548	2.5319315	1.9724543
H	1.8841639	1.4147628	3.0385543
H	1.7463073	3.1757548	3.3096600
H	2.2655659	1.7846876	-2.9356724
H	3.3044818	2.2153701	-1.5641328
H	2.7420475	3.4835023	-2.6972954
H	2.8998731	-1.9273890	-1.1177305
H	1.2517612	-1.5083129	-1.5773435
H	2.2734422	-2.2087955	1.2448292
H	0.6528782	-1.6045189	0.9967339
H	1.7042184	-4.2257148	-0.1065117
H	-0.3382552	-3.6264613	2.0861029
H	1.3233331	-4.1405016	2.3859082
H	-0.9697835	-2.7936707	-0.4005994
H	0.0548255	-3.4369967	-1.6677313
H	-0.4357825	-6.0233655	2.4361609
H	0.9313861	-6.3211436	1.3818127
H	-1.7615442	-5.1051070	-1.4076465
H	-1.4484443	-6.7803571	0.2996448
H	2.6870389	-0.1538925	-0.1917633
H	2.9426140	0.6411443	0.2767060

mTS2a$^+$: hydrogenoylsis with H_2
81
Energy = -1917.747324877

Ca	0.1603314	0.4190636	-0.2357393
N	1.1420205	0.3285453	2.1968227
N	-0.9980581	2.7368307	-0.6249512
N	1.9353089	2.2636928	-0.0064564
C	0.0432465	0.2008988	3.1865614
C	2.0720667	-0.8060121	2.3922261
C	1.8780980	1.6000682	2.4079092
C	0.0937332	3.7170971	-0.8707833
C	-1.9311728	2.7869423	-1.7728343
C	-1.7436725	3.0938823	0.6079503
C	1.2539685	3.5772203	0.1060667
C	2.7375114	1.9811956	1.2113319
C	2.8488291	2.2833264	-1.1702174
C	-1.1267742	1.1222682	2.8829735
H	0.4108546	0.4046869	4.2049763
H	-0.2871354	-0.8424796	3.1705600
H	1.5323711	-1.7392350	2.2299799
H	2.4876558	-0.7995674	3.4122204
H	2.8825700	-0.7532311	1.6650306
H	1.1523501	2.3907064	2.6129845
H	2.5194040	1.5226631	3.3001245
H	-0.2939247	4.7471112	-0.8230402
H	0.4455294	3.5519930	-1.8931701
H	-1.3743627	2.6263172	-2.6951514
H	-2.4322959	3.7653850	-1.8306415
H	-2.6930372	2.0098797	-1.6780261
C	-2.5972159	1.9472167	1.1278680
H	-1.0246178	3.3986655	1.3707100
H	-2.3895477	3.9652987	0.4176134
H	0.8988229	3.6956884	1.1322560
H	1.9698376	4.3944595	-0.0738621
H	3.4148220	1.1601947	0.9609399
H	3.3594600	2.8512217	1.4755259
H	2.2844649	2.4770523	-2.0833290
H	3.3314674	1.3077901	-1.2551900
H	3.6187699	3.0615929	-1.0531079
N	-1.7880530	0.7916486	1.5938248
H	-0.7835023	2.1578963	2.8419897
H	-1.8559462	1.0659422	3.7059309
H	-3.2601908	1.5812618	0.3384448
H	-3.2416110	2.3185558	1.9405772
C	-2.7085118	-0.3469450	1.8170929
H	-2.1520401	-1.2125176	2.1794945
H	-3.1967959	-0.6130674	0.8785862
H	-3.4779510	-0.0862525	2.5598729
H	-0.0835357	1.4911999	-4.4099677
C	-0.2996984	0.5539931	-3.8820046
C	-0.0138592	-0.6717004	-4.7709504
H	-1.3173356	0.5835314	-3.4940435
O	0.5996433	0.4531570	-2.7421349
C	1.3117434	-1.2467061	-4.2119956
H	0.0834844	-0.3688099	-5.8160989
H	-0.8202177	-1.4058661	-4.7072394
C	1.8130878	-0.1445471	-3.2865451
H	2.0341627	-1.4759049	-4.9989724
H	1.1244780	-2.1572099	-3.6376628
H	2.4010897	-0.4956706	-2.4389919
H	2.3642069	0.6288468	-3.8381311
H	0.0430330	-2.7428508	-1.5708264
C	-0.3454428	-2.0849086	-0.7817173
C	-1.6354406	-1.4535207	-1.3180993
H	-1.9024333	-0.4551330	-0.8941704
C	-0.4850464	-2.9220802	0.4979836
H	-1.5709589	-1.3044288	-2.3980827
H	-2.5409865	-2.0485583	-1.1326422
C	-1.7526969	-3.7971927	0.5840670
C	0.7430991	-3.8336293	0.6633399
H	-0.5097684	-2.2593710	1.3837975
C	-1.7098907	-4.7706430	1.7337367
H	-1.8800229	-4.3413717	-0.3658528
H	-2.6487439	-3.1715771	0.6851003
C	0.7631825	-4.5371236	2.0263375
H	0.7108347	-4.5884462	-0.1348298
H	1.6640967	-3.2587777	0.5150274
C	-0.5897753	-5.0844082	2.3945842
H	-2.6537186	-5.2251524	2.0355361
H	1.5006667	-5.3510608	2.0227316
H	1.1003129	-3.8435442	2.8132743
H	-0.6361653	-5.7753225	3.2359259
H	1.1066820	-1.3938407	-0.6162230

H	1.9042862	-0.9086182	-0.4619669

mTS2$^+$: hydrogenoylsis with H_2

81

Energy = -1917.750495878

Ca	0.4038095	0.7423361	0.1828840
N	1.4486389	0.9058787	2.5197494
N	-1.7104242	2.3398144	0.0402038
N	1.1322455	3.1837170	0.5214517
C	0.5688378	0.2517894	3.5214144
C	2.7697113	0.2351851	2.5450704
C	1.6161666	2.3470626	2.8401359
C	-1.2113557	3.7325277	-0.0999986
C	-2.5664264	2.0272114	-1.1251389
C	-2.5133692	2.2208641	1.2810828
C	-0.0447227	4.0284021	0.8333212
C	2.1002141	3.1627795	1.6486135
C	1.8253068	3.7253929	-0.6687902
C	-0.8976868	0.6113455	3.3340881
H	0.8803630	0.5175189	4.5438013
H	0.7076233	-0.8283163	3.4167668
H	2.6458042	-0.8101122	2.2551789
H	3.2147710	0.2852341	3.5505065
H	3.4363569	0.7138301	1.8269434
H	0.6580102	2.7342630	3.1953890
H	2.3302586	2.4694120	3.6694037
H	-2.0215774	4.4552187	0.0852484
H	-0.9005679	3.8620552	-1.1402604
H	-1.9793881	2.1409373	-2.0368132
H	-3.4392447	2.6968643	-1.1677737
H	-2.9238100	0.9966079	-1.0633494
C	-2.7166343	0.7794261	1.7170644
H	-2.0114665	2.7847385	2.0702247
H	-3.4997924	2.6903675	1.1397893
H	-0.3380825	3.8450910	1.8698694
H	0.2144770	5.0963613	0.7623933
H	3.0245684	2.7312032	1.2539338
H	2.3258915	4.1882207	1.9828190
H	1.1349264	3.7780879	-1.5129465
H	2.6487687	3.0556469	-0.9262986
H	2.2124391	4.7372059	-0.4722564
N	-1.4517626	0.0793807	2.0615414
H	-1.0146607	1.6969934	3.3360396
H	-1.4787610	0.2305610	4.1884591
H	-3.1917381	0.2061508	0.9151988
H	-3.4091011	0.7619257	2.5741066
C	-1.7877372	-1.3495810	2.2573216
H	-0.9028675	-1.9075329	2.5646976
H	-2.1579958	-1.7703520	1.3209516
H	-2.5614266	-1.4626542	3.0323270
H	-1.6407775	0.2792157	-3.3823814
C	-0.7901583	-0.2827788	-2.9793475
C	0.0782296	-0.8505422	-4.0897241
H	-1.1538016	-1.0260793	-2.2667329
O	0.0781555	0.6408755	-2.2506481
C	0.9187984	0.3740981	-4.4788629
H	-0.5163453	-1.2422985	-4.9184240
H	0.7170744	-1.6504705	-3.7031191
C	1.1882694	1.0405545	-3.1309476
H	0.3407881	1.0386845	-5.1286912
H	1.8469933	0.1104371	-4.9907432
H	2.1120695	0.6895890	-2.6641936
H	1.1951024	2.1313029	-3.1836198
H	0.3999439	-2.4089501	0.9260028
C	0.4246963	-2.1512436	-0.1412774
C	1.7404410	-1.4473592	-0.4724769
H	-0.4633606	-1.4911709	-0.2933638
C	0.1082476	-3.4259663	-0.9485604
H	2.5689246	-1.9455331	0.0473250
H	1.9418057	-1.5330032	-1.5515202
C	1.1313950	-4.5348731	-0.6686256
C	-1.3099723	-3.9508965	-0.6907019
H	0.1827371	-3.1681799	-2.0161735
C	0.7161604	-5.8579782	-1.2572460
H	1.2696181	-4.6393152	0.4204392
H	2.1126248	-4.2434175	-1.0630209
C	-1.6480818	-5.1185646	-1.6272989
H	-1.3820906	-4.2876646	0.3529930
H	-2.0430877	-3.1425488	-0.8185712
C	-0.5188883	-6.1134509	-1.7039744
H	1.4800386	-6.6324667	-1.3210080
H	-2.5633178	-5.6244969	-1.2927575
H	-1.8713245	-4.7380718	-2.6363037
H	-0.7416852	-7.0848754	-2.1440256
H	2.3003162	0.0064938	-0.3406450
H	2.5893102	0.9119551	-0.3094746

mTS30$^+$: H_2 isotope exchange via **1m**$^+$

48

Energy = -1372.873147233

Ca	-1.2354968	0.0015701	0.0002796
H	-3.1693212	1.0399799	-0.0278310
N	0.0774705	1.6593122	1.3248891
N	0.0767366	-1.3148603	1.6707094
N	0.0738297	-1.6588191	-1.3245230
N	0.0791405	1.3153037	-1.6703210
C	1.1663190	0.9129693	2.0091634
H	1.6398978	1.5451922	2.7749987
H	1.9365941	0.6828757	1.2683792
C	0.6675700	-0.3731393	2.6603877
H	1.4958216	-0.8487702	3.2057053
H	-0.1090773	-0.1462336	3.3957617
C	1.1476265	-2.0153448	0.9119668
H	1.6116679	-2.7909976	1.5392366
H	1.9294480	-1.2870462	0.6806434
C	0.6330897	-2.6571054	-0.3728772
H	1.4479669	-3.2281526	-0.8418904
H	-0.1655356	-3.3680701	-0.1447006
C	1.1640811	-0.9147424	-2.0089946
H	1.6361811	-1.5478844	-2.7750018

H	1.9350000	-0.6862618	-1.2683802
C	0.6678584	0.3723938	-2.6601297
H	1.4969822	0.8463181	-3.2056087
H	-0.1093959	0.1470762	-3.3953479
C	1.1516155	2.0135598	-0.9117803
H	1.6171468	2.7882490	-1.5391349
H	1.9319697	1.2836554	-0.6805644
C	0.6385913	2.6564039	0.3731263
H	1.4547147	3.2258630	0.8419339
H	-0.1586075	3.3689925	0.1450544
C	-0.7778919	2.3505285	2.3186142
H	-1.2110699	1.6227618	3.0083725
H	-1.5910704	2.8611006	1.7976966
H	-0.2005286	3.0842938	2.8997197
C	-0.7835639	-2.2973523	2.3722834
H	-1.2407427	-2.9763335	1.6498579
H	-1.5801198	-1.7660537	2.8988394
H	-0.2045704	-2.8867497	3.0980736
C	-0.7831435	-2.3482840	-2.3180873
H	-1.2148630	-1.6196557	-3.0078458
H	-1.5973562	-2.8570530	-1.7970294
H	-0.2074055	-3.0833271	-2.8991905
C	-0.7792500	2.2996057	-2.3717099
H	-1.2353868	2.9790941	-1.6491185
H	-1.5766669	1.7699287	-2.8985775
H	-0.1990142	2.8882003	-3.0971633
H	-3.3340958	0.0048023	-0.0035780
H	-3.1728051	-1.0306594	0.0213304

mTS3$^+$: H_2 isotope exchange via **1m$^+$.**THF
61
Energy = -1605.491394244

Ca	0.2150446	-0.2583944	-0.3177775
N	-0.1435846	1.5481475	1.4993699
C	-0.0141177	0.8857268	2.8236358
C	-1.5196622	2.0736491	1.3106125
C	0.8301617	2.6604530	1.4246103
C	-0.6801397	-0.4851058	2.8667561
H	-0.4382840	1.5172621	3.6196044
H	1.0552722	0.7893322	3.0316065
H	-1.6663473	2.9862145	1.9088895
H	-2.2242881	1.3305119	1.6924150
C	-1.8268879	2.3786290	-0.1508132
H	0.7592555	3.1563848	0.4543138
H	1.8399706	2.2622193	1.5338575
H	0.6422081	3.4046071	2.2133466
N	-0.0945516	-1.4315756	1.8849753
H	-0.5973630	-0.8908879	3.8868841
H	-1.7476203	-0.3901092	2.6521038
N	-1.7721821	1.1641831	-1.0058799
H	-2.8135581	2.8613374	-0.2216048
H	-1.0965748	3.0894531	-0.5475191
C	-1.0050401	-2.5849172	1.6559182
C	1.2133640	-1.9292972	2.3682057
C	-2.9941251	0.3415173	-0.8033324
C	-1.6666938	1.5696830	-2.4267124
C	-2.1922542	-2.2244220	0.7683325
H	-1.3723506	-2.9857659	2.6130877
H	-0.4109149	-3.3699233	1.1805202
H	1.8940811	-1.0906841	2.5349078
H	1.6432586	-2.5838952	1.6063102
H	1.1070481	-2.4831522	3.3131708
H	-3.8515021	0.8083698	-1.3124006
H	-3.2268274	0.3383829	0.2646087
C	-2.8364114	-1.0879914	-1.3056433
H	-1.5881653	0.6845334	-3.0593631
H	-0.7632438	2.1697603	-2.5632761
H	-2.5386548	2.1659657	-2.7363875
N	-1.7661944	-1.8345235	-0.5979720
H	-2.8835100	-3.0806448	0.7272768
H	-2.7493440	-1.3919332	1.2066145
H	-3.8037315	-1.6070069	-1.2144271
H	-2.5715034	-1.0847881	-2.3666136
C	-1.4315128	-3.0423214	-1.3864077
H	-0.6454078	-3.6068969	-0.8842957
H	-1.0606112	-2.7306686	-2.3650409
H	-2.3160749	-3.6849856	-1.5147682
H	3.1624002	2.5147746	-1.2871979
C	2.6766714	1.6230477	-1.7012276
C	3.6068349	0.8396432	-2.6365044
O	2.3801763	0.7149793	-0.6031899
H	1.7254153	1.9056853	-2.1574102
C	4.2093067	-0.2745147	-1.7347004
H	4.3754502	1.4927210	-3.0559656
H	3.0402500	0.3996099	-3.4592103
C	3.6279254	0.0083485	-0.3476385
H	3.8879154	-1.2589647	-2.0812295
H	5.3012761	-0.2514429	-1.7182431
H	3.3784020	-0.8886549	0.2207866
H	4.2759771	0.6636179	0.2475562
H	1.3665748	-2.0536844	-0.9641267
H	1.0242974	-1.5362245	-1.8069245
H	0.5595979	-0.8528827	-2.4380872

mTS4a$^+$: anti-Markovnikov styrene addition
62
Energy = -1681.544456877

Ca	0.0561901	0.2031245	0.6926005
N	1.8168622	1.1669620	-0.8801732
N	-0.9783190	0.3010083	-1.5891738
N	-2.0090558	1.5781195	0.9639369
N	0.7550589	2.4281026	1.6723308
C	1.0739001	1.6415127	-2.0787244
H	1.7777148	1.8983515	-2.8847545
H	0.5500843	2.5628323	-1.8120726
C	0.0761068	0.6076998	-2.5894886
H	-0.3662243	0.9659537	-3.5313738
H	0.5888167	-0.3317145	-2.8151897
C	-1.9879693	1.3904626	-1.5388119
H	-2.6620837	1.3224710	-2.4064000

H	-1.4634921	2.3462892	-1.6227107
C	-2.8218390	1.3612764	-0.2614171
H	-3.6221336	2.1134049	-0.3367711
H	-3.3013301	0.3848635	-0.1533143
C	-1.6148389	3.0004411	1.1113565
H	-2.4694018	3.6081546	1.4465939
H	-1.3250651	3.3785339	0.1271273
C	-0.4618889	3.1720372	2.0965366
H	-0.2417133	4.2429472	2.2189855
H	-0.7490433	2.7914635	3.0802931
C	1.4622629	3.1717137	0.5973126
H	1.9977759	4.0342867	1.0225057
H	0.7094192	3.5748662	-0.0847771
C	2.4563715	2.3084566	-0.1710504
H	3.0058256	2.9449070	-0.8814400
H	3.1966526	1.8860518	0.5146257
C	2.8764850	0.2196051	-1.2956773
H	2.4367223	-0.6148110	-1.8445976
H	3.3840891	-0.1683212	-0.4092536
H	3.6176299	0.7130518	-1.9418085
C	-1.6281470	-0.9861235	-1.9326049
H	-2.3606708	-1.2432787	-1.1665048
H	-0.8724491	-1.7743456	-1.9534825
H	-2.1224115	-0.9289425	-2.9146242
C	-2.7790205	1.1276661	2.1458182
H	-2.1856893	1.2638487	3.0531629
H	-2.9962352	0.0635640	2.0324494
H	-3.7154513	1.6959216	2.2519020
C	1.6508904	2.2321071	2.8352372
H	2.5355455	1.6662037	2.5345973
H	1.1222971	1.6652479	3.6057219
H	1.9752193	3.1942560	3.2584709
C	1.3832444	-1.5884164	2.0472855
C	0.3006101	-2.3652063	1.6576507
H	1.4808233	-1.2530329	3.0755754
H	2.2491916	-1.4473391	1.4044803
H	-0.3610161	-2.7554742	2.4230261
H	-1.1550596	-1.4313760	1.1818594
C	0.2765584	-3.1265131	0.3768889
C	-0.8342205	-3.9281838	0.0654098
C	1.3294385	-3.0674296	-0.5466439
C	-0.8971905	-4.6353586	-1.1299781
H	-1.6628311	-3.9660519	0.7679991
C	1.2597848	-3.7577506	-1.7612867
H	2.2252215	-2.5012078	-0.3131131
C	0.1468415	-4.5420157	-2.0591458
H	-1.7647431	-5.2519039	-1.3481535
H	2.0854657	-3.6928496	-2.4647931
H	0.0925876	-5.0823255	-2.9997489

mTS4$^+$: Markovnikov styrene addition

62

Energy = -1681.556649605

Ca	0.2980770	-0.6150616	-0.0789471
N	0.7786790	1.3312788	-1.7358115
N	-0.2195704	1.5567817	1.1368842
N	1.6969409	-0.5392256	2.0399727
N	2.6638088	-0.7869150	-0.8278728
C	0.8578581	2.5528011	-0.8892806
H	0.8466789	3.4508702	-1.5250218
H	1.8199010	2.5474525	-0.3714533
C	-0.2778543	2.6409907	0.1217877
H	-0.2550454	3.6283864	0.6074467
H	-1.2435149	2.5566443	-0.3859851
C	0.8708297	1.8159991	2.1125802
H	0.5780955	2.6207278	2.8040298
H	1.7447649	2.1737994	1.5628485
C	1.2345180	0.5719576	2.9110512
H	1.9995968	0.8302421	3.6588587
H	0.3617447	0.2103341	3.4615974
C	3.0948391	-0.3040282	1.5918290
H	3.7970697	-0.5282014	2.4093613
H	3.2071337	0.7596191	1.3643025
C	3.4638238	-1.1430948	0.3735035
H	4.5399591	-1.0353620	0.1692426
H	3.2727276	-2.1997539	0.5782751
C	3.1215065	0.4920934	-1.4204079
H	4.0745638	0.3535274	-1.9536737
H	3.3153102	1.1956057	-0.6065632
C	2.0945500	1.0743858	-2.3842259
H	2.4951772	1.9993868	-2.8257939
H	1.9167450	0.3790677	-3.2090565
C	-0.2330279	1.5439699	-2.7959437
H	-1.2134077	1.7162233	-2.3479304
H	-0.2850277	0.6579006	-3.4316664
H	0.0253952	2.4123144	-3.4199397
C	-1.5202406	1.4852273	1.8404962
H	-1.5100537	0.6711553	2.5682494
H	-2.3113053	1.2903629	1.1142563
H	-1.7380673	2.4256129	2.3685330
C	1.6207477	-1.8095937	2.7993640
H	1.9421012	-2.6367981	2.1668569
H	0.5832882	-1.9918709	3.0894809
H	2.2479580	-1.7631738	3.7031964
C	2.7608174	-1.8832095	-1.8194210
H	2.1671904	-1.6412499	-2.7044379
H	2.3585512	-2.7930322	-1.3688402
H	3.8029913	-2.0457177	-2.1318671
C	-1.7100285	-1.6030240	-1.6976132
C	-1.1030247	-2.8052878	-1.4785770
H	-1.4385573	-1.0262631	-2.5803841
H	-1.4666313	-3.5069058	-0.7411845
H	-0.3678830	-3.1920386	-2.1759169
H	0.3606728	-2.7190656	0.1322841
C	-2.7211887	-1.0192386	-0.8173873
C	-3.4618170	0.1123056	-1.2248051
C	-2.9823768	-1.5506950	0.4674837
C	-4.4474306	0.6586403	-0.4075834
H	-3.2803853	0.5366165	-2.2091453
C	-3.9697407	-0.9983895	1.2816640

H -2.4181220 -2.4113545 0.8182702
C -4.7128342 0.1021721 0.8485266
H -5.0152532 1.5184421 -0.7518481
H -4.1592079 -1.4297410 2.2606042
H -5.4841356 0.5271297 1.4835131

mTS5a$^+$: hydrogenolysis with H_2
64
Energy = -1682.748687727

Ca	-0.3356173	-0.1669739	-0.5885175
N	-2.7390482	0.1545963	-1.1716525
N	-1.6609905	-2.0098982	0.5797831
N	0.1922328	-0.0092450	1.9021343
N	-0.8898584	2.1861500	0.1286332
C	-3.5705761	-0.5852543	-0.1914991
H	-4.6162153	-0.6377314	-0.5313151
H	-3.5714650	-0.0273882	0.7486547
C	-3.0486619	-1.9987463	0.0391558
H	-3.7304645	-2.5354165	0.7154993
H	-3.0339150	-2.5524595	-0.9033873
C	-1.6777798	-1.6802348	2.0301476
H	-2.0237345	-2.5483901	2.6111528
H	-2.4107590	-0.8855696	2.1884847
C	-0.3139690	-1.2476413	2.5530861
H	-0.3742686	-1.1154470	3.6441710
H	0.4254927	-2.0321617	2.3680505
C	-0.5552914	1.1787534	2.3943625
H	-0.2384222	1.4255792	3.4189639
H	-1.6156133	0.9197358	2.4460867
C	-0.3626035	2.3985895	1.5027250
H	-0.8410080	3.2706380	1.9734725
H	0.7018889	2.6306616	1.4082860
C	-2.3756139	2.2718112	0.1210690
H	-2.6927086	3.3246265	0.1656769
H	-2.7501363	1.7932805	1.0295698
C	-2.9951121	1.6176258	-1.1090643
H	-4.0761199	1.8249659	-1.1223085
H	-2.5660814	2.0508339	-2.0165125
C	-3.0163294	-0.3289209	-2.5443716
H	-2.7772449	-1.3917621	-2.6243106
H	-2.3833788	0.2218121	-3.2426028
H	-4.0746924	-0.1857168	-2.8079836
C	-1.0826039	-3.3595779	0.3792624
H	-0.0817398	-3.4064894	0.8128068
H	-1.0110684	-3.5684290	-0.6907247
H	-1.7049488	-4.1316042	0.8548438
C	1.6301032	0.1425415	2.2274089
H	2.0515312	0.9991283	1.6981003
H	2.1666376	-0.7513880	1.9080063
H	1.7757001	0.2822484	3.3087784
C	-0.3245045	3.2215277	-0.7684872
H	-0.7006738	3.0761705	-1.7817886
H	0.7630844	3.1226464	-0.7875924
H	-0.5870487	4.2315176	-0.4187320
C	0.9820881	-1.4770765	-2.3386874
C	1.9627738	-1.7959149	-1.1995399
H	1.5432597	-1.3538683	-3.2733869
H	0.2965692	-2.3264925	-2.4889438
H	1.4063072	-2.0309847	-0.2646043
H	2.5401926	-2.7181537	-1.3671731
C	2.9315240	-0.6753181	-0.8601834
C	4.0572877	-0.9373818	-0.0637820
C	2.7020370	0.6475084	-1.2663326
C	4.9081867	0.0894371	0.3386333
H	4.2609251	-1.9594240	0.2483079
C	3.5512015	1.6826729	-0.8576494
H	1.8754505	0.8774358	-1.9380923
C	4.6520087	1.4090723	-0.0484018
H	5.7729935	-0.1371443	0.9560432
H	3.3578774	2.6997045	-1.1880267
H	5.3136295	2.2104212	0.2669546
H	0.2596115	-0.1154421	-2.5832991
H	-0.1972459	0.7405488	-2.6183645

mTS5$^+$**.**THF : hydrogenolysis with H_2
77
Energy = -1915.365223826

Ca	0.2006407	0.5922102	0.0122886
N	0.9977810	2.1661836	1.8525578
N	-0.5815107	1.8783035	-2.1418916
N	2.1441213	1.9028887	-0.9615556
O	-0.8036175	-1.2856474	-1.0087814
C	-0.1853683	2.8333739	2.4556261
C	1.7608330	1.4815229	2.9229354
C	1.8810582	3.1603449	1.1868679
C	0.6114480	2.4121531	-2.8513270
C	-1.3553358	1.0578974	-3.1007580
C	-1.4285942	3.0002197	-1.6610066
C	1.6623776	2.9510464	-1.8930166
C	2.8440585	2.5112119	0.1996896
C	3.0815452	0.9976201	-1.6670337
C	0.0195327	-2.2208061	-1.7851390
C	-2.1829833	-1.7765286	-0.9705790
C	-1.2146534	3.2543240	1.4145671
H	0.1170681	3.7167085	3.0388654
H	-0.6301699	2.1244164	3.1598478
H	1.1148690	0.7570105	3.4188361
H	2.1296142	2.2065999	3.6638489
H	2.6064878	0.9467733	2.4880894
H	1.2523138	3.8923215	0.6735243
H	2.4582854	3.7153128	1.9419859
H	0.3196727	3.2030821	-3.5590996
H	1.0280185	1.5931910	-3.4438973
H	-0.7448542	0.2159967	-3.4316122
H	-1.6515384	1.6531379	-3.9775380
H	-2.2564999	0.6705365	-2.6241207
C	-2.4245073	2.5694131	-0.5934942
H	-0.7721579	3.7804370	-1.2683978
H	-1.9794484	3.4472037	-2.5028137
H	1.2471338	3.7723776	-1.3037681

H 2.5006914 3.3686329 -2.4715185
H 3.3984553 1.7074487 0.6915645
H 3.5769291 3.2597190 -0.1389260
H 2.5800740 0.5258684 -2.5155595
H 3.3979074 0.2157448 -0.9733674
H 3.9568019 1.5496394 -2.0415987
C -0.9651625 -2.8956687 -2.7255717
H 0.4900118 -2.9246563 -1.0937927
H 0.7879117 -1.6269116 -2.2888663
C -2.2154150 -3.0350722 -1.8410461
H -2.8221861 -0.9796250 -1.3637803
H -2.4454446 -1.9755145 0.0709589
N -1.7726832 2.0988036 0.6593709
H -0.7587949 3.9413175 0.6981102
H -2.0259437 3.8076223 1.9109413
H -3.0415217 1.7455924 -0.9646752
H -3.1066104 3.4059432 -0.3778453
H -1.1692510 -2.2608499 -3.5938169
H -0.5937585 -3.8605579 -3.0781292
H -3.1387260 -3.0997904 -2.4208598
H -2.1306799 -3.9246551 -1.2137042
C -2.8001864 1.4217723 1.4854838
H -2.3691283 1.1045996 2.4352159
H -3.1706828 0.5401071 0.9568844
H -3.6464610 2.0943505 1.6877116
H 1.9561002 -0.5620879 0.5634361
H 1.3404331 -0.9042131 1.2083212
C 0.5140630 -1.6912295 2.2766301
C -0.8302582 -1.3000948 2.8696433
H 1.3308609 -1.5331679 2.9909103
C 0.6053474 -3.0022380 1.6405531
H -0.7848644 -0.3106711 3.3415588
H -1.6253322 -1.2630809 2.1125844
H -1.1870860 -1.9990579 3.6462877
C -0.5203073 -3.8083715 1.3444418
C 1.8635943 -3.4895248 1.1981190
C -0.3984398 -5.0060625 0.6404462
H -1.5046112 -3.4973212 1.6842704
C 1.9803920 -4.6796491 0.4869474
H 2.7552557 -2.9072127 1.4185442
C 0.8488118 -5.4510639 0.1927202
H -1.2864825 -5.6042866 0.4469128
H 2.9625798 -5.0134412 0.1591007
H 0.9406329 -6.3828592 -0.3578852

mTS5$^+$: hydrogenolysis with H_2
64
Energy = -1682.761724532
Ca 0.0096897 0.2297028 0.2577910
N 0.2239430 -0.5236821 -2.1622021
N 1.5132543 1.8807988 -0.9510209
N 2.1790645 0.2194169 1.5072055
N 0.9004630 -2.1867040 0.2880118
C 1.4342317 0.1300457 -2.7257284
H 1.4722439 -0.0176311 -3.8157403
H 2.3165262 -0.3611688 -2.3082824
C 1.4758028 1.6189694 -2.4135888
H 2.3434420 2.0737229 -2.9147061
H 0.5852178 2.1130194 -2.8108539
C 2.8867161 1.6554561 -0.4250683
H 3.5308292 2.5120626 -0.6751875
H 3.3087449 0.7871978 -0.9376957
C 2.9135075 1.4408142 1.0832907
H 3.9601266 1.4015136 1.4226644
H 2.4334837 2.2840866 1.5868048
C 2.9306986 -1.0110173 1.1609067
H 3.7927431 -1.1407948 1.8330632
H 3.3321488 -0.8962730 0.1505312
C 2.0485089 -2.2505444 1.2341751
H 2.6574529 -3.1463000 1.0411890
H 1.6333694 -2.3575307 2.2397542
C 1.3609938 -2.4868973 -1.0950059
H 1.5125809 -3.5701121 -1.2144545
H 2.3368858 -2.0172585 -1.2386075
C 0.3832336 -2.0023783 -2.1582289
H 0.7180496 -2.3585881 -3.1444298
H -0.6076474 -2.4313707 -1.9818875
C -0.9574714 -0.1717054 -2.9835380
H -1.1266114 0.9069541 -2.9589667
H -1.8390069 -0.6646041 -2.5725022
H -0.8191663 -0.4834269 -4.0294173
C 1.1040776 3.2841483 -0.7052023
H 1.1656192 3.5008689 0.3612319
H 0.0651799 3.4161069 -1.0188412
H 1.7431757 3.9832331 -1.2659023
C 1.9533970 0.2812137 2.9704606
H 1.3528935 -0.5706848 3.2957928
H 1.4081890 1.1983009 3.1999848
H 2.9079455 0.2714086 3.5174061
C -0.1024618 -3.1969460 0.6983140
H -0.9298287 -3.2110138 -0.0132542
H -0.4910361 -2.9430491 1.6873491
H 0.3439327 -4.2014169 0.7372718
C -1.7194929 0.2209712 2.3794041
C -2.1649540 1.0761374 3.5654721
H -1.4521297 -0.7946022 2.6975419
H -3.1152881 0.7319514 3.9940754
H -1.4063753 1.0526911 4.3562678
H -2.2903341 2.1249682 3.2703525
C -2.5699278 0.2138114 1.1855889
C -2.9108909 -0.9850079 0.5052545
C -2.9652960 1.4336690 0.5682862
C -3.6426695 -0.9692155 -0.6806450
H -2.6514149 -1.9359024 0.9632706
C -3.6871986 1.4384439 -0.6272896
H -2.7348819 2.3757792 1.0565923
C -4.0414786 0.2428312 -1.2559076
H -3.9195761 -1.9099269 -1.1502535
H -3.9875507 2.3879249 -1.0632251
H -4.6171332 0.2528047 -2.1762801

H	-0.6896010	1.1736140	2.0215977
H	-0.1618088	1.9424268	1.6209886

mTS6^{+} : ethylene addition to **1m**$^{+}$**.**THF
65
Energy = -1682.957632556

Ca	0.0450190	-0.4206145	-0.2472965
N	0.3009199	1.7102936	1.3352636
N	-0.6782436	-1.0342084	2.1742097
N	-1.3692601	1.5241049	-1.1680072
H	0.0135357	-1.1369039	-2.2554285
C	0.1781596	1.2498485	2.7409156
C	1.5870669	2.4237137	1.1826883
C	-0.8111025	2.6349740	1.0006320
C	-0.9290097	0.2214618	2.9258714
C	-1.9322890	-1.8269973	2.0711588
C	0.3407376	-1.8339115	2.8912873
C	-2.7867046	1.1865736	-0.8817385
C	-1.0001692	2.7972648	-0.5010277
C	-1.1750540	1.6733333	-2.6283920
H	-0.0059779	2.1004098	3.4164833
H	1.1424179	0.8208315	3.0284285
H	2.4077162	1.7494438	1.4315025
H	1.6324368	3.3021125	1.8452398
H	1.7130626	2.7554858	0.1510099
H	-1.7278047	2.2473496	1.4508580
H	-0.6325966	3.6239657	1.4517102
H	-1.8821529	0.6371457	2.5912314
H	-1.0396938	0.0028119	3.9996418
C	-2.9250647	-1.2373685	1.0737685
H	-1.6488618	-2.8365206	1.7609925
H	-2.4173503	-1.9154304	3.0561424
H	1.2936235	-1.2992094	2.9111879
H	0.4860117	-2.7830149	2.3748450
H	0.0309602	-2.0325536	3.9288087
C	-3.1203526	-0.2705034	-1.1736171
H	-2.9870457	1.4114803	0.1689812
H	-3.4551060	1.8309919	-1.4747731
H	-0.0708623	3.1463827	-0.9603104
H	-1.7600364	3.5726704	-0.6882347
H	-1.3773950	0.7237048	-3.1233218
H	-0.1332982	1.9369053	-2.8240594
H	-1.8268648	2.4634536	-3.0343572
N	-2.3842372	-1.2236567	-0.3055761
H	-3.1778483	-0.2122848	1.3560015
H	-3.8613709	-1.8157668	1.1183005
H	-4.2083415	-0.4149834	-1.0751562
H	-2.8493317	-0.5138425	-2.2042854
C	-2.4926128	-2.5719944	-0.9037622
H	-2.0137461	-2.5540402	-1.8843660
H	-3.5465719	-2.8736238	-1.0076760
H	-1.9818425	-3.3034734	-0.2754706
C	0.9398445	-2.7192440	-1.6725494
C	1.0093455	-2.9291977	-0.3189748
H	0.2090621	-3.2358144	-2.2808449
H	1.7883752	-2.3246214	-2.2192753
H	0.2527797	-3.5092972	0.2017157
H	1.8699438	-2.6119297	0.2651766
H	2.4336891	1.7806477	-2.0464649
C	2.5918836	0.6980219	-2.1219046
O	2.3441571	0.1185781	-0.7962240
C	4.0426545	0.3630297	-2.4466832
H	1.8562591	0.2576448	-2.8007087
C	3.6070373	-0.3387385	-0.2225102
C	4.6896747	0.3360169	-1.0532169
H	4.4928116	1.1006351	-3.1148881
H	4.1119662	-0.6228469	-2.9178809
H	3.6541082	-1.4302165	-0.3102815
H	3.6134093	-0.0602284	0.8346881
H	4.8702926	1.3543949	-0.6933339
H	5.6312321	-0.2172993	-1.0268651

mTS7^{+} : hydrogenolysis with H_2
67
Energy = -1684.157379838

Ca	0.4377507	-0.0346986	-0.1110306
N	1.3406886	0.5650240	2.1980165
N	-2.2199652	0.1196598	-0.1135824
N	-0.2924015	2.3771643	0.3704521
C	1.0260807	-0.5175238	3.1653521
C	2.8107895	0.7448579	2.1476608
C	0.6975848	1.8380443	2.6160537
C	-2.5783627	1.5602020	-0.1696220
C	-2.8261786	-0.5625277	-1.2781947
C	-2.7538788	-0.4784974	1.1333598
C	-1.7218761	2.4079475	0.7619880
C	0.5835322	2.8430172	1.4769555
C	-0.0844782	3.2737598	-0.7895317
C	-0.4025986	-1.0247707	3.0312990
H	1.1935551	-0.1752192	4.1985972
H	1.7333103	-1.3314397	2.9807530
H	3.2741064	-0.1830102	1.8070256
H	3.2073729	1.0071555	3.1403374
H	3.0582171	1.5343035	1.4366959
H	-0.2937954	1.6080450	3.0141312
H	1.2674194	2.2960608	3.4392676
H	-3.6419426	1.7051092	0.0772922
H	-2.4440634	1.8876811	-1.2042285
H	-2.4363177	-0.1171954	-2.1941162
H	-3.9232544	-0.4710413	-1.2637602
H	-2.5675157	-1.6238275	-1.2667744
C	-2.1054583	-1.8100351	1.4723425
H	-2.6014152	0.2321267	1.9485994
H	-3.8421301	-0.6265176	1.0473523
H	-1.8085319	2.0420318	1.7879084
H	-2.1004437	3.4420076	0.7622001
H	1.5691894	3.0196664	1.0370143
H	0.2199108	3.8020478	1.8795269
H	-0.7247961	2.9679510	-1.6193607
H	0.9588431	3.2000450	-1.1036441

H	-0.3264717	4.3155512	-0.5281974
N	-0.6449073	-1.7115519	1.7341606
H	-1.1000281	-0.1888836	3.1155362
H	-0.6263655	-1.7066216	3.8664537
H	-2.2356496	-2.5149664	0.6455395
H	-2.6199126	-2.2441960	2.3447156
C	-0.1343533	-3.0993050	1.8359048
H	0.9240116	-3.0960707	2.0955331
H	-0.2582377	-3.6067359	0.8768990
H	-0.6859211	-3.6568073	2.6084598
H	-1.0932676	-1.7032906	-3.4556525
C	-0.0469329	-1.5454514	-3.1716049
C	0.8620544	-1.4594611	-4.3889729
H	0.2526254	-2.3102176	-2.4506067
O	0.0550216	-0.2406404	-2.5131024
C	0.7082538	0.0139004	-4.7943134
H	0.5585344	-2.1542453	-5.1755359
H	1.8976520	-1.6752181	-4.1086531
C	0.6606451	0.7219193	-3.4449970
H	-0.2282790	0.1633822	-5.3412829
H	1.5335681	0.3753084	-5.4120128
H	1.6573139	0.9660925	-3.0666263
H	0.0357830	1.6174952	-3.4419564
H	2.4812727	-2.3792235	0.7723053
C	2.3327351	-2.3663655	-0.3148834
C	2.7721681	-1.0272614	-0.9178588
H	1.2630668	-2.5868817	-0.4861801
H	3.8098194	-0.8247823	-0.6145337
H	2.7778144	-1.0935604	-2.0161542
H	2.3760984	0.4865830	-0.7462125
H	2.0998683	1.3848070	-0.6835999
H	2.8642290	-3.2465572	-0.7116165

mTS8^{+} : anti-Markovnikov 1-hexene addition
77
Energy = -1840.308684854

Ca	-0.1413122	-0.1873858	0.3640573
N	1.9565861	0.5715373	1.8723206
N	0.0232925	-1.6816249	2.4967788
N	-0.3771587	2.2931107	1.0223127
H	-1.4241732	0.3134534	-1.2416225
C	2.1896597	-0.4853028	2.8873777
C	3.2158172	0.7904556	1.1296495
C	1.5611200	1.8406615	2.5323381
C	0.8986438	-1.0158554	3.4938444
C	-1.3525487	-1.8218151	3.0425450
C	0.5650881	-3.0264237	2.1975831
C	-1.4407219	2.3692071	2.0557056
C	0.9006858	2.8142947	1.5665897
C	-0.7656482	3.1052955	-0.1535637
H	2.8382007	-0.1134858	3.6970170
H	2.7342897	-1.2952827	2.3934270
H	3.5200192	-0.1390367	0.6472113
H	4.0199248	1.1206239	1.8058752
H	3.0723976	1.5507806	0.3605793
H	0.8751957	1.6052033	3.3490493
H	2.4403145	2.3228455	2.9884937
H	0.3380875	-0.1968458	3.9498658
H	1.1473140	-1.7175606	4.3056903
C	-2.1146819	-0.5003551	3.0671028
H	-1.8782263	-2.5427226	2.4098567
H	-1.3252065	-2.2446685	4.0592787
H	1.5548841	-2.9397290	1.7425984
H	-0.0952810	-3.5367490	1.4959230
H	0.6544059	-3.6311384	3.1131831
C	-2.6432580	1.4928833	1.7342568
H	-1.0089214	2.0735458	3.0152614
H	-1.7802052	3.4109254	2.1713561
H	1.5619694	3.0063715	0.7165777
H	0.7452108	3.7795302	2.0744647
H	-1.6770825	2.7001456	-0.5933027
H	0.0241104	3.0448188	-0.9056678
H	-0.9138746	4.1602723	0.1279698
N	-2.3233947	0.0435795	1.7051900
H	-1.5652707	0.2393169	3.6548934
H	-3.0792958	-0.6531881	3.5765159
H	-3.4428134	1.7010326	2.4636047
H	-3.0294392	1.7444211	0.7428628
C	-3.4494743	-0.6567186	1.0518143
H	-3.5435792	-0.2796366	0.0321039
H	-4.3898349	-0.4867030	1.5993822
H	-3.2570783	-1.7304046	1.0156556
C	-1.6120110	-1.5736463	-1.7421669
C	-0.9993701	-2.4219574	-0.8513432
H	-2.6713162	-1.3762775	-1.6091248
H	-1.5464593	-2.8343105	-0.0076572
H	-0.0078308	-2.8284819	-1.0429752
H	2.1622705	1.6720149	-1.8028851
C	1.7057852	0.8082242	-2.3003811
C	2.5952883	0.2667473	-3.4147753
H	0.6874845	1.0566642	-2.6094670
O	1.6059685	-0.2725143	-1.3151577
C	3.4943628	-0.7299324	-2.6660912
H	3.1560321	1.0610962	-3.9121227
H	1.9946299	-0.2569850	-4.1649562
C	2.5129985	-1.3544724	-1.6847366
H	4.2908724	-0.2035115	-2.1298845
H	3.9474768	-1.4747091	-3.3241730
H	1.9283683	-2.1561735	-2.1503911
H	2.9718373	-1.7320645	-0.7669752
C	-1.1034360	-1.3556710	-3.1485589
C	-1.7200143	-0.1767243	-3.9037376
H	-1.3250945	-2.2786256	-3.7081357
H	-0.0109986	-1.2706251	-3.1368053
C	-1.2053329	-0.0534656	-5.3414356
H	-2.8133435	-0.2934002	-3.9187873
H	-1.5174731	0.7435188	-3.3429640
C	-1.8133101	1.1392532	-6.0862598
H	-0.1107826	0.0464752	-5.3267618
H	-1.4213340	-0.9797601	-5.8912856

H	-1.4269554	1.2132089	-7.1087121
H	-2.9045145	1.0482652	-6.1435179
H	-1.5844632	2.0794091	-5.5699887

mTS9$^+$: hdrogenolysis with H_2
79
Energy = -1841.507891323

Ca	0.2345697	0.5059643	-0.1286292
N	1.2520528	1.0869170	2.1346441
N	-1.6117678	2.3229815	-0.7807842
N	1.3214300	2.8226483	-0.3724735
C	0.2657988	0.8360411	3.2167107
C	2.4538609	0.2571217	2.3847395
C	1.6359892	2.5226298	2.1024808
C	-0.8972298	3.5453538	-1.2294089
C	-2.4951130	1.8643164	-1.8753859
C	-2.4358675	2.6384757	0.4105181
C	0.2880671	3.8842169	-0.3351409
C	2.2586277	2.9379992	0.7751072
C	2.1041399	2.9470228	-1.6232982
C	-1.1249558	1.3511408	2.8737063
H	0.5998372	1.2944919	4.1606762
H	0.2356641	-0.2452468	3.3807538
H	2.1680913	-0.7965735	2.3813778
H	2.9085226	0.5082040	3.3551511
H	3.1821398	0.4187835	1.5888227
H	0.7439493	3.1212042	2.3018221
H	2.3482597	2.7406589	2.9133398
H	-1.5851357	4.4047532	-1.2675028
H	-0.5558809	3.3651801	-2.2525099
H	-1.8876391	1.6369383	-2.7521748
H	-3.2379048	2.6346749	-2.1342016
H	-3.0264819	0.9587376	-1.5735706
C	-2.8717291	1.3979602	1.1714485
H	-1.8586688	3.2963270	1.0638680
H	-3.3332005	3.2035958	0.1119582
H	-0.0451809	4.0080207	0.6980823
H	0.7142139	4.8503868	-0.6476551
H	3.1101274	2.2911912	0.5452172
H	2.6363270	3.9692952	0.8636538
H	1.4428903	2.8692519	-2.4888886
H	2.8303405	2.1324402	-1.6615178
H	2.6232661	3.9170049	-1.6668731
N	-1.7399247	0.6164469	1.7361163
H	-1.0725845	2.4102573	2.6133610
H	-1.7692442	1.2775703	3.7636518
H	-3.4258867	0.7252866	0.5099075
H	-3.5651220	1.6981198	1.9735697
C	-2.3096463	-0.6521189	2.2485570
H	-1.5365519	-1.2442588	2.7380902
H	-2.7206810	-1.2308146	1.4186094
H	-3.1117037	-0.4492354	2.9747245
H	-2.0001380	-0.7434748	-3.2936721
C	-1.1642230	-1.2385710	-2.7866889
C	-0.4248183	-2.1872676	-3.7173581
H	-1.5243062	-1.7065607	-1.8674279
O	-0.1824064	-0.2192910	-2.4141855
C	0.5037040	-1.2236097	-4.4712754
H	-1.1065994	-2.7292427	-4.3769300
H	0.1580240	-2.9114035	-3.1397143
C	0.9285098	-0.2482648	-3.3773392
H	-0.0491802	-0.7021265	-5.2592137
H	1.3634308	-1.7236688	-4.9228589
H	1.8190470	-0.5850809	-2.8396961
H	1.0796270	0.7720861	-3.7370320
H	0.0853395	-2.1111285	1.6451512
C	0.0112525	-2.3352189	0.5703076
C	1.3071353	-1.9214168	-0.1245190
H	-0.8541374	-1.7370897	0.2104209
H	2.1654840	-2.3393626	0.4184148
H	1.3371336	-2.3460845	-1.1402491
H	2.0383167	-0.5525118	-0.4088330
H	2.4321827	0.2774374	-0.6148996
C	-0.4191617	-3.8100624	0.4172355
C	0.5688265	-4.7952881	1.0458958
H	-0.5223193	-4.0335425	-0.6543430
H	-1.4125118	-3.9553257	0.8682395
C	0.1544856	-6.2605718	0.8714314
H	1.5618574	-4.6435250	0.6030591
H	0.6687788	-4.5708684	2.1184906
C	1.1515833	-7.2388642	1.5012744
H	-0.8396676	-6.4124440	1.3139299
H	0.0530274	-6.4791757	-0.2006146
H	0.8358436	-8.2784497	1.3603946
H	2.1461418	-7.1247881	1.0536500
H	1.2480211	-7.0589921	2.5787513

THF : tetrahydrofuran
13
Energy = -232.5925783441

O	-0.0015557	0.0014218	-1.2568307
C	0.1578025	1.1738437	-0.4164078
C	-0.1578691	-1.1728329	-0.4183286
C	0.2220753	-0.7358684	0.9960709
C	-0.2222255	0.7348547	0.9972812
H	1.2051482	1.5036723	-0.4640137
H	-0.4821435	1.9672156	-0.8154063
H	-1.2045003	-1.5048433	-0.4660472
H	0.4835715	-1.9641002	-0.8191326
H	-0.2703177	-1.3371085	1.7652312
H	1.3060902	-0.8054752	1.1400205
H	-1.3062581	0.8043259	1.1412623
H	0.2701820	1.3348946	1.7673744

TS1a^{2+} : Markovnikov addition to **1**$^{2+}$**.**THF
112
Energy = -3055.670880147

Ca	0.1142991	-0.2071842	1.7797610
Ca	-0.6578101	-0.0098052	-1.7743499
H	-0.4112699	1.0261177	0.1030092

N	2.0047306	-0.6823819	3.5477250
N	-0.4317848	-1.6992938	-3.8794055
N	-2.1049699	0.3081982	2.8625701
N	-2.2112324	1.9809049	-2.3299977
N	0.4339477	1.9124953	3.1665638
N	0.3502430	1.2586476	-3.7712666
N	-0.5791783	-2.2370772	3.1938192
N	-2.9584497	-0.9524132	-2.3670611
C	-2.0495162	1.7087254	3.3562729
C	-1.4676406	2.8934719	-3.2369585
C	1.7288891	-2.0194726	4.1455047
C	-1.7266948	-2.4223806	-3.9833609
C	-0.7424487	2.0216330	4.0704535
C	-0.6998003	2.1549130	-4.3230087
C	0.2545633	-2.2712755	4.4230179
C	-2.9369360	-1.5347791	-3.7335905
C	3.4223819	-0.6774329	3.1137393
C	0.6483805	-2.7135187	-3.8956464
C	-3.1834084	0.2020064	1.8597090
C	-2.6173419	2.7592586	-1.1382060
C	1.8233890	0.3837763	4.5719194
C	-0.2500797	-0.8041277	-5.0531409
C	-2.3516962	-0.6494007	3.9687501
C	-3.4187044	1.4312833	-2.9978727
C	1.6834872	1.7711793	3.9592320
C	0.7857172	0.2828289	-4.8048983
C	-2.0117072	-2.0772722	3.5597251
C	-3.9325688	0.1709911	-2.3094320
C	0.5184732	3.1330682	2.3309895
C	1.5168624	2.0703372	-3.3594300
C	-0.4211457	-3.5038825	2.4421452
C	-3.3622287	-1.9967178	-1.3983047
H	-2.8940235	1.9171202	4.0301941
H	-2.1528048	3.6152033	-3.7079900
H	2.2922745	-2.1417170	5.0833733
H	-1.8305220	-2.8875264	-4.9763621
H	-2.1636540	2.3584892	2.4840559
H	-0.7782362	3.4703106	-2.6138069
H	2.1136734	-2.7667544	3.4451933
H	-1.6989856	-3.2372731	-3.2544124
H	3.5838690	-1.4895130	2.4028039
H	0.5336883	-3.3866475	-3.0443584
H	-2.9167082	0.8194261	1.0005013
H	-1.7273885	3.0310951	-0.5681987
H	0.9349310	0.1475727	5.1615913
H	-1.2132980	-0.3508766	-5.2966946
H	-1.7448677	-0.3472435	4.8258002
H	-3.1689799	1.2134214	-4.0389656
H	-0.6019752	1.3345508	4.9081441
H	-1.3874544	1.5522601	-4.9204108
H	-0.1320374	-1.5212058	5.1167274
H	-2.9563592	-0.7145887	-4.4543716
H	4.0916792	-0.8267664	3.9740983
H	0.6110697	-3.3069547	-4.8216495
H	-4.1497125	0.5297738	2.2711493
H	-3.1717147	3.6638821	-1.4308310
H	2.6740304	0.3793270	5.2694285
H	0.0488056	-1.3932160	-5.9333128
H	-3.4027200	-0.6044652	4.2919574
H	-4.2228730	2.1824565	-3.0184453
H	-0.7972008	3.0337482	4.4986505
H	-0.2479486	2.8894427	-5.0069777
H	0.1507747	-3.2452808	4.9248955
H	-3.8502791	-2.1251477	-3.9047028
H	3.6752189	0.2678778	2.6309762
H	1.6242384	-2.2303202	-3.8291858
H	-3.2796382	-0.8334520	1.5307823
H	-3.2630742	2.1526146	-0.5022592
H	-0.3775615	3.2202933	1.7154138
H	1.2316801	2.7595642	-2.5614770
H	0.6338139	-3.6785881	2.2209930
H	-2.6492412	-2.8231125	-1.4199458
H	2.5227980	1.9732618	3.2866333
H	1.7232290	-0.1626462	-4.4578593
H	-2.6018584	-2.3703919	2.6870930
H	-4.1299208	0.3749873	-1.2527864
H	1.7248533	2.5258084	4.7593090
H	1.0072685	0.7976560	-5.7523539
H	-2.2824650	-2.7655333	4.3744013
H	-4.8913451	-0.1251493	-2.7615701
H	1.3793644	3.0602258	1.6649272
H	2.2993117	1.4077604	-2.9857929
H	-0.9699873	-3.4293844	1.4997822
H	-3.3614646	-1.5720303	-0.3941327
H	0.6214281	4.0312004	2.9580356
H	1.9145422	2.6533261	-4.2035163
H	-0.7998850	-4.3593132	3.0205347
H	-4.3660140	-2.3858028	-1.6266390
C	1.7720054	-0.9452656	-1.1001090
C	0.9393457	-1.8981687	-0.5027613
H	1.7684375	-0.8704258	-2.1888812
H	1.2499318	-2.2952751	0.4692613
H	-0.4764241	-1.4370588	-0.0059473
C	2.9634801	-0.3267560	-0.4073404
C	4.2231399	-0.4237160	-1.2910269
C	2.7952360	1.1348725	0.0653042
H	3.1666019	-0.9298667	0.4892488
C	5.4884485	-0.0087507	-0.5303366
H	4.0944912	0.2321742	-2.1635989
H	4.3228812	-1.4461264	-1.6702935
C	4.0828296	1.7886753	0.4892822
H	2.2960754	1.7264356	-0.7112753
H	2.0909708	1.1711251	0.9123014
C	5.2887927	1.2655774	0.2431022
H	6.3269927	0.1138555	-1.2278323
H	5.7928926	-0.8114095	0.1588960
H	4.0073657	2.7332711	1.0283286
H	6.1800672	1.7785387	0.6012964
H	0.4824634	-2.6820909	-1.1018379

TS1^{2+}.THF : anti-Markovnikov to **1^{2+}.**(THF)$_2$
125
Energy = -3288.275872287

Ca	-2.2727208	-0.0212394	-0.3825623
H	-0.1925516	-0.0492754	-1.0690495
N	-2.7374572	-2.4131172	-1.2099099
N	-3.9165116	-1.1669439	1.2428306
N	-4.4371296	1.3982335	-0.3297112
N	-3.2341023	0.1485321	-2.7539335
C	-3.9874557	-2.8894380	-0.5642118
H	-4.1236884	-3.9667184	-0.7419313
H	-4.8346127	-2.3825997	-1.0319370
C	-3.9821607	-2.6211160	0.9324563
H	-4.8744955	-3.0718501	1.3910683
H	-3.1128866	-3.0925513	1.3992106
C	-5.2547835	-0.5471777	1.0345539
H	-5.9148537	-0.7894692	1.8803776
H	-5.7051879	-1.0005080	0.1489358
C	-5.1932665	0.9654949	0.8753937
H	-6.2196972	1.3626388	0.8521813
H	-4.6979532	1.4147566	1.7411078
C	-5.2019186	1.1077030	-1.5690736
H	-6.0369999	1.8154775	-1.6805556
H	-5.6422467	0.1117996	-1.4790813
C	-4.3130937	1.1712996	-2.8029204
H	-4.9266631	1.0480480	-3.7072991
H	-3.8341946	2.1514833	-2.8744903
C	-3.7883697	-1.1807929	-3.1250894
H	-3.9313324	-1.2365735	-4.2145582
H	-4.7792504	-1.2739263	-2.6748307
C	-2.9017735	-2.3357924	-2.6849941
H	-3.3172196	-3.2763871	-3.0777541
H	-1.9016982	-2.2252367	-3.1149337
C	-1.6478527	-3.3706469	-0.9157576
H	-1.5033396	-3.4614858	0.1620731
H	-0.7228224	-3.0011171	-1.3580558
H	-1.8751250	-4.3653057	-1.3269213
C	-3.5224515	-1.0160834	2.6629232
H	-3.5644526	0.0343594	2.9514249
H	-2.5020462	-1.3846736	2.7977321
H	-4.1949317	-1.5837573	3.3220634
C	-4.2043930	2.8575440	-0.2337184
H	-3.6097044	3.2005795	-1.0830975
H	-3.6589849	3.0731311	0.6851048
H	-5.1555988	3.4095250	-0.2275351
C	-2.1624809	0.5216180	-3.7033577
H	-1.3395482	-0.1919015	-3.6296465
H	-1.7859892	1.5139728	-3.4452334
H	-2.5333744	0.5414955	-4.7385648
Ca	2.0505114	-0.0242927	-0.4271151
H	1.3416793	0.7310403	1.4213808
N	4.1226732	1.5025190	0.0947848
N	2.2972663	1.9050921	-2.2369350
N	2.3926204	-1.1116272	-2.7478399
N	4.2780043	-1.5140983	-0.4419943
C	4.4740721	2.3281946	-1.0864063
H	5.1629683	3.1371730	-0.7959266
H	5.0133680	1.7004698	-1.7993802
C	3.2497959	2.9355895	-1.7544106
H	3.5753667	3.5891563	-2.5793695
H	2.7091306	3.5659940	-1.0425612
C	2.8224522	1.2469178	-3.4585217
H	2.7188876	1.9201746	-4.3243370
H	3.8925747	1.0726007	-3.3244627
C	2.1164713	-0.0674578	-3.7638997
H	2.4075740	-0.4129326	-4.7685628
H	1.0336807	0.0873861	-3.7732201
C	3.7660643	-1.6472855	-2.8986286
H	3.8184689	-2.3373063	-3.7556711
H	4.4350110	-0.8147401	-3.1272446
C	4.2454184	-2.3766165	-1.6513580
H	5.2406076	-2.8058357	-1.8497431
H	3.5778481	-3.2136633	-1.4277538
C	5.4085056	-0.5534944	-0.5217938
H	6.3583683	-1.0717806	-0.3177675
H	5.4720401	-0.1789093	-1.5459281
C	5.2574326	0.6126216	0.4471701
H	6.2012099	1.1801188	0.4793658
H	5.0778522	0.2385925	1.4597761
C	3.8356486	2.3922886	1.2426318
H	3.0029858	3.0550385	0.9992257
H	3.5356936	1.7893733	2.1003301
H	4.7172541	3.0020877	1.4976672
C	1.0097888	2.5541338	-2.5539457
H	0.2605135	1.7908342	-2.7639206
H	0.6788007	3.1310174	-1.6900839
H	1.1032510	3.2337425	-3.4156411
C	1.4042676	-2.1967095	-2.8959779
H	1.5912874	-2.9780299	-2.1555150
H	0.4129146	-1.7732311	-2.7156772
H	1.4425652	-2.6480341	-3.8996347
C	4.4858241	-2.3898125	0.7328497
H	4.4753914	-1.7988484	1.6495303
H	3.6825654	-3.1256598	0.7824232
H	5.4501800	-2.9167973	0.6623219
O	-1.6160599	1.7180056	1.1745447
C	-1.6450743	1.9525462	2.6226329
C	-0.6105515	3.0392067	2.8871388
C	-0.6398862	3.8451555	1.5816636
C	-0.7703809	2.7398958	0.5478665
H	-1.4180930	1.0089847	3.1214445
H	-2.6580970	2.2747570	2.8885786
H	0.3763718	2.5862603	3.0200726
H	-0.8601745	3.6334371	3.7686981
H	-1.5051505	4.5156147	1.5524723
H	0.2646042	4.4394304	1.4288179
H	-1.2628366	3.0528111	-0.3773105
H	0.1942383	2.2641576	0.3409078
C	0.9969228	-1.9266524	1.2259748
C	0.6363537	-0.8049050	1.9491718

H	1.8244847	-2.5545613	1.5350956
H	0.4182453	-2.2516446	0.3691003
H	-0.3195690	-0.3437976	1.7089715
C	1.0833203	-0.5763837	3.3794523
C	0.2939365	-1.4648509	4.3656510
C	2.5833497	-0.8065075	3.5824827
H	0.8806780	0.4714323	3.6323793
C	0.8666118	-1.4018930	5.7578950
H	0.2873209	-2.5019234	4.0007263
H	-0.7591077	-1.1502459	4.3945279
C	3.0175848	-0.3765067	4.9871628
H	2.8132210	-1.8699420	3.4419672
H	3.1301842	-0.2424167	2.8162733
C	2.0736773	-0.8982128	6.0393347
H	0.2434749	-1.7840489	6.5658558
H	4.0351099	-0.7309714	5.1974699
H	3.0659976	0.7220324	5.0478288
H	2.4120514	-0.8614855	7.0740236

$TS1^{2+}$: anti-Markovnikov to **1^{2+}.THF**
112
Energy = -3055.677829693

Ca	-0.0286213	0.1838504	1.9555805
Ca	-0.0366771	0.6045556	-1.5771248
H	0.0090470	1.5917477	0.3501739
N	1.4535573	-1.3903069	3.4816539
N	0.9940156	-0.5290208	-3.8363436
N	-1.4912126	1.7340843	3.3085161
N	-1.0918798	2.8796848	-2.1257824
N	1.5242931	1.6391037	3.3436759
N	1.7959763	2.1156059	-2.5137383
N	-1.5974592	-1.2525097	3.4054941
N	-1.9050321	0.2600213	-3.3098301
C	-0.6268056	2.8445232	3.7868597
C	0.0196755	3.8569530	-2.2377214
C	0.5338174	-2.4245615	4.0307151
C	-0.1720301	-1.0202815	-4.6183591
C	0.7013035	2.3574760	4.3523860
C	1.1787149	3.3446129	-3.0786883
C	-0.8057432	-1.8748026	4.4987619
C	-1.3499818	-0.0572534	-4.6518192
C	2.6203971	-2.1019544	2.9058330
C	1.9915243	-1.6255697	-3.8015782
C	-2.5774983	2.2974706	2.4777112
C	-1.9972140	3.3391621	-1.0522521
C	1.9369265	-0.4922038	4.5663281
C	1.6241935	0.6400824	-4.5056236
C	-2.0696910	0.9714347	4.4418426
C	-1.8442100	2.7518190	-3.3957403
C	2.5420859	0.7931784	4.0190282
C	2.5547513	1.4012784	-3.5709501
C	-2.6411637	-0.3647305	3.9858209
C	-2.7176930	1.5039229	-3.3945381
C	2.2004088	2.6134083	2.4578239
C	2.7044365	2.4824838	-1.4043743
C	-2.2599774	-2.3096146	2.6083667
C	-2.7657362	-0.8512294	-2.8476618
H	-1.1463295	3.4433515	4.5505252
H	-0.3368683	4.8104933	-2.6580078
H	1.0111765	-2.9520894	4.8710955
H	0.1245495	-1.2382289	-5.6568229
H	-0.4483834	3.4989584	2.9287291
H	0.3605479	4.0579471	-1.2179851
H	0.3815781	-3.1665015	3.2396057
H	-0.4739268	-1.9736936	-4.1716837
H	2.2977666	-2.7223877	2.0681114
H	1.5308635	-2.5296123	-3.4025962
H	-2.1318188	2.8605950	1.6591782
H	-1.4094770	3.4355798	-0.1397090
H	1.0943126	-0.2518621	5.2186970
H	0.8348204	1.3081965	-4.8566086
H	-1.2845238	0.8088024	5.1847513
H	-1.1284672	2.7052584	-4.2206756
H	0.5268694	1.6824591	5.1935923
H	0.8387211	3.1234852	-4.0929784
H	-0.6540421	-1.1248868	5.2782937
H	-1.0544685	0.8817712	-5.1246142
H	3.0968377	-2.7393893	3.6651780
H	2.3635938	-1.8415003	-4.8143541
H	-3.2280346	2.9605033	3.0672866
H	-2.4592941	4.3051069	-1.3082023
H	2.6794273	-1.0148710	5.1877991
H	2.1798385	0.3101782	-5.3965164
H	-2.8603706	1.5561839	4.9363019
H	-2.4725824	3.6394685	-3.5677834
H	1.2577269	3.2194079	4.7504529
H	1.9353362	4.1390656	-3.1678318
H	-1.3788480	-2.6930274	4.9602162
H	-2.1355568	-0.4921281	-5.2883839
H	3.3568099	-1.3856017	2.5395133
H	2.8392553	-1.3529443	-3.1703127
H	-3.1862155	1.4957395	2.0537818
H	-2.7879381	2.6060685	-0.8783415
H	1.4550928	3.2181825	1.9387412
H	2.1352779	2.9852253	-0.6211098
H	-1.5137491	-3.0112816	2.2329617
H	-2.1871979	-1.7759530	-2.8246223
H	3.3171587	0.5628202	3.2827386
H	3.2440933	0.7117382	-3.0754624
H	-3.4009701	-0.2074514	3.2159858
H	-3.3890148	1.5164465	-2.5321543
H	3.0304101	1.3456244	4.8355486
H	3.1681505	2.1050719	-4.1535973
H	-3.1413620	-0.8585890	4.8320133
H	-3.3514627	1.4909205	-4.2934759
H	2.7807415	2.0705199	1.7083456
H	3.1411033	1.5744633	-0.9827610
H	-2.7675571	-1.8550228	1.7548315
H	-3.1210386	-0.6402574	-1.8358459
H	2.8728626	3.2707280	3.0292132

H	3.5113440	3.1447838	-1.7528715
H	-2.9888801	-2.8672296	3.2147381
H	-3.6296812	-0.9949005	-3.5131341
C	1.7307979	-0.8897896	-0.1252165
C	0.6090946	-1.6912218	0.0534871
H	2.3057660	-0.4888001	0.7033951
H	2.1978422	-0.7996434	-1.1032343
H	0.4014369	-2.0392564	1.0727165
H	-0.8451784	-0.8077199	0.0350434
C	0.2070832	-2.7364066	-0.9723839
C	1.3166143	-3.8047895	-1.0547329
C	-1.1266441	-3.4318283	-0.6881646
H	0.1397347	-2.2535232	-1.9600077
C	0.9258134	-4.9416361	-1.9612721
H	1.5269323	-4.1800014	-0.0408608
H	2.2515640	-3.3457949	-1.3938596
C	-1.5101759	-4.3826225	-1.8306358
H	-1.0204627	-4.0148626	0.2360264
H	-1.9098393	-2.6864939	-0.5167609
C	-0.3372503	-5.1847169	-2.3269823
H	1.7289303	-5.5763161	-2.3320434
H	-2.3050916	-5.0622027	-1.4983491
H	-1.9461848	-3.8231698	-2.6725209
H	-0.5527470	-6.0050356	-3.0097444

TS2a^{2+} : hydrogenolysis with H_2

114

Energy = -3056.873269707

Ca	-0.0557780	-0.0580217	1.7314096
Ca	-0.4674472	-0.0703040	-1.9149159
H	-1.3580234	-0.4263587	-0.0094190
N	1.1482706	1.5942862	3.3719045
N	1.1817149	-0.7488182	-3.8790410
N	-2.2058487	-0.8998809	2.7342820
N	-2.8113975	0.5738304	-2.8094224
N	-1.5228839	2.0130768	2.0888947
N	-0.2166814	1.9468478	-3.4504830
N	0.4554334	-1.3008458	4.1019084
N	-1.4169219	-2.0994582	-3.1347195
C	-3.1892348	0.1915877	2.5091659
C	-2.7256795	1.9432006	-3.3818310
C	1.7072350	0.8311225	4.5201323
C	0.7912621	-2.1159250	-4.3187826
C	-2.6646619	1.5613329	2.9267402
C	-1.4736938	2.1557157	-4.2214293
C	0.7298163	-0.2010144	5.0658672
C	-0.7113076	-2.3195296	-4.4244557
C	2.2407084	2.3546409	2.7192189
C	2.6466549	-0.7685953	-3.6561946
C	-2.6694941	-2.1093834	2.0153965
C	-3.9079240	0.5407137	-1.8139293
C	0.1278449	2.5543298	3.8731841
C	0.8820190	0.2582768	-4.9323490
C	-2.0595407	-1.1982309	4.1785038
C	-3.1072772	-0.4246149	-3.8695993
C	-0.7595295	3.0913934	2.7643830
C	0.9089780	1.6813334	-4.3883686
C	-0.8033021	-2.0037316	4.4754028
C	-2.8601020	-1.8484068	-3.3917238
C	-2.0336140	2.5321329	0.8056167
C	0.0878989	3.1652459	-2.6631767
C	1.5530134	-2.2914522	4.2129856
C	-1.2795957	-3.3000733	-2.2791802
H	-4.1271171	-0.0154422	3.0476505
H	-3.6097363	2.1681026	-3.9981469
H	1.9980768	1.5169092	5.3307237
H	1.2497431	-2.3511713	-5.2918905
H	-3.4123758	0.1884663	1.4384596
H	-2.7371225	2.6429849	-2.5407273
H	2.6225236	0.3399339	4.1767061
H	1.2165165	-2.8136389	-3.5903549
H	3.0265011	1.6696869	2.3949557
H	2.8880755	-1.4748056	-2.8594822
H	-2.7196819	-1.8716999	0.9509177
H	-3.7847042	1.3673574	-1.1148409
H	-0.4842914	2.0471692	4.6218957
H	-0.1038561	0.0415963	-5.3501407
H	-2.0381102	-0.2533652	4.7263283
H	-2.4771520	-0.2101003	-4.7361412
H	-2.3382273	1.5396887	3.9691088
H	-1.4700564	1.4697559	-5.0711764
H	-0.2170887	0.2792569	5.3204343
H	-1.1342615	-1.6374142	-5.1650209
H	2.6775433	3.0907522	3.4103739
H	3.1744644	-1.0699282	-4.5729950
H	-3.6540247	-2.4342670	2.3842706
H	-4.8868601	0.6445817	-2.3052334
H	0.6221869	3.3942973	4.3845507
H	1.6023219	0.1687114	-5.7592286
H	-2.9345371	-1.7556789	4.5469950
H	-4.1515602	-0.3248046	-4.2025667
H	-3.4864421	2.2914844	2.8702438
H	-1.4928732	3.1724938	-4.6402719
H	1.1303431	-0.6149741	6.0034554
H	-0.9051076	-3.3383859	-4.7917242
H	1.8557818	2.8801423	1.8424906
H	2.9950756	0.2229688	-3.3662841
H	-1.9570247	-2.9253171	2.1573008
H	-3.8819286	-0.3947044	-1.2531114
H	-2.5332522	1.7233245	0.2752032
H	-0.7502355	3.3974593	-2.0028998
H	2.5145541	-1.8043061	4.0525923
H	-0.2229107	-3.5257688	-2.1211096
H	-0.1520341	3.5826057	1.9992203
H	1.8391943	1.8611048	-3.8423969
H	-0.8195363	-2.9457001	3.9197525
H	-3.3982397	-2.0338255	-2.4588484
H	-1.4370473	3.8544774	3.1771452
H	0.8868607	2.3947174	-5.2251042
H	-0.7902438	-2.2649408	5.5450959

H	-3.2482265	-2.5601498	-4.1349405
H	-1.1874830	2.8665303	0.2012999
H	0.9766865	2.9915388	-2.0515615
H	1.4226553	-3.0767043	3.4650169
H	-1.7486961	-3.1042087	-1.3114982
H	-2.7309053	3.3702467	0.9594836
H	0.2632641	4.0287845	-3.3210867
H	1.5573232	-2.7521464	5.2122373
H	-1.7583321	-4.1753172	-2.7416053
C	1.5955143	-1.1041636	-0.1967777
C	1.0596799	-2.3986191	0.4358352
H	1.7116809	-3.2719603	0.2685320
H	1.5255456	-1.2547946	-1.2911698
H	0.9577139	-2.3402343	1.5339094
H	0.0656751	-2.6457903	0.0525584
C	3.0964764	-0.8723607	0.0905547
C	3.5519812	0.5373045	-0.3354480
C	3.4262536	-1.0968975	1.5703630
H	3.7053515	-1.6037961	-0.4719531
C	4.8911019	0.9394889	0.2193255
H	2.8019952	1.2791493	-0.0264842
H	3.5829626	0.6044634	-1.4293050
C	4.9140503	-0.9127734	1.8933138
H	2.8323928	-0.3904876	2.1732916
H	3.1195299	-2.1008187	1.8718234
C	5.5054853	0.2903784	1.2131378
H	5.3646340	1.8145735	-0.2241685
H	5.0558111	-0.8314480	2.9796216
H	5.4693544	-1.8138191	1.5934580
H	6.4814745	0.6285361	1.5577650
H	0.9472259	0.4310180	-0.1497779
H	0.5332157	1.2639293	-0.1683342

TS2^{2+} : hydrogenolysis with H_2

114

Energy = -3056.883245801

Ca	0.0299772	0.4989441	1.6922408
Ca	0.3673031	0.5058737	-1.8815441
H	-0.7872819	1.1534220	-0.1838128
N	1.9331440	-0.1492065	3.2409649
N	1.0897054	-1.3516691	-3.5376295
N	-1.4290202	2.3469753	2.6772205
N	-0.6223871	2.5582689	-3.0301239
N	1.5037465	2.5701367	1.9967120
N	2.1549934	1.4968469	-3.4294140
N	-1.0291299	-0.3169836	4.0048885
N	-1.6915427	-0.2836473	-3.0948646
C	-0.6865403	3.6246742	2.5294027
C	0.4724129	3.3350124	-3.6677555
C	1.3753306	-0.8814237	4.4102087
C	-0.1485489	-2.0410352	-3.9905707
C	0.7800930	3.4806945	2.9197774
C	1.4930787	2.4465051	-4.3642131
C	0.1112388	-0.2298996	4.9567820
C	-1.2973797	-1.0824437	-4.2836460
C	2.9030164	-1.0220030	2.5391657
C	2.0308257	-2.3694039	-3.0124521
C	-2.6890542	2.4177214	1.9021905
C	-1.3559548	3.4480515	-2.1021732
C	2.6125169	1.0944515	3.6949728
C	1.7352348	-0.6345526	-4.6688130
C	-1.7268837	2.0988122	4.1087031
C	-1.5602951	2.0275652	-4.0540923
C	2.7928572	2.1101971	2.5756480
C	2.7695433	0.3784732	-4.1952224
C	-2.0957993	0.6505138	4.3829453
C	-2.4566015	0.9255682	-3.5018292
C	1.7907662	3.2883865	0.7377204
C	3.2176940	2.2012530	-2.6754590
C	-1.6272565	-1.6689717	4.1034723
C	-2.5309774	-1.0955206	-2.1853413
H	-1.1522942	4.4174290	3.1340321
H	0.0694449	4.0586106	-4.3926784
H	2.1243399	-0.9542025	5.2128821
H	0.0488154	-2.6483346	-4.8870392
H	-0.7695680	3.9283885	1.4825600
H	0.9567592	3.9152153	-2.8764743
H	1.1613002	-1.9031616	4.0824647
H	-0.4328391	-2.7349750	-3.1928032
H	2.3931084	-1.9262902	2.1989592
H	1.5430484	-2.9341588	-2.2166315
H	-2.4417099	2.5362501	0.8457740
H	-0.6653223	3.8203940	-1.3430481
H	2.0218605	1.5301686	4.5039411
H	0.9570664	-0.1291992	-5.2460809
H	-0.8497660	2.3822729	4.6951019
H	-0.9728192	1.6449587	-4.8929249
H	0.8603469	3.0820827	3.9341073
H	1.0079950	1.8660784	-5.1522899
H	0.3005248	0.8242350	5.1722314
H	-1.0158986	-0.3897726	-5.0803775
H	3.7323932	-1.3081025	3.2023130
H	2.3442435	-3.0621022	-3.8070380
H	-3.3197807	3.2524318	2.2426931
H	-1.7959019	4.3019082	-2.6392753
H	3.5978378	0.8518762	4.1203019
H	2.2167389	-1.3538284	-5.3483449
H	-2.5519423	2.7451535	4.4456402
H	-2.1890576	2.8389324	-4.4514919
H	1.2556927	4.4730979	2.9302526
H	2.2458180	3.0792981	-4.8575340
H	-0.1570083	-0.7033841	5.9132557
H	-2.1555518	-1.6599028	-4.6590095
H	3.3055755	-0.5011270	1.6682282
H	2.9177662	-1.8853391	-2.5981701
H	-3.2503716	1.4875000	2.0196280
H	-2.1434274	2.8897113	-1.5961822
H	0.8567045	3.5863019	0.2586317
H	2.7957417	3.0665278	-2.1604091
H	-0.8696693	-2.4333908	3.9266687

H	-2.0061615	-2.0150472	-1.9188224
H	3.3624322	1.6639949	1.7547812
H	3.5003612	-0.1039577	-3.5405070
H	-2.9896680	0.3749842	3.8155981
H	-2.9868003	1.2825465	-2.6151446
H	3.3785747	2.9619541	2.9547704
H	3.3240426	0.7658446	-5.0629166
H	-2.3492356	0.5417009	5.4489587
H	-3.2186923	0.6665248	-4.2516616
H	2.3252111	2.6119625	0.0692568
H	3.6470050	1.5227635	-1.9335460
H	-2.4149939	-1.7740634	3.3553965
H	-2.7173324	-0.5245721	-1.2721281
H	2.4009793	4.1856049	0.9221113
H	4.0151855	2.5526979	-3.3462983
H	-2.0572093	-1.8317534	5.1029984
H	-3.4887145	-1.3607490	-2.6562582
C	0.4461261	-1.6227635	-0.0623646
C	-0.6220832	-2.1790138	0.8809672
H	1.3522508	-2.2384243	0.0103564
H	0.0747598	-1.7858409	-1.0901771
H	-0.2476325	-2.1765508	1.9186290
H	-1.5223369	-1.5360495	0.8691409
C	-1.0849399	-3.6177862	0.5677352
C	0.0566866	-4.6316108	0.7309237
C	-2.2896282	-4.0601053	1.4082657
H	-1.3935189	-3.6333205	-0.4897027
C	-0.4272499	-6.0555336	0.6374668
H	0.5560347	-4.4699739	1.7010900
H	0.8286088	-4.4589203	-0.0295505
C	-2.8297198	-5.4107625	0.9208981
H	-1.9787315	-4.1567867	2.4567716
H	-3.0799479	-3.2996528	1.3717937
C	-1.7177633	-6.4020675	0.6976550
H	0.3313627	-6.8251872	0.5014011
H	-3.5453155	-5.8175318	1.6468338
H	-3.3975436	-5.2759987	-0.0125885
H	-1.9982954	-7.4490183	0.5911217
H	1.3289408	-0.2749483	-0.0024112
H	1.8280148	0.5171679	-0.0513630

TS3a^{2+} : H_2 isotope exchange via **1**$^{2+}$**.**THF
107
Energy = -2977.229501668

Ca	-1.7533576	-0.0129145	-0.0544912
H	0.1105691	0.0093218	-1.2637408
N	-1.8480483	-2.5625570	-0.6921506
N	-2.5632893	-1.3676906	2.0013784
N	-4.2410638	0.7879089	0.7414283
N	-3.4246985	-0.3518488	-1.9732139
C	-2.5859500	-3.2764168	0.3778862
H	-2.4019288	-4.3602044	0.3123451
H	-3.6565970	-3.1332169	0.2140806
C	-2.1941067	-2.7887758	1.7671911
H	-2.6584485	-3.4383046	2.5259132
H	-1.1090196	-2.8543917	1.8907048
C	-4.0323265	-1.2501223	2.1773614
H	-4.3308135	-1.6578809	3.1559224
H	-4.5178179	-1.8680853	1.4190490
C	-4.5248216	0.1835946	2.0672178
H	-5.6042305	0.2084944	2.2865221
H	-4.0351506	0.8094201	2.8188960
C	-5.1690327	0.2466876	-0.2836057
H	-6.1547934	0.7288807	-0.1928820
H	-5.3245273	-0.8158362	-0.0851180
C	-4.6500302	0.4435829	-1.7023620
H	-5.4455903	0.1861815	-2.4190345
H	-4.3971810	1.4954029	-1.8644926
C	-3.7677047	-1.7917052	-2.0902835
H	-4.2830155	-1.9805213	-3.0449455
H	-4.4732135	-2.0441150	-1.2960141
C	-2.5448278	-2.6889685	-1.9958244
H	-2.8462871	-3.7321635	-2.1800936
H	-1.8225385	-2.4234169	-2.7726299
C	-0.5022147	-3.1495601	-0.8388582
H	0.0224077	-3.0989542	0.1165521
H	0.0522282	-2.5548533	-1.5655448
H	-0.5580534	-4.1976427	-1.1738977
C	-1.8803291	-0.8960540	3.2273393
H	-2.0883670	0.1635728	3.3908637
H	-0.8041203	-1.0284842	3.1001320
H	-2.2142525	-1.4605505	4.1111794
C	-4.4611170	2.2461728	0.8508976
H	-4.2243169	2.7329868	-0.0955536
H	-3.8091875	2.6510451	1.6264602
H	-5.5065855	2.4651925	1.1185989
C	-2.8102578	0.1068137	-3.2399746
H	-1.8482172	-0.3874791	-3.3896572
H	-2.6394617	1.1824994	-3.1893253
H	-3.4647050	-0.1060715	-4.0987053
Ca	1.8666454	-0.0591923	0.0173255
H	0.0751693	-0.1072639	1.2764078
N	3.1537101	1.1639839	1.8882690
N	3.3601235	1.6900084	-1.0709905
N	3.4120939	-1.2704813	-1.6298940
N	3.1999528	-1.8232137	1.3266338
C	4.2772830	1.8956688	1.2453455
H	4.7103988	2.6220045	1.9493883
H	5.0662629	1.1766586	1.0126935
C	3.8498026	2.6234590	-0.0233865
H	4.6912078	3.2258801	-0.3975507
H	3.0356552	3.3198865	0.1985991
C	4.5002314	0.9829758	-1.7093903
H	5.0389711	1.6652695	-2.3844874
H	5.2049462	0.6926400	-0.9264756
C	4.0546602	-0.2440279	-2.4925138
H	4.9200491	-0.6702746	-3.0219968
H	3.3237992	0.0403340	-3.2543495
C	4.4377986	-2.0081143	-0.8476219
H	4.9598302	-2.7336331	-1.4896022

H 5.1900160 -1.2911577 -0.5103429
C 3.8408407 -2.7411735 0.3471478
H 4.6265275 -3.3401631 0.8324152
H 3.0701923 -3.4411504 0.0113762
C 4.2358281 -1.0890806 2.1007566
H 4.7011178 -1.7592661 2.8395457
H 5.0272203 -0.7852516 1.4115541
C 3.6768574 0.1308839 2.8218425
H 4.4590785 0.5542040 3.4701566
H 2.8493197 -0.1632628 3.4731806
C 2.3206749 2.1179508 2.6562290
H 1.9137109 2.8781378 1.9871617
H 1.4840111 1.5839907 3.1099556
H 2.9114462 2.6142623 3.4408803
C 2.6066537 2.4450980 -2.0962533
H 2.1897954 1.7597699 -2.8364846
H 1.7809571 2.9693049 -1.6141529
H 3.2482536 3.1786496 -2.6065740
C 2.6702530 -2.2128421 -2.4982578
H 2.2633549 -3.0288020 -1.8985768
H 1.8453957 -1.6822131 -2.9805939
H 3.3274116 -2.6436645 -3.2680809
C 2.3720674 -2.6263343 2.2554966
H 1.8751808 -1.9738132 2.9753514
H 1.6046629 -3.1553970 1.6875633
H 2.9873426 -3.3576967 2.8010268
O -1.6795590 2.4540917 -0.4361659
C -1.3390581 3.3869711 0.6302620
C -0.1855440 4.2015146 0.0747429
C -0.6086178 4.3745884 -1.3911633
C -1.2728201 3.0334204 -1.7179243
H -1.0823668 2.7876065 1.5072495
H -2.2068735 4.0217196 0.8436956
H 0.7398540 3.6191999 0.1486180
H -0.0470547 5.1504594 0.5975028
H -1.3314748 5.1913007 -1.4759741
H 0.2256782 4.5924433 -2.0623774
H -2.1644233 3.1512953 -2.3403123
H -0.5882224 2.3161414 -2.1820777
H 0.1373612 -1.1094963 1.0811350
H 0.0429712 0.9273590 1.2201137

TS3^{2+} : H_2 isotope exchange via **1^{2+}**
94
Energy = -2744.626240437
Ca 0.1319398 0.0308974 1.7607364
Ca 0.1285622 0.0341257 -1.7608576
H 1.3341265 0.2748690 -0.0010021
H -0.8386600 -1.2086795 -0.0004439
N 0.2968007 -2.0956590 3.0670502
N 0.2911977 -2.0902143 -3.0710211
N -0.2666073 2.0990269 3.1230947
N -0.2721932 2.1046365 -3.1192440
N 2.1120326 0.2738424 3.2990689
N 2.1058860 0.2796523 -3.3020455
N -2.1141682 -0.2806964 2.9169521
N -2.1194938 -0.2754431 -2.9139199
C 0.9951794 2.4474695 3.8310641
C 0.9884408 2.4542339 -3.8286998
C -1.0697250 -2.4739103 3.5143833
C -1.0760735 -2.4678155 -3.5166792
C 1.7148046 1.2173168 4.3753584
C 1.7072505 1.2249965 -4.3761577
C -1.8718947 -1.2704320 3.9992823
C -1.8790923 -1.2635079 -3.9982145
C 0.8535630 -3.1574062 2.1984862
C 0.8497049 -3.1531544 -2.2050810
C -0.6657485 3.2201643 2.2415567
C -0.6695811 3.2243997 -2.2352056
C 1.1868529 -1.8577399 4.2331933
C 1.1792562 -1.8502591 -4.2382797
C -1.3440150 1.7972345 4.1013795
C -1.3511425 1.8042904 -4.0962558
C 2.4348678 -1.0644716 3.8603529
C 2.4278814 -1.0577416 -3.8659940
C -2.5110120 1.0372585 3.4802144
C -2.5172558 1.0434308 -3.4744651
C 3.2899848 0.7943917 2.5670547
C 3.2848795 0.7986659 -2.5706892
C -3.1941023 -0.7633245 2.0266602
C -3.1979553 -0.7592418 -2.0224996
H 0.7987169 3.1477941 4.6563241
H 0.7906368 3.1558969 -4.6525065
H -1.0247218 -3.2279253 4.3142151
H -1.0323267 -3.2203856 -4.3179473
H 1.6353388 2.9703295 3.1153297
H 1.6297414 2.9759619 -3.1131711
H -1.5713430 -2.9389295 2.6614765
H -1.5763125 -2.9344189 -2.6638058
H 0.1900294 -3.2957460 1.3415332
H 0.1874818 -3.2930489 -1.3473972
H 0.1252774 3.3955690 1.5072727
H 0.1229009 3.3984036 -1.5021266
H 0.6135170 -1.3189042 4.9920535
H 0.6047064 -1.3100047 -4.9952044
H -0.9058651 1.2135049 4.9150276
H -0.9142802 1.2217187 -4.9114256
H 1.0651413 0.6822331 5.0731120
H 1.0566033 0.6911680 -5.0739549
H -1.3361894 -0.7675861 4.8087394
H -1.3448182 -0.7593921 -4.8078295
H 0.9538009 -4.1095347 2.7399131
H 0.9499380 -4.1043070 -2.7482203
H -0.8311480 4.1431623 2.8166734
H -0.8360605 4.1483262 -2.8085250
H 1.4901546 -2.8146522 4.6843355
H 1.4816989 -2.8063919 -4.6916481
H -1.7234770 2.7283832 4.5490216
H -1.7312223 2.7360982 -4.5420069
H 2.5978262 1.5407223 4.9474708

H	2.5895034	1.5493118	-4.9489319
H	-2.8275243	-1.6183970	4.4200796
H	-2.8354654	-1.6107707	-4.4179067
H	1.8385478	-2.8600568	1.8299890
H	1.8349550	-2.8557677	-1.8373095
H	-1.5816932	2.9637861	1.7059765
H	-1.5845697	2.9670840	-1.6984836
H	3.0598088	1.7692434	2.1328017
H	3.0553551	1.7727555	-2.1344334
H	-2.9100704	-1.7166907	1.5783655
H	-2.9133763	-1.7133950	-1.5762224
H	3.0086997	-1.6027021	3.1007397
H	3.0028137	-1.5972620	-3.1081382
H	-2.9396545	1.6177996	2.6586014
H	-2.9448968	1.6226974	-2.6514071
H	3.0826503	-0.9733520	4.7452341
H	3.0743787	-0.9651737	-4.7516591
H	-3.3015431	0.9169332	4.2366843
H	-3.3088288	0.9241779	-4.2300207
H	3.5338109	0.1105969	1.7514863
H	3.5297286	0.1133889	-1.7566532
H	-3.3432084	-0.0339512	1.2279020
H	-3.3455407	-0.0310376	-1.2223500
H	4.1584712	0.8978674	3.2345540
H	4.1524939	0.9033169	-3.2391263
H	-4.1365548	-0.8928091	2.5794323
H	-4.1414102	-0.8876448	-2.5738408
H	-1.1846633	-0.2246479	0.0009176
H	-1.2550870	0.8029220	0.0020247

TS4a^{2+} : anti-Markovnikov styrene addition
108
Energy = -3053.282536039

Ca	0.1048798	0.2946339	1.9697449
Ca	-0.0247148	0.4816243	-1.6123797
H	0.1566965	1.5430289	0.2579624
N	1.7663884	-0.8863724	3.5696594
N	0.6383360	-0.7895184	-3.8485767
N	-1.6982950	1.5566313	3.1527038
N	-0.9340581	2.8504261	-2.1432285
N	1.2646104	2.0859907	3.3288765
N	1.8308246	1.7937424	-2.7634030
N	-1.2307902	-1.3969360	3.3808218
N	-2.1224033	0.2813967	-3.1285065
C	-1.1073663	2.8352445	3.6291506
C	0.2517417	3.7024692	-2.4102380
C	1.0641551	-2.0732607	4.1319882
C	-0.6325887	-1.1990801	-4.5042635
C	0.2614444	2.6461943	4.2729570
C	1.2626696	3.0423939	-3.3354404
C	-0.3775767	-1.7930071	4.5323566
C	-1.7040093	-0.1207287	-4.4976637
C	3.0518637	-1.3515188	2.9950858
C	1.4923502	-1.9971854	-3.7761551
C	-2.8088361	1.8497478	2.2202465
C	-1.6827348	3.4543953	-1.0217883
C	2.0450722	0.1109085	4.6396891
C	1.3444900	0.2423290	-4.6524738
C	-2.1881685	0.7332121	4.2862552
C	-1.8170675	2.7532527	-3.3290890
C	2.3985761	1.4812294	4.0756907
C	2.4181721	0.9612215	-3.8445275
C	-2.4557415	-0.7081765	3.8706078
C	-2.8047994	1.6021514	-3.1908694
C	1.7724476	3.1529182	2.4359146
C	2.8751634	2.1371792	-1.7715547
C	-1.6381166	-2.6038585	2.6239423
C	-3.0719647	-0.7195996	-2.5905471
H	-1.7782150	3.3331013	4.3452725
H	-0.0518315	4.6700867	-2.8402742
H	1.6087462	-2.4617391	5.0060962
H	-0.4464921	-1.5023996	-5.5468681
H	-1.0223116	3.4915601	2.7582923
H	0.7124843	3.9088773	-1.4398523
H	1.0964866	-2.8551196	3.3661126
H	-0.9875744	-2.0876969	-3.9724067
H	2.8559700	-2.0535588	2.1818671
H	0.9728683	-2.7724359	-3.2143495
H	-2.4244592	2.4552034	1.3997693
H	-1.0294146	3.4807649	-0.1483194
H	1.1596344	0.1909292	5.2747487
H	0.6103662	0.9657810	-5.0142145
H	-1.4366899	0.7615310	5.0795764
H	-1.1945088	2.6069042	-4.2152740
H	0.1836621	1.9701057	5.1278760
H	0.7949567	2.8005484	-4.2924043
H	-0.4137097	-0.9927165	5.2748503
H	-1.3460335	0.7700535	-5.0185202
H	3.6651543	-1.8476426	3.7614265
H	1.7192399	-2.3716840	-4.7861983
H	-3.6229888	2.3917759	2.7232934
H	-2.0231855	4.4704829	-1.2744489
H	2.8650637	-0.2433463	5.2819273
H	1.8007475	-0.2178658	-5.5421445
H	-3.1096272	1.1656915	4.7047928
H	-2.3706679	3.6928799	-3.4812840
H	0.6083781	3.6135920	4.6660723
H	2.0701415	3.7581766	-3.5526514
H	-0.7905142	-2.6897857	5.0180428
H	-2.5745411	-0.4847212	-5.0644038
H	3.6130726	-0.5053446	2.5944040
H	2.4337272	-1.7720664	-3.2718509
H	-3.2096399	0.9214040	1.8064405
H	-2.5569825	2.8450831	-0.7841636
H	0.9471442	3.5817498	1.8655197
H	2.4279543	2.7062098	-0.9543474
H	-0.7566252	-3.1613820	2.3010362
H	-2.6161274	-1.7103468	-2.6178182
H	3.2395141	1.3988365	3.3817501
H	3.0858577	0.2344034	-3.3732564

H	-3.1898481	-0.7371479	3.0613292
H	-3.3873588	1.7131435	-2.2723083
H	2.7239980	2.1394142	4.8949136
H	3.0351584	1.5750342	-4.5181191
H	-2.8940532	-1.2551927	4.7185252
H	-3.5198464	1.6255806	-4.0267909
H	2.4833216	2.7193066	1.7286237
H	3.2967946	1.2186396	-1.3604836
H	-2.1977371	-2.2981457	1.7379953
H	-3.3152862	-0.4712749	-1.5535812
H	2.2710424	3.9476328	3.0105450
H	3.6789598	2.7316685	-2.2316522
H	-2.2619156	-3.2668051	3.2411282
H	-4.0002412	-0.7447819	-3.1803217
C	1.9147669	-0.9501626	-0.2427078
C	0.8080771	-1.6894101	0.1661553
H	2.5151861	-0.3676923	0.4468882
H	2.2933041	-1.0390026	-1.2567335
H	0.7079641	-1.8489655	1.2497536
H	-0.6189988	-0.8716060	0.1360855
C	0.3786189	-2.9158557	-0.5901431
C	1.3601501	-3.8624775	-0.9219444
C	-0.9502378	-3.1819670	-0.9344219
C	1.0199662	-5.0341642	-1.5971611
H	2.3933266	-3.6633909	-0.6529923
C	-1.2928621	-4.3475872	-1.6207444
H	-1.7088941	-2.4543067	-0.6621338
C	-0.3072867	-5.2770381	-1.9579165
H	1.7936143	-5.7532297	-1.8505369
H	-2.3289263	-4.5299580	-1.8925240
H	-0.5710794	-6.1825685	-2.4962825

TS4^{2+} : Markovnikov styrene addition to **1**$^{2+}$
108
Energy = -3053.291651741

Ca	-0.3792556	-0.3735268	1.8429682
Ca	-0.2452146	0.0205262	-1.7993470
H	-0.8897859	0.8813899	0.1728055
N	1.8460155	-0.6826644	3.2360985
N	-0.7626159	-1.8704826	-3.4864252
N	-2.5016653	-0.2867467	3.2777148
N	-1.1285178	2.3000585	-2.6017308
N	-0.1651471	1.6145366	3.3843288
N	0.8627320	0.6085200	-4.1016921
N	-0.5340484	-2.5889713	3.0464570
N	-2.7409570	-0.2121628	-2.0380773
C	-2.5403010	1.0472855	3.9395390
C	-0.3442190	2.7662072	-3.7731780
C	1.8557721	-2.1089892	3.6686544
C	-2.2103567	-2.2009737	-3.4171885
C	-1.1844923	1.5003884	4.4605109
C	-0.0230748	1.6246314	-4.7265321
C	0.4974933	-2.6325428	4.1172828
C	-3.0724098	-0.9519496	-3.2767336
C	3.1856595	-0.3833224	2.6670262
C	0.0264851	-3.0741255	-3.1425094
C	-3.7904867	-0.4743671	2.5708216
C	-1.0405302	3.3105477	-1.5231239
C	1.6319191	0.2197813	4.4021506
C	-0.4195100	-1.4159058	-4.8583297
C	-2.3366945	-1.3717822	4.2829399
C	-2.5497981	2.0995644	-2.9892678
C	1.1995983	1.6164958	3.9745382
C	0.8846960	-0.6349893	-4.9155750
C	-1.8937990	-2.6828122	3.6448466
C	-3.2898980	1.1676081	-2.0401742
C	-0.3807793	2.8670949	2.6235106
C	2.2418334	1.1442897	-4.0511850
C	-0.3372682	-3.7230118	2.1125723
C	-3.2714308	-0.9216373	-0.8590060
H	-3.2603813	1.0424980	4.7716313
H	-0.8836291	3.5568678	-4.3165358
H	2.5741756	-2.2528406	4.4902190
H	-2.5240342	-2.7685901	-4.3060007
H	-2.9137765	1.7583349	3.1973596
H	0.5785849	3.2133444	-3.3939386
H	2.2246156	-2.6939908	2.8203147
H	-2.3547303	-2.8556190	-2.5540720
H	3.3771846	-1.0262746	1.8064557
H	-0.2086656	-3.3720158	-2.1178962
H	-3.9178263	0.3249653	1.8382347
H	0.0059893	3.4279571	-1.2295367
H	0.8698465	-0.2213092	5.0487615
H	-1.2442826	-0.8015764	-5.2270691
H	-1.5986740	-1.0475019	5.0205870
H	-2.5747615	1.6980457	-4.0052844
H	-0.8145377	0.7971858	5.2099271
H	-0.9483465	1.1336943	-5.0375909
H	0.1312343	-2.0512458	4.9664395
H	-2.9109464	-0.2871285	-4.1296581
H	3.9674719	-0.5573946	3.4204977
H	-0.1998351	-3.9092477	-3.8221484
H	-4.6322808	-0.4439775	3.2778655
H	-1.4314986	4.2831947	-1.8591240
H	2.5526425	0.2857535	4.9998188
H	-0.3452705	-2.2802987	-5.5358782
H	-3.2793460	-1.5310795	4.8270795
H	-3.0727873	3.0676181	-3.0212878
H	-1.3060687	2.4676650	4.9706390
H	0.4461538	2.0268719	-5.6377421
H	0.6227757	-3.6647326	4.4769297
H	-4.1359855	-1.2370484	-3.2909018
H	3.2316044	0.6548327	2.3381103
H	1.0934864	-2.8521816	-3.2095936
H	-3.8048688	-1.4363164	2.0548941
H	-1.5984170	2.9625427	-0.6532360
H	-1.3792091	2.8664504	2.1835002
H	2.2977648	1.9977014	-3.3732974
H	0.6771637	-3.7001527	1.7104654
H	-2.8724646	-1.9360144	-0.8121245

H	1.8849559	2.0081932	3.2171389
H	1.7063229	-1.2493991	-4.5357486
H	-2.5831764	-2.9650257	2.8440637
H	-3.1954760	1.5378031	-1.0149331
H	1.2486598	2.2975036	4.8369528
H	1.1154103	-0.4051492	-5.9679038
H	-1.9300508	-3.4855527	4.3959126
H	-4.3607123	1.1616656	-2.2972472
H	0.3437186	2.9233450	1.8094823
H	2.9126050	0.3700795	-3.6805691
H	-1.0427801	-3.6349927	1.2829763
H	-2.9376649	-0.3762044	0.0240633
H	-0.2709161	3.7483816	3.2724005
H	2.5768871	1.4588505	-5.0513301
H	-0.4903034	-4.6855821	2.6217059
H	-4.3717579	-0.9664511	-0.8754521
C	2.3289694	-0.9275610	-1.1373030
C	1.5781680	-1.6570617	-0.2318494
H	2.6306778	-1.4219890	-2.0591156
H	1.6435858	-1.3935833	0.8223085
H	-0.1847078	-1.4691977	-0.1515199
C	2.9198019	0.3816775	-0.8664350
C	4.2019823	0.6960680	-1.3688470
C	2.2595523	1.3813520	-0.1211467
C	4.7941488	1.9292824	-1.1215213
H	4.7382387	-0.0524935	-1.9469212
C	2.8535752	2.6199687	0.1252593
H	1.2352178	1.2120151	0.2278749
C	4.1262954	2.9028610	-0.3693260
H	5.7859285	2.1350518	-1.5147001
H	2.3142894	3.3758958	0.6899363
H	4.5869061	3.8681441	-0.1837787
H	1.4043266	-2.7101756	-0.4108581

TS5a^{2+} : hydrogenolysis with H_2
110
Energy = -3054.488281808

Ca	0.1829530	0.7456962	1.6885057
Ca	0.1395704	0.1821466	-1.8859054
H	-0.9137660	0.9309762	-0.1629397
N	2.3300389	1.0239515	3.0104880
N	1.3761947	-1.6419580	-3.2293672
N	-1.7160260	2.1175575	2.6375855
N	-1.4389553	1.5845462	-3.2992439
N	0.8751525	3.1762870	1.4955644
N	1.5315235	1.3614752	-3.6878859
N	-0.2906613	0.0142911	4.2503923
N	-1.6028644	-1.3975340	-2.8252364
C	-1.4905563	3.5189609	2.1974238
C	-0.6381332	2.5312182	-4.1187364
C	2.1855635	0.3598946	4.3339336
C	0.4064771	-2.7422120	-3.4809520
C	-0.0384810	3.9521383	2.3719966
C	0.5971739	1.8785191	-4.7243445
C	0.8383860	0.6465721	4.9844443
C	-0.9767926	-2.2389944	-3.8780304
C	3.4610285	0.4049340	2.2799118
C	2.5814128	-2.2053821	-2.5760945
C	-2.9833944	1.6172284	2.0562432
C	-2.3909072	2.3563781	-2.4682857
C	2.5804063	2.4788125	3.1940543
C	1.7735830	-0.9914773	-4.5055035
C	-1.7694241	2.0489384	4.1181129
C	-2.1834926	0.6371282	-4.1688639
C	2.2895096	3.2864372	1.9369183
C	2.4504652	0.3545193	-4.2847560
C	-1.5832121	0.6353489	4.6479901
C	-2.6995780	-0.5715172	-3.3985902
C	0.7757761	3.6799958	0.1104037
C	2.3398327	2.4802366	-3.1502635
C	-0.3589655	-1.4161162	4.6351237
C	-2.1648289	-2.2566349	-1.7580216
H	-2.1478804	4.2095984	2.7467151
H	-1.2491921	2.9666972	-4.9239003
H	2.9930251	0.6743495	5.0119196
H	0.7798355	-3.4201084	-4.2635642
H	-1.7738669	3.5736222	1.1427791
H	-0.3437663	3.3555414	-3.4622660
H	2.3052728	-0.7155704	4.1738692
H	0.3408806	-3.3257535	-2.5565922
H	3.2477818	-0.6549116	2.1187381
H	2.2852796	-2.7424316	-1.6739474
H	-2.8924901	1.6235839	0.9680226
H	-1.8310227	3.0311607	-1.8182197
H	1.9550773	2.8314680	4.0174479
H	0.8787831	-0.8610125	-5.1186503
H	-0.9943918	2.7069908	4.5180417
H	-1.5157272	0.3106101	-4.9704962
H	0.2740384	3.8087821	3.4094944
H	0.3039180	1.0446213	-5.3660488
H	0.6656825	1.7246139	5.0212153
H	-0.9145184	-1.6471652	-4.7944285
H	4.3992680	0.4978185	2.8462228
H	3.1083865	-2.8966357	-3.2499081
H	-3.8381192	2.2341451	2.3710696
H	-3.0774249	2.9433807	-3.0967644
H	3.6248639	2.6481253	3.4961928
H	2.4517958	-1.6486895	-5.0704050
H	-2.7316417	2.4386760	4.4842476
H	-3.0316168	1.1471424	-4.6505587
H	0.0486354	5.0286698	2.1600919
H	1.1116652	2.6063612	-5.3693083
H	0.8559246	0.2952877	6.0271829
H	-1.6180659	-3.1034975	-4.1059762
H	3.5809855	0.8864129	1.3075982
H	3.2652207	-1.4028357	-2.2906043
H	-3.1609679	0.5887680	2.3815532
H	-2.9679263	1.6813845	-1.8352266
H	-0.2471556	3.5690983	-0.2515794
H	1.6817737	3.2643082	-2.7707727

H	0.5814657	-1.9194776	4.4001645
H	-1.3965619	-2.9250332	-1.3673968
H	2.9054579	2.9222143	1.1091084
H	3.2992000	0.2443047	-3.6046209
H	-2.3776008	-0.0153944	4.2707544
H	-3.3261477	-0.2450904	-2.5643626
H	2.5667338	4.3375031	2.1111324
H	2.8519737	0.7203145	-5.2415402
H	-1.6801445	0.6522190	5.7448409
H	-3.3357854	-1.1791981	-4.0589270
H	1.4324038	3.0785587	-0.5202352
H	2.9652029	2.1147311	-2.3316767
H	-1.1675065	-1.9055195	4.0880278
H	-2.5234387	-1.6243752	-0.9414956
H	1.0713232	4.7384926	0.0488098
H	2.9822230	2.9155222	-3.9296684
H	-0.5470628	-1.5193344	5.7141203
H	-2.9997252	-2.8649615	-2.1353385
C	0.7966051	-1.5063086	0.2620252
C	-0.2400529	-1.9515332	1.2958230
H	1.7684452	-1.9640841	0.4860509
H	0.5026827	-1.9288452	-0.7134568
H	0.1801503	-1.8008020	2.3011445
H	-1.1469231	-1.3178734	1.2408833
C	-0.7292784	-3.3882509	1.2147976
C	0.1149556	-4.4202059	0.7898441
C	-2.0529989	-3.6993313	1.5484337
C	-0.3528217	-5.7322823	0.7018454
H	1.1430185	-4.1940171	0.5196013
C	-2.5270823	-5.0082409	1.4576633
H	-2.7253081	-2.9041811	1.8661823
C	-1.6763085	-6.0306193	1.0326859
H	0.3160448	-6.5220052	0.3707700
H	-3.5605193	-5.2281452	1.7109000
H	-2.0426613	-7.0501889	0.9555096
H	1.4164816	-0.0460175	-0.0393705
H	1.7335572	0.8040849	-0.2875636

TS5^{2+} : hydrogenolysis with H_2

110

Energy = -3054.495037638

Ca	-0.0913764	0.2610317	1.7436632
Ca	-0.1296203	0.3063824	-1.9300303
H	-0.6138351	1.3148453	-0.1051902
N	1.5888043	-0.7510320	3.3892879
N	-0.4239587	-1.6399241	-3.6967740
N	-1.0920174	2.3892681	2.6409424
N	0.0971975	2.6061312	-3.0247782
N	1.8452030	1.8909752	2.0267286
N	1.9571409	0.2764704	-3.3841355
N	-1.3493070	-0.2288274	4.0940391
N	-2.2591734	0.7324363	-3.2590363
C	-0.0768424	3.4459143	2.3920099
C	1.4761817	2.7279185	-3.5675265
C	0.8503034	-1.3360836	4.5389218
C	-1.8183678	-1.5467325	-4.2103422
C	1.3251214	3.0304668	2.8258722
C	1.9408785	1.4652965	-4.2807842
C	-0.2339325	-0.4067466	5.0641630
C	-2.2807540	-0.1222881	-4.4753971
C	2.3830392	-1.8218344	2.7423699
C	-0.1908251	-3.0554485	-3.3190092
C	-2.3225637	2.7197069	1.8847101
C	-0.1518007	3.7344838	-2.0988974
C	2.4949988	0.3280640	3.8597284
C	0.5543404	-1.2746522	-4.7583080
C	-1.4010326	2.2845882	4.0872195
C	-0.9072041	2.6471450	-4.1190779
C	2.9713739	1.2154989	2.7210106
C	1.9358456	-0.9732440	-4.1926951
C	-2.1292158	0.9933435	4.4311512
C	-2.2753978	2.1672730	-3.6534828
C	2.3248561	2.3807162	0.7192199
C	3.1924692	0.2938413	-2.5650668
C	-2.2613996	-1.3892851	4.2290216
C	-3.4513555	0.4483287	-2.4281282
H	-0.3545773	4.3796129	2.9047825
H	1.5512101	3.5803173	-4.2597431
H	1.5443467	-1.5853458	5.3561835
H	-1.9237442	-2.1304273	-5.1378298
H	-0.0910709	3.6450341	1.3167297
H	2.1353526	2.9457273	-2.7218799
H	0.4089460	-2.2758285	4.1929017
H	-2.4625783	-2.0207086	-3.4630652
H	1.7147304	-2.6263849	2.4333019
H	-0.8705867	-3.3426974	-2.5142982
H	-2.0737974	2.7473059	0.8221989
H	0.6113902	3.7344211	-1.3204347
H	1.9623539	0.9295676	4.6001400
H	0.1760939	-0.4004010	-5.2934310
H	-0.4635448	2.3471071	4.6445367
H	-0.5511816	2.0187665	-4.9393617
H	1.3241308	2.7385750	3.8788232
H	1.2837866	1.2477680	-5.1254418
H	0.1895913	0.5748784	5.2873372
H	-1.6465872	0.3465788	-5.2309299
H	3.1376223	-2.2239045	3.4353392
H	-0.3593386	-3.7179834	-4.1804759
H	-2.7385473	3.6856334	2.2082101
H	-0.1157036	4.6961259	-2.6322641
H	3.3673403	-0.1060547	4.3717680
H	0.6306681	-2.0867017	-5.4964686
H	-2.0176562	3.1373825	4.4106103
H	-0.9953377	3.6694838	-4.5167937
H	2.0001671	3.8953456	2.7388828
H	2.9435861	1.6366627	-4.6988779
H	-0.6238927	-0.8021140	6.0139488
H	-3.2968692	-0.1541445	-4.8958752
H	2.8848244	-1.4318431	1.8548559
H	0.8347613	-3.1891119	-2.9742844

H	-3.0770767	1.9453044	2.0427962
H	-1.1256150	3.6227565	-1.6211759
H	1.4991281	2.8535462	0.1881783
H	3.2620007	1.2389254	-2.0231746
H	-1.7181798	-2.3137651	4.0296313
H	-3.4756663	-0.6099122	-2.1593085
H	3.4946062	0.6150541	1.9717082
H	2.2652406	-1.7882690	-3.5419354
H	-3.0726110	0.9337675	3.8812110
H	-2.6017283	2.7422102	-2.7832037
H	3.6926144	1.9514759	3.1073223
H	2.6623117	-0.9091694	-5.0160158
H	-2.3844201	1.0014097	5.5021867
H	-3.0152400	2.3395954	-4.4487477
H	2.6666188	1.5257809	0.1325498
H	3.1601287	-0.5232312	-1.8400541
H	-3.0859255	-1.2987873	3.5185137
H	-3.4017740	1.0467640	-1.5144038
H	3.1469806	3.1027272	0.8379125
H	4.0866975	0.1883933	-3.1963045
H	-2.6780447	-1.4399064	5.2460067
H	-4.3789909	0.6924719	-2.9655530
C	-0.9168524	-1.7447720	-0.0304528
C	-2.2534503	-1.3202962	0.5770123
H	-1.0438203	-1.8778877	-1.1160653
H	-2.1803362	-1.0980084	1.6536925
H	-2.6390619	-0.4172101	0.0977229
H	0.3605309	-0.9238906	-0.0666606
H	1.2347385	-0.4437697	-0.1205850
H	-3.0272105	-2.0985645	0.5071639
C	-0.3010758	-2.9703579	0.5391496
C	-0.8243698	-3.6332225	1.6658685
C	0.9072967	-3.4671260	0.0041868
C	-0.1730740	-4.7322554	2.2305524
H	-1.7683300	-3.3072448	2.0897090
C	1.5495013	-4.5714902	0.5556733
H	1.3542191	-2.9651911	-0.8489068
C	1.0167209	-5.2103765	1.6799330
H	-0.6075614	-5.2245039	3.0967505
H	2.4768174	-4.9294668	0.1165307
H	1.5204083	-6.0675622	2.1162032

TS6a^{2+} : second ethylene addition to dimer **1**$^{2+}$
104
Energy = -2900.738747157

Ca	0.0923098	-0.2089386	1.8459294
Ca	-0.2234610	-0.2952330	-1.7320950
H	-1.0081685	0.7029624	0.1178326
N	1.6395882	-1.4155261	3.5980499
N	0.3467972	-1.9339165	-3.6655526
N	-1.0573907	1.9441078	2.8738891
N	-0.9704358	2.0268112	-2.6823983
N	1.8891659	1.4014088	2.5002164
N	1.7252679	0.7237317	-3.1296529
N	-1.3389673	-0.9043736	3.8824030
N	-2.3887351	-0.6162790	-3.2589623
C	-0.0012690	2.9899249	2.9048429
C	0.2337998	2.7213197	-3.2094432
C	0.7597281	-2.0822374	4.5941047
C	-0.9230244	-2.3346768	-4.3310679
C	1.3515287	2.4752108	3.3734399
C	1.1421773	1.7854941	-3.9913008
C	-0.4265735	-1.2301212	5.0126500
C	-1.8677385	-1.1605275	-4.5417689
C	2.5874146	-2.4365887	3.0908256
C	1.0384254	-3.1640421	-3.2099665
C	-2.2218182	2.5201880	2.1614708
C	-1.5931738	2.8943117	-1.6580345
C	2.4164340	-0.3164504	4.2315692
C	1.2197706	-1.2205687	-4.6376533
C	-1.4746818	1.5691107	4.2493372
C	-1.9419664	1.8077149	-3.7860854
C	2.9716976	0.6592005	3.2014458
C	2.2890718	-0.3682895	-3.9691313
C	-2.2123609	0.2372379	4.2713831
C	-2.9726069	0.7362348	-3.4693695
C	2.4145947	1.9824390	1.2458346
C	2.8271424	1.3176796	-2.3376030
C	-2.1967672	-2.0798881	3.5963820
C	-3.4902158	-1.4963399	-2.7967215
H	-0.3063708	3.8268369	3.5521157
H	-0.0576230	3.5677332	-3.8495558
H	1.3339576	-2.3578903	5.4923348
H	-0.7131970	-2.8106153	-5.3009032
H	0.0785313	3.3884450	1.8889016
H	0.7689341	3.1410456	-2.3533099
H	0.4143277	-3.0159067	4.1400883
H	-1.3952039	-3.0928953	-3.6999401
H	2.0339039	-3.2229046	2.5733257
H	0.4138549	-3.6835997	-2.4797578
H	-1.9626445	2.6601484	1.1113804
H	-0.8890581	3.0346378	-0.8355228
H	1.7633751	0.2135780	4.9285118
H	0.5880152	-0.5962003	-5.2724085
H	-0.5846797	1.5158464	4.8804628
H	-1.3849850	1.5397255	-4.6859759
H	1.2737947	2.0805635	4.3890083
H	0.5800996	1.3088994	-4.7971702
H	-0.0775898	-0.2932190	5.4519978
H	-1.3499899	-0.3606999	-5.0751994
H	3.1546846	-2.8832723	3.9208643
H	1.2340751	-3.8381297	-4.0569949
H	-2.5109109	3.4854774	2.6024530
H	-1.8611403	3.8745499	-2.0806045
H	3.2443325	-0.7294964	4.8269554
H	1.7099810	-1.9462847	-5.3035834
H	-2.1193840	2.3493462	4.6812036
H	-2.4683763	2.7467653	-4.0145387
H	2.0578469	3.3180007	3.4156454
H	1.9446727	2.3686554	-4.4677537

H	-0.9841647	-1.7574651	5.8011082
H	-2.7034142	-1.4765681	-5.1842654
H	3.2875378	-1.9841038	2.3893947
H	1.9885617	-2.9065681	-2.7389525
H	-3.0773597	1.8462443	2.2253334
H	-2.4970413	2.4233954	-1.2670908
H	1.6158522	2.5150450	0.7229323
H	2.4466905	2.1372693	-1.7262082
H	-1.5768848	-2.9352670	3.3178871
H	-3.1358717	-2.5235318	-2.6977433
H	3.5450551	0.1198439	2.4421650
H	2.9148451	-0.9868795	-3.3197967
H	-3.0525745	0.2590490	3.5706839
H	-3.5181519	0.9946325	-2.5562410
H	3.6640638	1.3589456	3.6919024
H	2.9478839	0.0479968	-4.7469354
H	-2.6367749	0.0695987	5.2726534
H	-3.7132521	0.7081399	-4.2839986
H	2.7732147	1.1750247	0.6051627
H	3.2573181	0.5600049	-1.6832827
H	-2.8678105	-1.8493929	2.7658077
H	-3.8514915	-1.1586478	-1.8241034
H	3.2380545	2.6853486	1.4394956
H	3.6148202	1.7117861	-2.9968131
H	-2.7953445	-2.3540015	4.4773327
H	-4.3218745	-1.4811636	-3.5168234
C	1.2579853	-1.5986690	-0.0147729
C	2.7838658	-1.6464312	-0.2394833
H	0.8167717	-2.3206049	-0.7285935
H	1.0653367	-2.1570796	0.9261547
H	3.0389671	-1.4110896	-1.2791071
H	3.3275805	-0.9152186	0.3742197
H	3.2352703	-2.6287284	-0.0321418
C	-2.1668478	-0.2653302	0.2663234
C	-1.9000942	-1.6285354	0.1025189
H	-2.7812514	0.2452080	-0.4760871
H	-2.4204625	0.0909540	1.2670315
H	-1.5435502	-2.2526617	0.9160451
H	-1.9632608	-2.1222728	-0.8612392

TS6^{2+} : first ethylene addition to dimer **1**$^{2+}$
98
Energy = -2822.085589660

Ca	0.1012797	-0.0451337	1.7639793
Ca	0.0048553	-0.0118235	-1.7661852
H	-0.7479074	0.9028020	0.0301585
N	2.0911426	-0.3823236	3.4838470
N	1.8994564	-0.3169587	-3.5967413
N	-2.1129155	0.4007358	2.8979291
N	-2.2673400	0.4476240	-2.7721140
N	0.3700852	2.0932515	3.0827872
N	0.1994987	2.1468602	-3.0653032
N	-0.4409810	-2.0655285	3.2205216
N	-0.6114857	-2.0111197	-3.2225669
C	-2.1063922	1.8167840	3.3484819
C	-2.2877787	1.8705099	-3.2000284
C	1.8954765	-1.7313296	4.0827274
C	1.6749831	-1.6580726	-4.2034191
C	-0.7916235	2.2242550	4.0011428
C	-1.0108342	2.2909700	-3.9163200
C	0.4458015	-2.0580104	4.4123882
C	0.2105332	-1.9844525	-4.4598811
C	3.5025225	-0.3018080	3.0355275
C	3.3326131	-0.2388697	-3.2234147
C	-3.2373621	0.2070083	1.9574866
C	-3.3394061	0.2354145	-1.7760954
C	1.8585600	0.6818729	4.4984631
C	1.6100314	0.7608629	-4.5817632
C	-2.2399010	-0.5349154	4.0414861
C	-2.4530420	-0.4702759	-3.9221365
C	1.6357313	2.0471361	3.8588356
C	1.4203723	2.1161058	-3.9108536
C	-1.8615560	-1.9595453	3.6505174
C	-2.0529796	-1.9002596	-3.5747027
C	0.4003069	3.2286577	2.1323890
C	0.2809621	3.2657146	-2.0984933
C	-0.2526617	-3.3217453	2.4583725
C	-0.3826839	-3.2792302	-2.4916274
H	-2.9317147	2.0091566	4.0510887
H	-3.1499963	2.0713863	-3.8542367
H	2.4950385	-1.8318901	5.0010298
H	2.2250664	-1.7441715	-5.1535889
H	-2.2877417	2.4309059	2.4621166
H	-2.4223780	2.4705965	-2.2958881
H	2.2919815	-2.4574625	3.3662375
H	2.1106482	-2.3931540	-3.5193363
H	3.6819260	-1.0306885	2.2431487
H	3.5549834	-0.9773907	-2.4512469
H	-3.1210925	0.9127451	1.1354288
H	-3.1816117	0.9289230	-0.9504828
H	0.9846669	0.4076858	5.0940639
H	0.7051690	0.4931341	-5.1323503
H	-1.5909533	-0.1824453	4.8474926
H	-1.8484004	-0.1034955	-4.7557304
H	-0.6020241	1.6042138	4.8808214
H	-0.8687096	1.6856704	-4.8149400
H	0.0489597	-1.3313710	5.1250589
H	-0.2254061	-1.2494425	-5.1404694
H	4.1857204	-0.5078567	3.8729004
H	3.9705684	-0.4322095	-4.0986037
H	-4.2058938	0.3696360	2.4541948
H	-4.3335796	0.4020115	-2.2177575
H	2.7100929	0.7351065	5.1933240
H	2.4220559	0.8261899	-5.3214924
H	-3.2683340	-0.5277843	4.4341851
H	-3.5008473	-0.4592362	-4.2597513
H	-0.8768217	3.2616724	4.3589648
H	-1.1162827	3.3339278	-4.2519064
H	0.4128909	-3.0375808	4.9133582
H	0.1531217	-2.9571418	-4.9718816

H	3.7202975	0.6941497	2.6475897
H	3.5691581	0.7524974	-2.8345732
H	-3.2187568	-0.8052763	1.5472028
H	-3.2958070	-0.7828473	-1.3828594
H	-0.5111010	3.2307598	1.5318171
H	-0.5950170	3.2544332	-1.4474583
H	0.7924425	-3.4215201	2.1574501
H	0.6768198	-3.3821950	-2.2475136
H	2.4522838	2.2786135	3.1688736
H	2.2734479	2.3398407	-3.2639361
H	-2.4811613	-2.2973477	2.8156415
H	-2.6271869	-2.2526671	-2.7138371
H	1.6455418	2.8234049	4.6386703
H	1.3851960	2.9037196	-4.6783675
H	-2.0635343	-2.6369180	4.4936022
H	-2.2989715	-2.5642236	-4.4168009
H	1.2512424	3.1070660	1.4574763
H	1.1688835	3.1348899	-1.4748832
H	-0.8679647	-3.2909617	1.5563989
H	-0.9499471	-3.2638373	-1.5583418
H	0.4918778	4.1894365	2.6607301
H	0.3407324	4.2358927	-2.6140561
H	-0.5295481	-4.1986866	3.0622787
H	-0.6903545	-4.1466494	-3.0944458
C	2.2257746	0.2961019	-0.0570348
C	1.9185596	-1.0584150	-0.0629142
H	2.4353014	0.8387234	0.8591097
H	2.3854262	0.8575730	-0.9717234
H	2.0989459	-1.6673020	0.8214750
H	0.2917011	-1.4235982	-0.0213105
H	2.0493416	-1.6470350	-0.9694903

TS7^{2+} : hydrogenolysis with H_2
100
Energy = -2823.290577514

Ca	-0.0700134	-0.2927173	1.8061569
Ca	0.0675927	-0.1291389	-1.7829178
H	-1.0134890	0.3900237	0.0026057
N	1.9490129	-0.8054148	3.2581869
N	0.9302736	-1.8101236	-3.5449749
N	-1.6654149	1.3398767	2.9352483
N	-1.1773409	1.8501328	-2.8078297
N	1.1983865	1.8962524	2.1482300
N	1.6882727	1.1231194	-3.3340920
N	-0.9418007	-1.3166240	4.1401779
N	-1.9396871	-1.0837881	-2.9821031
C	-1.0667708	2.6933233	2.8058620
C	-0.1948344	2.7694677	-3.4383923
C	1.5236239	-1.6405842	4.4140040
C	-0.2373290	-2.6122251	-4.0002791
C	0.4228645	2.6943791	3.1312060
C	0.8913080	2.0285043	-4.2054513
C	0.2271059	-1.1480631	5.0443986
C	-1.4905627	-1.7722130	-4.2200133
C	2.9670138	-1.5442548	2.4748609
C	1.9863225	-2.7355302	-3.0705223
C	-2.9607935	1.3062155	2.2176244
C	-1.9670567	2.6089448	-1.8122463
C	2.5194674	0.4808013	3.7412940
C	1.4685783	-0.9873880	-4.6587910
C	-1.8694496	1.0113958	4.3669952
C	-2.0880038	1.2713427	-3.8294928
C	2.5501985	1.5539852	2.6619077
C	2.3977592	0.1141476	-4.1664735
C	-2.0803044	-0.4757525	4.5996805
C	-2.8415577	0.0528438	-3.3105384
C	1.3563231	2.6775250	0.9043552
C	2.6926475	1.9136577	-2.5859556
C	-1.3958800	-2.7267578	4.2089652
C	-2.6698815	-2.0289843	-2.1059876
H	-1.5859877	3.4120425	3.4577311
H	-0.6975016	3.4762035	-4.1163621
H	2.3136304	-1.6690073	5.1794221
H	-0.0003568	-3.1523443	-4.9297373
H	-1.2267356	3.0203319	1.7748583
H	0.2509004	3.3634442	-2.6349169
H	1.3990836	-2.6640969	4.0478744
H	-0.4233208	-3.3678033	-3.2308616
H	2.5325109	-2.4778480	2.1098852
H	1.5794237	-3.3746755	-2.2861477
H	-2.7751016	1.4854612	1.1569832
H	-1.2926170	3.0174330	-1.0574205
H	1.9220646	0.8216776	4.5901584
H	0.6274557	-0.5501272	-5.2023552
H	-0.9978958	1.3605856	4.9256384
H	-1.4925809	0.9968927	-4.7042748
H	0.5894796	2.2746878	4.1263653
H	0.4442420	1.4286935	-5.0013273
H	0.3166460	-0.0893632	5.2989774
H	-1.3060393	-1.0123908	-4.9834643
H	3.8491855	-1.7770258	3.0891793
H	2.3566916	-3.3645470	-3.8933680
H	-3.6562853	2.0596950	2.6165283
H	-2.5175085	3.4331359	-2.2908374
H	3.5410502	0.3228733	4.1185742
H	2.0105038	-1.6248896	-5.3738224
H	-2.7374919	1.5594172	4.7648292
H	-2.8137466	2.0278109	-4.1651055
H	0.7907650	3.7312907	3.1582881
H	1.5510313	2.7602394	-4.6953242
H	0.0563529	-1.6851959	5.9897906
H	-2.2901540	-2.4205075	-4.6086564
H	3.2785130	-0.9460318	1.6162341
H	2.8246877	-2.1701998	-2.6572654
H	-3.4184586	0.3196464	2.3234792
H	-2.6697920	1.9443153	-1.3095828
H	0.3767593	2.8955769	0.4755401
H	2.1960411	2.7037582	-2.0196003
H	-0.5724009	-3.4043091	3.9780762
H	-2.0532284	-2.9074095	-1.9068267

H	3.1320811	1.2039560	1.8038754
H	3.1973280	-0.3109913	-3.5535630
H	-2.9676522	-0.8199565	4.0601922
H	-3.3821854	0.3082144	-2.3952864
H	3.0576025	2.4473992	3.0575201
H	2.8789453	0.5978847	-5.0295586
H	-2.2733178	-0.6448973	5.6706588
H	-3.5929166	-0.2530158	-4.0537793
H	1.9225000	2.0771386	0.1908241
H	3.2238547	1.2592990	-1.8897885
H	-2.1980524	-2.8882425	3.4859090
H	-2.8911271	-1.5326670	-1.1574672
H	1.8848500	3.6242728	1.0933327

H	3.4191664	2.3801853	-3.2673678
H	-1.7676128	-2.9639080	5.2169680
H	-3.6088741	-2.3590667	-2.5736610
C	0.3586298	-2.3145500	-0.0689053
C	-0.6059152	-2.8878498	0.9727148
H	1.2871136	-2.9013998	-0.0561398
H	-0.0765343	-2.4881240	-1.0673960
H	-0.1484762	-2.8930401	1.9725766
H	-1.5468872	-2.3139098	1.0374224
H	1.1957755	-0.9035147	-0.0009287
H	1.6405799	-0.0882486	-0.0301560
H	-0.9070598	-3.9290654	0.7924551
